# Induction of Expandable Tissue-Specific Stem/Progenitor Cells through Transient Expression of YAP/TAZ

**DOI:** 10.1016/j.stem.2016.08.009

**Published:** 2016-12-01

**Authors:** Tito Panciera, Luca Azzolin, Atsushi Fujimura, Daniele Di Biagio, Chiara Frasson, Silvia Bresolin, Sandra Soligo, Giuseppe Basso, Silvio Bicciato, Antonio Rosato, Michelangelo Cordenonsi, Stefano Piccolo

**Affiliations:** 1 Department of Molecular Medicine, University of Padua School of Medicine, viale Colombo 3, 35126 Padua, Italy; 2 Department of Woman and Child Health, Haemato-Oncology Laboratory, University of Padua, via Giustiniani 3, 35128 Padua, Italy; 3 Center for Genome Research, Department of Life Sciences, University of Modena and Reggio Emilia, via G. Campi 287, 41100 Modena, Italy; 4 Istituto Oncologico Veneto IOV-IRCCS and Department of Surgery, Oncology, and Gastroenterology, University of Padua School of Medicine, via Gattamelata 64, 35128 Padua, Italy

## Abstract

The ability to induce autologous tissue-specific stem cells in culture could have a variety of applications in regenerative medicine and disease modeling. Here we show that transient expression of exogenous YAP or its closely related paralogue TAZ in primary differentiated mouse cells can induce conversion to a tissue-specific stem/progenitor cell state. Differentiated mammary gland, neuronal, and pancreatic exocrine cells, identified using a combination of cell sorting and lineage tracing approaches, efficiently convert to proliferating cells with properties of stem/progenitor cells of their respective tissues after YAP induction. YAP-induced mammary stem/progenitor cells show molecular and functional properties similar to endogenous MaSCs, including organoid formation and mammary gland reconstitution after transplantation. Because YAP/TAZ function is also important for self-renewal of endogenous stem cells in culture, our findings have implications for understanding the molecular determinants of the somatic stem cell state.

## Highlights

• YAP/TAZ expression turns differentiated mammary gland cells into mammary stem cells
• YAP-induced MaSCs form organoids and have reconstitution capacity
• Induction of YAP in differentiated fetal neurons yields tripotent neural stem cells
• Pancreatic exocrine cells are also converted to progenitors by YAP expression

## Graphical Abstract

**Figure:**
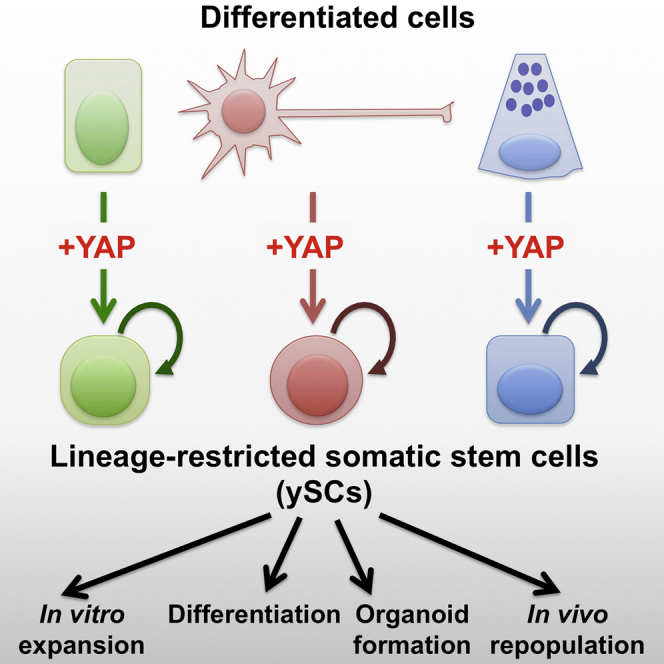

## Teaser

Reprogramming and lineage conversions have highlighted the plasticity of differentiated cell states. Here Panciera et al. build on these principles by showing that expression of YAP/TAZ can convert a range of differentiated cells into somatic stem cells of the same tissue, respecting lineage restrictions.

## Introduction

Stem cells (SCs) display the capacity to self-renew when they divide and to generate a differentiated progeny. Somatic SCs operate in multiple adult organs for continuous tissue renewal or repair after injury. However, these cells are still mainly defined by operational definitions and cell surface markers, rather than the molecular traits that govern their special status (Blanpain and Fuchs, 2014). Unlimited availability of normal, somatic SCs will be critical for effective organ repopulation in regenerative medicine applications to understand SC biology and for disease modeling in the Petri dish. However, these efforts remain limited by the fact that SCs are rare and difficult to purify or expand from native tissues.

Direct conversion of terminally differentiated cells back into their corresponding tissue-specific SCs may represent an attractive approach to obtain somatic SCs. Indeed, several reports have recently highlighted a surprising plasticity in somatic cell fates because differentiated cells can return to a SC status under special conditions, such as tissue damage (Blanpain and Fuchs, 2014). However, the identity of the factors able to control the somatic SC status remains poorly understood, limiting the exploitation of such plasticity.

Dysregulation of the Hippo signaling pathway has been recently associated with cell fate plasticity. In tumors, activation of the Hippo pathway transcriptional effectors YAP/TAZ can reprogram non-stem cancer cells into cancer SCs (Cordenonsi et al., 2011). Genetic inactivation of the Hippo cascade induces liver overgrowth, in part by influencing liver cell fate and zonation (Lee et al., 2016, Yimlamai et al., 2014, Fitamant et al., 2015). Nuclear YAP/TAZ proteins are found in anatomical locations enriched in tissue SCs, possibly as a consequence of their regulation by structural and chemical signals associated with the SC niche (Piccolo et al., 2014). That said, genetic ablation of YAP and/or TAZ from several adult organs in mice, such as liver, pancreas, intestine, and mammary gland, revealed that these factors are surprisingly dispensable during normal tissue homeostasis. Strikingly, however, in those same tissues, YAP/TAZ become essential for organ regrowth after tissue damage or oncogenic transformation (Azzolin et al., 2014, Zanconato et al., 2016). This suggests that YAP/TAZ remain latent—and, thus, apparently dispensable—in normal adult tissues but are called into action to generate new stem cells.

With this background in mind, we considered the possibility that ectopic expression of YAP or TAZ may be instrumental to turn differentiated cells into somatic stem-like cells. Here we tested this hypothesis in vitro by using distinct paradigms of terminal differentiation; that is, luminal mammary gland cells, neurons, and pancreatic exocrine cells. We found that transient expression of YAP/TAZ indeed converts differentiated cells into cells displaying multiple features of their corresponding tissue-specific SCs. Notably, the ability of YAP/TAZ to impart an SC state pairs with their endogenous function in isolated native SCs, where they are essential for preserving organoid-forming potential. Our work therefore reveals that a single factor can induce somatic stem cell features in cells of different lineages.

## Results

### YAP/TAZ Revert Differentiated Cells of the Mammary Gland into MaSC-like Cells

The mammary gland represents a classic model system for the study of epithelial SCs and tissue regeneration. Remarkably, implantation of mammary gland SCs (MaSCs) into the mammary fat pad is sufficient to regenerate an entire ductal tree, with MaSCs contributing to both the luminal and myoepithelial lineages (Blanpain and Fuchs, 2014). Given that YAP/TAZ can reprogram non-stem mammary tumor cells into their corresponding cancer stem cells (CSCs) (Cordenonsi et al., 2011), we hypothesized that expression of YAP/TAZ may bestow stem-like characteristics on normal mammary cells as well.

**Figure 1:**
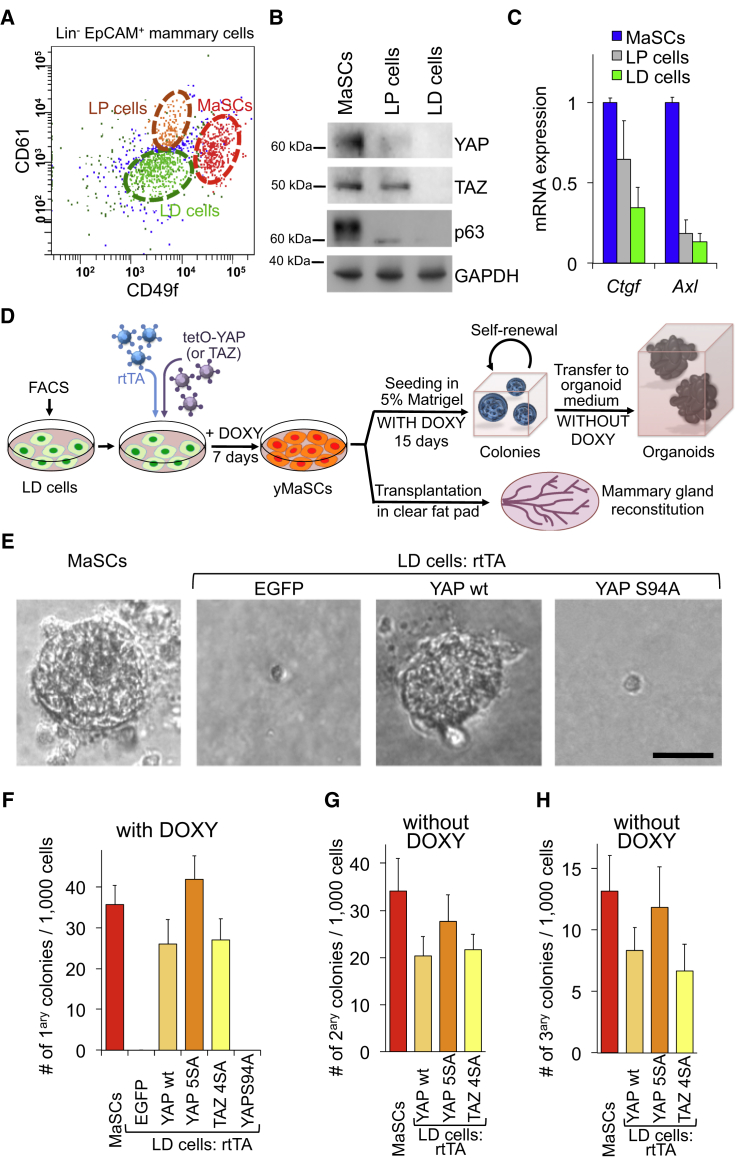
YAP and TAZ Convert Luminal Differentiated Cells in yMaSCs (A) FACS profile showing the distribution of Lin–/EpCAM+ mammary cells according to their CD49f/CD61 antigenic profile. Three subpopulations were separated: a MaSC-enriched fraction (EpCAM^low^CD49f^high^CD61^+^), luminal progenitors (EpCAM^high^CD49f^low^CD61^+^), and luminal differentiated cells (EpCAM^high^CD49f^low^CD61^−^). See also Figure S1A for the FACS pattern of EpCAM and Figures S1B–S1E for characterization of the three subpopulations. (B) Western blots for YAP, TAZ, and the MaSC marker p63 in the indicated purified populations. GAPDH served as a loading control. (C) qRT-PCRs for *Ctgf* and *Axl* in the indicated cell populations (mean + SD). The results are representative of three independent experiments (each using mammary glands from 20 mice) performed in triplicate. (D) Schematic of the experiments performed with LD cells. (E and F) Representative images (E) and quantifications (F) of mammary colonies formed by the indicated cells 15 days after seeding in mammary colony medium. The data in (F) are presented as mean + SD and are representative of five independent experiments, each with six technical replicates. (G and H) Quantifications of secondary (G) and tertiary (H) colonies formed by primary mammary colonies after dissociation and re-seeding in mammary colony medium without doxycycline. The data are representative of three independent experiments performed with six technical replicates and presented as mean + SD. See also Figure S1.

To address this, we used fluorescence-activated cell sorting (FACS) to isolate terminally differentiated luminal cells (LD, EpCAM^high^CD49f^low^CD61^−^) from dissected mouse mammary glands (Guo et al., 2012; Figure 1A). LD cells expressed markers of terminal differentiation and, in culture, were void of any proliferative potential and remained as single cells (Figures S1B–S1D). This differs from the MaSC-enriched or luminal progenitor (LP) fractions, which formed solid and cavitated colonies, respectively (Figure S1D). Endogenous YAP/TAZ proteins and their transcriptional targets *Ctgf* and *Axl* were highly expressed in the MaSC-containing population but at much lower levels in differentiated cells (Figures 1B and 1C).

To investigate whether ectopic expression of YAP or TAZ in LD cells could impart MaSC-like properties, FACS-purified LD cells were plated on collagen-coated dishes and transduced with doxycycline (Doxy)-inducible lentiviral vectors encoding for wild-type (WT) YAP or the activated versions of YAP and TAZ (i.e., YAP5SA or TAZ4SA, lacking inhibitory phosphorylation sites) (see the diagram in Figure 1D). As a control, cells were infected with an inducible EGFP vector. Transduced cells were cultured for 7 days in doxycycline-containing medium and then plated at clonogenic density in three-dimensional 5% Matrigel cultures (Experimental Procedures). Strikingly, cells expressing either YAP or TAZ formed solid colonies indistinguishable from those generated by MaSCs (Figures 1E and 1F) and very distinct from the cysts generated by LP cells (Figure S1D). EGFP-expressing control cells invariably remained as single cells without ever originating even a single colony in 33 experiments. As a further control, the expression of transcriptionally deficient YAPS94A (i.e., unable to interact with its DNA-binding partner TEAD) also had no effect.

We then asked whether YAP/TAZ expression converted luminal differentiated cells to a MaSC-like state. This includes the ability to form colonies that can be serially passaged. Indeed, YAP/TAZ-induced colonies, similarly to those generated from MaSCs, could form additional generations of colonies after single-cell dissociation (Figures 1G and 1H). Notably, colonies could be passaged even after expression of ectopic YAP had been turned off (by removing doxycycline) (Figures 1G, 1H and S3A). This suggests that transient expression of YAP/TAZ is sufficient to stably endow self-renewal potential to differentiated mammary cells. We thus designated the YAP/TAZ-induced “MaSC-like” cells as “yMaSCs.”

To verify whether the switch from LD to yMaSC could be recapitulated at the single-cell level, individual LD cells were seeded in 96-well plates (visually verified) and induced to express YAP. By monitoring the resulting outgrowths, we found that these individual cells formed solid colonies with high frequency (Figure S1F; 18.5% on average in the three independent experiments). From this experiment, we also noticed that this frequency of conversion, combined with the lack of colony-forming cells in controls (0%), argues against the hypothesis that yMaSCs arise from rare, contaminating, pre-existing stem/progenitors in our LD preparations.

Of note, we also found that overexpressing YAP in the endogenous MaSC-enriched cell population does not increase its colony-forming capacity (Figure S1G). In other words, even if rare contaminant MaSCs were present, then these would remain rare and not be expanded by YAP expression.

### Validation of LD-to-yMaSC Conversion by Lineage Tracing

**Figure 2:**
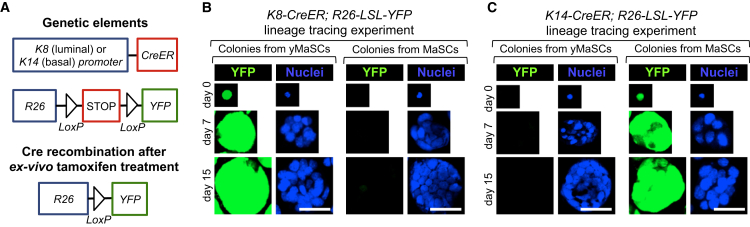
Lineage Tracing of yMaSCs (A) Schematic of the genetic lineage tracing strategies to trace different mammary cell lineages. (B and C) Immunostainings with anti-YFP of K8-CreER/R26-YFP-traced (B) or K14-CreER/R26-YFP-traced (C) cells during colony formation. Days indicate the time in mammary colony medium. Scale bars, 62 μm. See also Figure S2.

To validate the notion that YAP expression converts differentiated cells to an SC fate, we carried out reprogramming of LD cells purified from *K8-CreERT2; R26-LSL-YFP* mice (Figure 2A), allowing for a lineage tracing strategy to genetically label luminal cells (Van Keymeulen et al., 2011). For this experiment, we first FACS-purified LD cells (as in Figure 1A). After plating, cells were exposed to a pulse of tamoxifen to activate the YFP tracer exclusively in K8-positive cells and then infected with empty or YAP-expressing vectors. Colonies generated by YAP reprogramming of LD cells were entirely YFP-positive, confirming their origin from the luminal lineage (Figures 2B and S2A). As a control, we validated that the K8-CreERT2 tracing was restricted to luminal cells. Tamoxifen-treated MaSCs from the *K8-CreERT2; R26-LSL-YFP* mammary gland formed colonies that were exclusively YFP-negative (n = 154, 0% YFP+) (Figure 2B). These results also argue against the possibility that YFP-labeled yMaSCs could emerge from contaminating endogenous MaSCs.

The same conclusion was further validated by a complementary experiment in which we genetically labeled the basal/MaSC-enriched cell population by using *K14-CreERT2; R26-LSL-YFP* mice (Van Keymeulen et al., 2011; Figure 2A). LD cells and MaSCs were sorted by FACS as above and then treated with a pulse of tamoxifen to label K14-positive cells. YAP-reprogrammed LD cells purified from this genetic setup generated yMaSC colonies that were invariably void of any YFP expression (0%, n = 122) (Figure 2C). As a positive control, endogenous MaSCs traced by *K14-CreERT2* labeling formed YFP-positive colonies (Figure 2C). Collectively, several lines of evidence indicate that yMaSCs do not emerge from rare contaminating MaSCs pre-existing in our LD preparation. We conclude from these lineage-tracing experiments that YAP acts in differentiated cells to reprogram them into a MaSC-like state.

### The Expansion, Differentiation, and Regenerative Potential of yMaSCs

**Figure 3:**
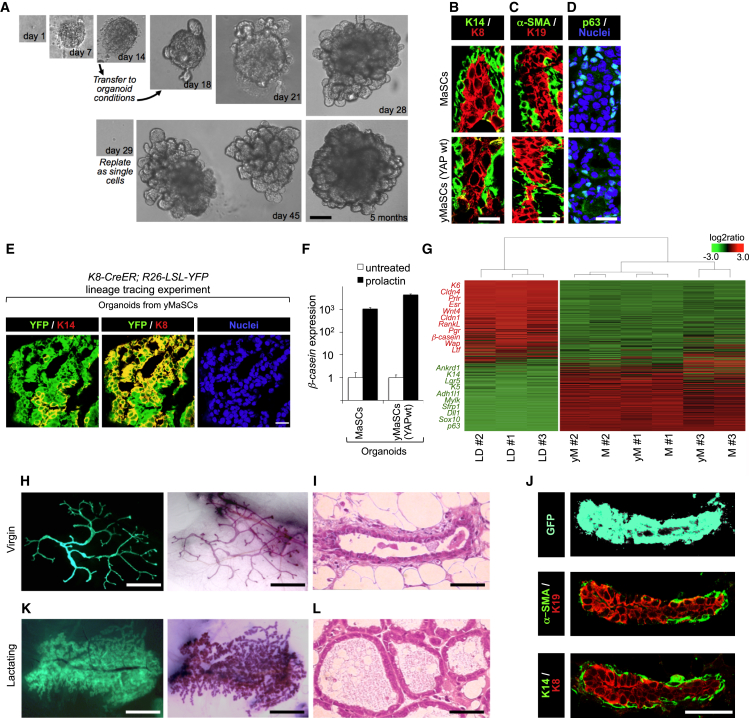
Characterization of yMaSCs (A) Representative images of yMaSC outgrowths at the indicated time points. Until day 14, cultures were in mammary colony medium. After transfer to organoid conditions (see scheme in Figure 1D), 64%–75% of yMaSC colonies evolved as organoids and were maintained and passaged without doxycycline. Scale bar, 250 μm. See also Figure S3A for transgene expression. (B–D) Organoids from MaSCs and yMaSCs (from YAP WT) expressed basal and/or stem (α-SMA, K14, p63) and luminal markers (K8, K19). Scale bars in IF pictures, 17 μm). See also Figures S3D–S3F for yMaSCs from phosphomutant YAP/TAZ. (E) Immunostainings with anti-YFP combined with either anti-K14 or anti-K8 antibodies of yMaSC-derived organoids obtained from K8-CreER/R26-YFP-traced LD cells as in Figure 2A and Figure S2A. Scale bars, 49 μm. (F) Organoids from MaSCs and yMaSCs (from YAP WT) expressed *β-casein* (qRT-PCR) when treated with prolactin. Data were normalized to *Gapdh* expression and are presented as mean + SD. The results are representative of two independent experiments performed in triplicate. See also Figure S3G. (G) Unsupervised hierarchical clustering of gene expression profiles in LD cells, organoids from MaSCs (M), and organoids from yMaSCs (yM). Each column represents one separated biological sample. Genes are ordered according to the decreasing average expression level in LD cells. Representative genes upregulated in LD cells (red) or in MaSC- and yMaSC-derived organoids (green) are shown on the left. (H–J) Mammary gland reconstitution generated by stably GFP-expressing yMaSCs (from YAP WT) in virgin females. (H) Whole-mount images (left, native GFP fluorescence; right, hematoxylin staining). (I) Histological section. (J) Representative sections stained for GFP and the indicated markers. See Figures S3I and S3J for controls. Scale bars, 0.5 cm in (H) and 21 μm in (I) and (J). (K and L) Mammary gland reconstitution generated by yMaSC organoids in an impregnated female. (K) Whole-mount images (left, native GFP fluorescence; right, hematoxylin staining). (L) Histological section. Note that, upon gestation and lactation, the mammary gland is constituted by alveoli filled with milk. Scale bars, 0.5 cm in (K) and 21 μM in (L). See also Figure S3.

We then examined whether yMaSCs are functionally equivalent to mammary SCs, as determined by additional characteristic properties of SCs, such as the ability to self-organize in vitro into mammary tissue-like structures, to differentiate along distinct lineages, and to regenerate a mammary tree in vivo after injection into a cleared mammary fat pad. For this, we sought to establish a long-term culture system that allows mammary SCs to form mammary gland-like structures in vitro. MaSC- and yMaSC-derived colonies were transferred and embedded into 100% Matrigel and overlaid with “organoid” medium (Sato et al., 2009) in the absence of doxycycline. Under these conditions, colonies underwent extensive budding and, by 2 weeks, grew into large epithelial organoids (Figure 3A; Figures S3B and S3C; see the legend of Figure 3A for quantification). Organoids derived from yMaSCs were indistinguishable in growth pattern, size, and frequency from those generated by natural MaSCs. yMaSC-derived organoids were dissociated, replated as single cells every 2 weeks, and cultured for at least 12 months (i.e., >25 passages) without changes in growth pattern, plating efficiency, and differentiation potential.

By histological examination, both MaSC and yMaSC organoids were composed by a stratified epithelium reminiscent of the histology of the mammary gland (E-cadherin-positive; Figure S3C). Internal cells surrounding a lumen-like cavity expressed differentiated luminal markers such as K8 and K19 (Figures 3B and 3C). Outer cells (either at external surfaces or bordering inner folds) displayed expression of basal, myoepithelial, and SC markers (K14, α-smooth muscle actin [α-SMA], p63) (Figures 3B–3D). yMaSCs derived from *K8-CreERT2; R26-LSL-YFP-*traced LD cells (as in Figure 2B) generated organoids with YFP-positive and K14-positive basal cells (Figures 3E and S2A), attesting to their origin from reprogrammed LD cells. Furthermore, addition of a lactogenic stimulus triggered expression of *α-* and *β-casein*, indicative of alveolar (milk-producing) cell differentiation (Figures 3F and S3G).

To molecularly characterize yMaSCs, we FACS-purified yMaSCs from organoids and evaluated the expression of luminal and basal and/or stem cell markers (Figure S3H). yMaSCs were remarkably similar to native MaSCs (freshly purified from the mammary gland) because they express basal markers (including myoepithelial markers such as *α-Sma* and *Myh11*) but not luminal markers (*Claudin1*, *K8*, *K18*, and *K19*). yMaSCs also express genes previously associated with various types of adult mammary SCs, including *ΔNp63*, *Lgr4/5/6*, and *Procr*, and all to levels comparable with MaSCs (Chakrabarti et al., 2014, Plaks et al., 2013, Wang et al., 2015).

By unsupervised hierarchical clustering of gene expression profiles, organoids from MaSCs or yMaSCs could not be distinguished (Figure 3G). Taken together, the results indicate that, similar to authentic MaSCs, yMaSCs display self-renewal potential, generate self-organizing epithelial structures reminiscent of the normal mammary gland, and retain multilineage differentiation ability.

Next we tested whether yMaSCs displayed mammary gland reconstituting activity. For this, FACS-purified LD cells were transduced with vectors encoding for EGFP and inducible wild-type YAP. Cells were treated with doxycycline for 7 days and then transplanted (10^3^–10^4^ cells) into the cleared mammary fat pads of non-obese diabetic (NOD)-severe combined immunodeficiency (SCID) mice. Strikingly, cells that had experienced transient expression of wild-type YAP had also acquired the ability to regenerate the mammary gland (25%, n = 16) (Figures 3H and 3I). Ductal tree and terminal end buds were regenerated when as few as 100 YAP-reprogrammed LD cells were implanted (33%, n = 6). As a control, LD cells transduced with the sole EGFP vector did not display any reconstituting activity at any inoculum dose (0%, n = 28, 10^2^–5 × 10^4^) (Figure S3I). Histological analyses revealed that the epithelial outgrowths obtained from yMaSCs were EGFP-positive and morphologically indistinguishable from those generated by endogenous MaSCs and consisted of a bilayered epithelium composed of a basal and/or myoepithelial layer (positive for K14 and α-SMA) overlaid by luminal cells (positive for K8 and K19) (Figures 3J and S3J).

To explore the reconstituting potential of a single yMaSC, we injected in the cleared fat pads single-cell derived organoids and found that these were also able to regenerate the mammary gland (33%, n = 6). Notably, when these mice were impregnated, reconstituted mammary glands generated a dense ductal system ending in clusters of milk-secreting alveoli, indicating that yMaSCs retain full differentiation potential in vivo (Figures 3K and 3L). We conclude from this collective set of experiments that transient expression of YAP/TAZ in differentiated cells of the mammary gland is able to convert them into bona fide MaSCs.

YAP/TAZ are not only instrumental for reprogramming of differentiated mammary cells but also endogenously required in MaSCs for preserving their self-renewal potential. Indeed, we generated organoids from either endogenous MaSCs or yMaSCs obtained from *Yap*^fl/fl^*; Taz*^fl/fl^ mice; deletion of endogenous YAP/TAZ by Adeno-Cre severely affected the ability of organoids to self-renew upon passaging (Figure S3K). Consistently, we also found that conversion to the ySC state was accompanied by activation of endogenous YAP/TAZ proteins. As shown in Figure S3L, induction of exogenous YAP in LD cells turned on expression of endogenous YAP and TAZ that remained expressed in ySC-derived organoids after ectopic YAP expression had been turned off (see Figure S3A for on-off control of exogenous YAP expression). We conclude that transient exposure to YAP/TAZ is sufficient to empower a self-sustaining loop of endogenous YAP/TAZ expression.

### YAP Turns Neurons into Neural SC-like Cells

**Figure 4:**
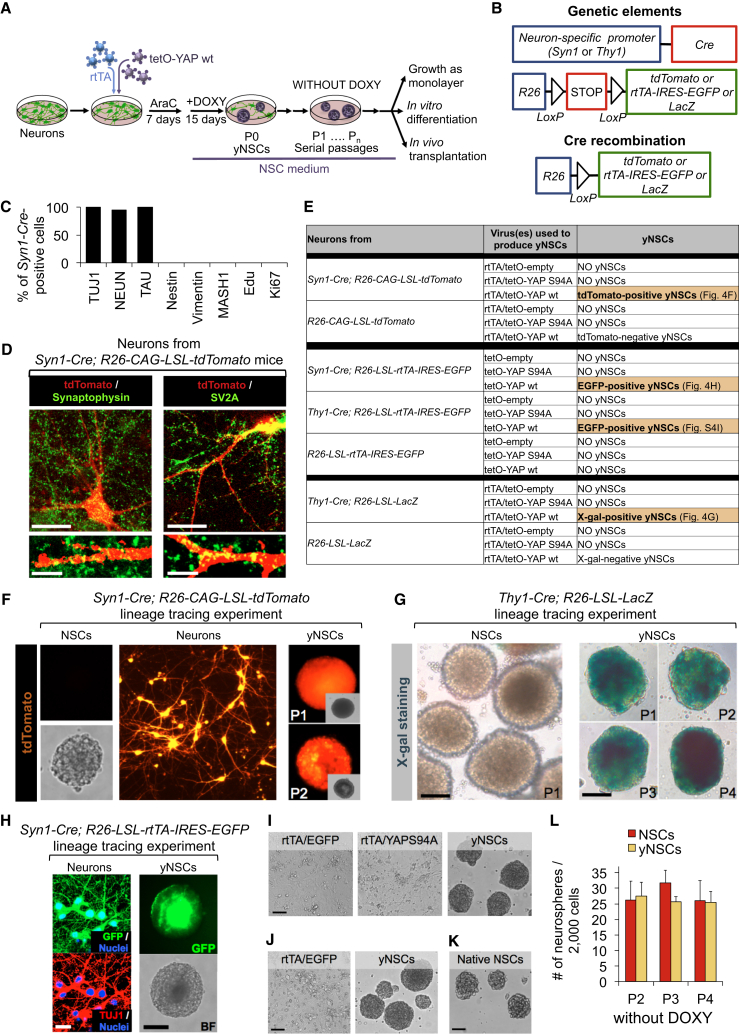
YAP Converts Neurons in yNSCs (A) Schematic of the experiments performed with hippocampal or cortical neurons. (B) Schematic of the genetic lineage tracing strategy used to trace neurons ex vivo. (C) We confirmed, in primary neuronal cultures prepared from the brain of *Syn1-Cre; R26-LSL-rtTA-IRES-EGFP* or *Syn1-Cre; R26-CAG-LSL-tdTomato* transgenic mice, that expression of the lineage tracer is indeed restricted to post-mitotic neurons expressing TUJ1 (100%, n = 635 cells), TAU (100%, n = 140 cells), and NEUN (95%, n = 252 cells). None of these traced cells were ever positive for the NSCs and/or progenitor markers Nestin, Vimentin, or MASH1 or for proliferation markers (5-Ethynyl-2’-deoxyridine [EdU], Ki67) (0%, n > 4,000 cells). See Figure S4F for representative images. See also Figure S4G for similar characterizations on *Thy1-Cre*-traced neuronal preparations. (D) Immunofluorescence for the synaptic markers Synaptophysin and SV2A in cortical neurons derived from *Syn1-Cre; R26-CAG-LSL-tdTomato* mice. Scale bars, 11 μm (top) and 5.7 μm in magnifications (bottom). (E) Table summarizing the results obtained in different lineage tracing experiments. (F) Lineage tracing experiment showing that yNSCs originate from neurons. The images are bright-field and tdTomato-fluorescence pictures of primary cortical neurons from *Syn1-Cre; R26-CAG-LSL-tdTomato* mice and yNSCs derived from them (at the indicated passages). Neurospheres from *Syn1-Cre; R26-CAG-LSL-tdTomato* NSCs are presented as a negative control. (G) Panels are X-gal stainings of yNSCs derived from hippocampal neurons from *Thy1-Cre; R26-LSL-LacZ* mice (scale bars, 210 μm) at the indicated passages. Neurospheres from *Thy1-Cre; R26-LSL-LacZ* NSCs (scale bar, 210 μm) are presented as a negative control. (H) Lineage tracing experiment with the *Syn1-Cre* driver showing that yNSCs originate from Syn1-traced neurons. Left: immunostaining for GFP and TUJ1 in cortical neurons obtained from *Syn1-Cre; R26-LSL-rtTA-IRES-EGFP* mice. Right: bright-field and GFP fluorescence pictures of neurospheres derived from yNSCs obtained from the same neurons after transduction with doxycycline-inducible YAP. See also Figures S4G–S4I for similar results with the alternative lineage tracing strategy using *Thy1-Cre; R26-LSL-rtTA-IRES-EGFP* mice. (I–K) Representative images of yNSCs neurospheres (second passage, P2) derived from hippocampal (I) or cortical (J) neurons. Images from negative control transduced neurons are shown as a reference (I and J). Neurospheres from native NSCs are presented for comparison (K). Scale bars, 210 μm. (L) P1 yNSCs were dissociated to single cells and replated at clonal density for neurosphere formation in the absence of doxycycline for further passages (P2, P3, and P4). Native NSCs are presented for comparison. Graphs are quantifications of neurospheres formed by the indicated cells. Results are representative of at least eight (P2), six (P3), and three (P4) independent experiments performed in six replicates. Data are presented as mean + SD. See also Figure S4.

**Figure 5:**
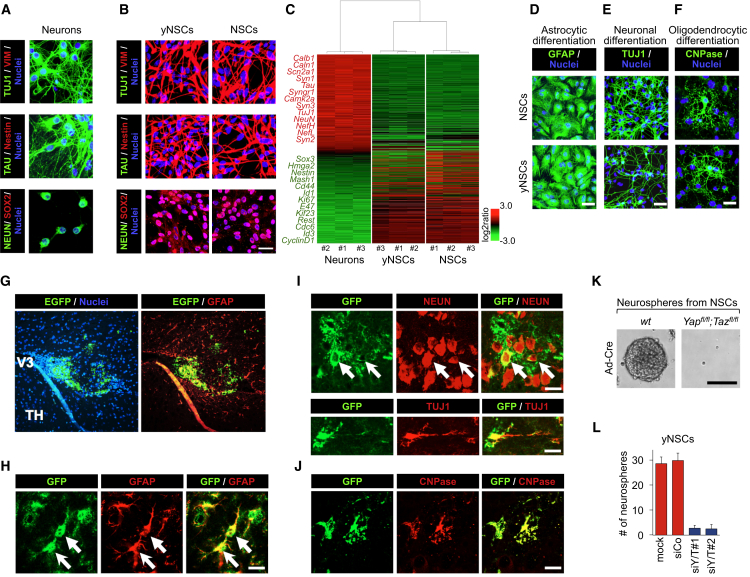
Characterization of yNSCs (A and B) Immunofluorescence for the indicated markers in hippocampal neurons (A) and P3 yNSCs plated as a monolayer (B). Endogenous NSCs served as a positive control (scale bar, 23 μm). As shown in Figure S5B, Nestin-, SOX2-, and Vimentin-positive yNSCs derived from *Thy1-Cre; R26-LSL-rtTA-IRES-EGFP* neuronal preparations are co-stained with EGFP, confirming their origin from differentiated cells. (C) Unsupervised hierarchical clustering of gene expression profiles in cortical neurons, yNSCs, and NSCs. Each column represents one separated biological sample. Genes are ordered according to the decreasing average expression level in neurons. Representative genes upregulated in neurons (red) or in NSCs and yNSCs (green) are shown on the left. (D–F) YAP-induced yNSCs and endogenous NSCs as a positive control were plated and differentiated toward an astrocytic, neuronal, or oligodendrocytic fate (Experimental Procedures). Confocal images showing the astrocytic marker GFAP (D), the neuronal differentiation marker TUJ1 (E), and the oligodendrocytic marker CNPase (F) are displayed. The results are representative of three independent experiments performed in triplicate. Scale bars, 50 μm. For neuronal differentiation, see also Figures S5C–S5E, attesting acquisition of TAU and loss of Nestin expression. (G–J) yNSCs were transduced with a constitutive EGFP-expressing vector and injected into the brains of recipient mice. Four weeks later, the brains were fixed and processed for immunofluorescence analyses. (G and H) Representative confocal images showing that injected yNSCs (GFP-positive) integrate in the brain parenchyma and massively differentiate into an astrocytic fate, as indicated by GFAP expression (arrows). V3, third ventricle; TH, thalamus. (I and J) Representative confocal images showing injected yNSCs (GFP-positive) differentiated in NEUN- and TUJ1-positive neurons (I, arrows) or CNPase-positive oligodendrocytes (J). Scale bars, 19 μm. (K) Representative images of neurospheres from WT or *Yap*^fl/fl^*; Taz*^fl/fl^ NSCs transduced with Ad-Cre. Scale bar, 250 μm. (L) yNSCs (passage 4 as neurospheres) were dissociated, plated on fibronectin-coated dishes, and transfected with the indicated siRNAs. The graph represents the quantification (mean ± SD) of neurospheres derived from the indicated cells. See also Figure S5.

Neurons are post-mitotic cells that have been long considered refractory to any cell fate change. Endogenous YAP and TAZ proteins are highly expressed and transcriptionally active in neural stem cells (NSCs) but absent in neurons (Figures S4A–S4E), prompting us to determine whether ectopic expression of YAP in neurons was sufficient to convert them into NSCs. Primary cultures enriched in post-mitotic neurons (Figures 5A and S5A; see below) were obtained from the hippocampus or cortex from late mouse embryos (embryonic day 19 [E19]) or newborns. Cells were cultured in serum-free neuronal differentiation medium (Neurobasal with B27 containing vitamin A) also containing cytosine β-D-arabinofuranoside (AraC) for 7 days to support neuronal differentiation and eliminate proliferating cells (Figure 4A).

To follow the fate of post-mitotic neurons during YAP reprogramming, we adopted redundant lineage tracing strategies to stably label post-mitotic neurons. For this we prepared primary neurons from the brain of *Syn1-Cre* or *Thy1-Cre* transgenic mice expressing the Cre recombinase exclusively in post-mitotic neurons under the control of a neuron-specific promoter (Zhu et al., 2001, Dewachter et al., 2002) also bearing Cre-inducible tracers from the R26 locus (Figure 4B). We validated that expression of the lineage tracer was restricted to cells displaying unambiguous neuronal morphology and markers, whereas none of these traced cells was ever positive for NSCs and/or progenitor markers, as detailed in Figures 4C, 4D, and 4H and Figures S4F–S4H.

Primary cells were infected with lentiviral vectors encoding for reverse tetracycline transactivator (rtTA) and inducible wild-type YAP (Experimental Procedures). After AraC, neurons were shifted to NSC medium in the presence of doxycycline (see experimental outline in Figure 4A). Remarkably, after 2 weeks, neurosphere-like structures emerged from YAP-expressing neurons (P0 spheres) but never from neurons transduced with rtTA alone or rtTA combined with empty vectors or transcriptionally inactive YAPS94A (Figure 4E). Lineage-traced neurons from *Syn1-Cre; R26-CAG-LSL-tdTomato* or *Thy1-Cre; R26-LSL-LacZ* transgenic mice gave rise to tdTomato-positive or β-galactosidase (β-gal)-positive neurospheres, respectively (Figures 4F and 4G). Similarly, infection with tetO-YAP lentiviruses of neurons from mice bearing *Syn1-Cre* or *Thy1-Cre* and the *R26-LSL-rtTA-IRES-EGFP* reporter generated EGFP-positive yNSCs (Figures 4H and S4G–S4I). In contrast, NSCs derived from the same strains were invariably unlabeled (Figures 4F, left panel, and 4G, left panel). This confirms that, as originally described (Zhu et al., 2001, Dewachter et al., 2002), these drivers are not active in natural NSCs; hence, YAP-induced NSCs, or “yNSCs,” originate from neurons rather than through amplification of pre-existing, contaminating, endogenous NSCs.

By monitoring the process more closely using lineage-traced neurons from *Syn1-Cre; R26-CAG-LSL-tdTomato,* we found that Doxy addition triggered YAP expression in about 30% of these neurons, and that, of these, 25% became positive for the NSC marker Nestin 2–3 days after switching them to the NSC medium (Figure S4J). This frequency of conversion, combined with the fact that no Nestin-positive neurons were present before YAP induction and no Nestin-positive neurons ever appeared in control cultures, argues against the possibility that yNSCs arose from rare pre-existing stem/progenitor cells in our neuronal preparations. This is in line with the above conclusions drawn from lineage tracing experiments.

Of note, we also found that overexpressing YAP in endogenous NSCs does not increase neurosphere-forming capacity (Figure S4K). Thus, even if rare contaminant NSCs were present, then it seems these would remain rare and not be expanded by YAP expression.

P0 spheres of yNSCs were transferred to new plates for further growth and could then be propagated for several passages as clonal outgrowths after single-cell dissociation, similarly to native NSCs (Figures 4I–4L). Lineage tracing was retained upon passaging (Figures 4F and 4G), indicating that YAP-reprogrammed neurons had acquired self-renewing properties.

The propagation of yNSCs as neurospheres did not require addition of doxycycline, indicating that transient exposure to exogenous YAP is sufficient to induce self-renewal properties that are autonomously maintained. In line, as shown by experiments with a Cre-excisable tetO-YAP lentiviral vector, post-reprogramming deletion of the whole YAP-encoding viral cassette had no effects on yNSC maintenance (Figures S4L–S4N).

### Characterization of yNSCs

Next we characterized yNSCs by marker gene expression using immunofluorescence, qRT-PCR, and gene profiling. As shown in Figures 5A and 5B and Figures S5A and S5B, yNSCs completely lost expression of the terminal differentiation markers present in the original neurons (such as TUJ1, TAU, and NEUN) and, instead, expressed high levels of NSC markers (Nestin, SOX2, and Vimentin). Furthermore, we compared the transcriptome of parental neurons, yNSCs, and native NSCs and found that yNSCs completely lost their neuronal identity and acquired a gene expression profile closely similar to native NSCs (Figure 5C).

Neural SCs are defined as tripotent, as attested to by their ability to differentiate in astrocytes, neurons, and oligodendrocytes. We therefore examined the differentiation potential of yNSCs by placing them under appropriate culture conditions (Experimental Procedures). yNSCs could differentiate into astrocytes, neurons, and oligodendrocytes as defined by markers and morphology (Figures 5D–5F and S5C–S5E) and thus are indeed tripotent. To investigate the in vivo differentiation potential of yNSCs, we transplanted EGFP-labeled yNSCs in the brain of newborn mice (n = 5). Four weeks after transplantation, grafted yNSCs invariably lost Nestin positivity (Figure S5F) and mainly remained close to the injection site, where they primarily acquired expression of GFAP, indicative of astrocyte differentiation (Figures 5G and 5H). Injected yNSCs also differentiated into NEUN- and TUJ1-positive neurons or CNPase-positive oligodendrocytes (Figures 5I and 5J). Importantly, no tumor formation was ever observed after histological examination of the yNSCs-injected brain parenchyma. Thus, YAP induces conversion of neurons into cells that have functional properties similar to those of normal NSCs.

As for MaSCs, endogenous YAP/TAZ are essential to sustain the expansion of native NSCs in vitro because ex-vivo Adeno-Cre-mediated deletion of YAP/TAZ from *Yap*^fl/fl^*; Taz*^fl/fl^ NSCs blunted neurosphere formation (Figure 5K). We further established that the self-renewal properties of yNSCs are also sustained by reactivation of endogenous YAP/TAZ. Two lines of evidence support this conclusion. First, endogenous TAZ is induced in yNSCs and remains as such after doxycycline withdrawal and Cre-mediated excision of the tetO-YAP cassette (Figure S5G). Second, YAP/TAZ depletion in yNSCs by transfecting independent pairs of small interfering RNAs (siRNAs) greatly impairs their self-renewal properties (Figure 5L). These results raise an interesting parallel between the requirement of YAP/TAZ in native NSCs and induced yNSCs.

### Ex Vivo Generation of Pancreatic Progenitors from Exocrine Cells

**Figure 6:**
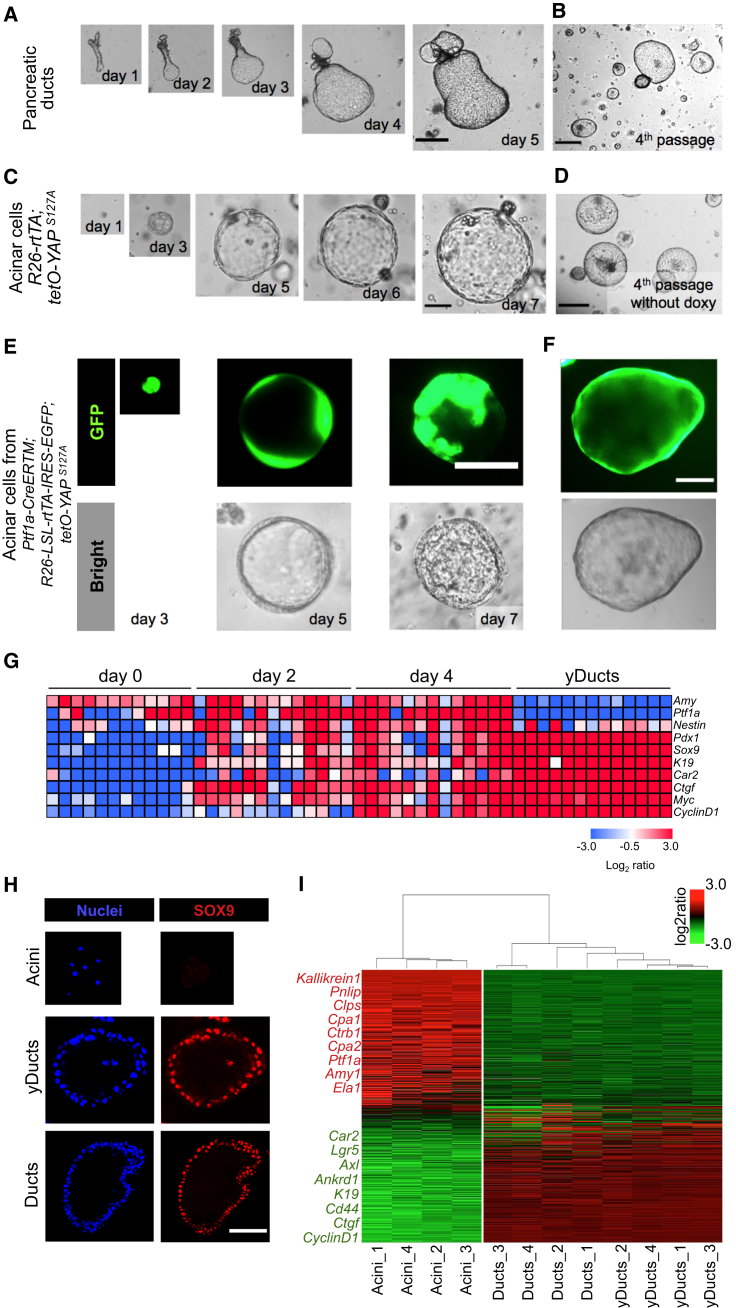
YAP Converts Pancreatic Acinar Cells to Duct-like Organoids (A and B) Representative images of a pancreatic duct fragment growing in pancreatic organoid medium at the indicated times (A) and after four passages in fresh Matrigel (B). Pictures are representative of three independent experiments performed with four technical replicates. Scale bars, 290 μm. (C and D) Serial images of a single acinar cell derived from *R26-rtTA; tetO-YAP*^S127A^ growing as cyst-like organoids at the indicated time points after Doxy addition (C) and after four passages in fresh Matrigel in the absence of Doxy (D). Pictures are representative of five independent experiments performed with four technical replicates. Scale bars, 70 μm in (C) and 290 μm in (D). (E and F) Lineage tracing experiments using the *Ptf1a-CreERTM* driver. Images are bright-field and GFP fluorescence pictures of transgenic YAP-expressing exocrine cells at the indicated time points of Doxy treatment (E) and after passaging in absence of Doxy (F). See also Figure S6I for a schematic of the experiment. Scale bars, 70 μm in (E) and 130 μm in (F). (G) Single-cell gene expression profile of pancreatic cultures of the *R26-rtTA; tetO-YAP*^S127A^ genotype during the YAP-induced conversion of acinar cells to yDucts. Rows are evaluated genes, and columns are individual cells. Day 0, starting acini (without doxycycline); day 2, cultures that experienced 48 hr of doxycycline; day 4, cultures that experienced 96 hr of doxycycline. The heatmap represents expression levels as log2 ratio normalized to *18S* rRNA. (H) Organoids from duct fragments (Ducts, bottom, as in B) and YAP-induced organoids (yDucts, center) expressed the ductal marker SOX9 and were negative for the exocrine marker Amylase (data not shown) by immunofluorescence. Acinar cells (top) are shown as a control. Scale bar, 80 μm. (I) Unsupervised hierarchical clustering of gene expression profiles in acini, yDucts, and Ducts. Each column represents one separated biological sample. Genes are ordered according to the decreasing average expression level in acini. Representative genes upregulated in acinar cells (red) or in Ducts and yDucts (green) are shown on the left. See also Figure S6.

Pancreatic progenitors are rare in the normal pancreas but can be regenerated by differentiated acinar exocrine cells upon injury (Pan et al., 2013). Pancreatic progenitors are expandable in vitro as ductal organoids (Huch et al., 2013; Figures 6A and 6B), and, like MaSCs and NSCs, display nuclear and transcriptionally active YAP/TAZ and genetically require YAP/TAZ for their propagation (Figures S6A–S6C). Considering the intrinsic plasticity of acinar cells, we used them as a third reprogramming paradigm, asking whether a transient pulse of YAP expression could be sufficient to convert them into progenitors. To this end, we isolated pancreatic acini from *R26-rtTA; tetOYAP*^S127A^ adult mice and dissociated them to obtain a single cell preparation. Cells were plated in 100% Matrigel and cultured in the presence of doxycycline in pancreas organoid medium. In just a few days, acinar cells induced to express YAP, but not cells left without doxycycline, expanded as cyst-like organoids (Figures 6C and S6D). Acinar cells derived from control *R26-rtTA* mice remained as single cells or, more rarely, formed small cysts but never expandable organoids (Figure S6D). After initial derivation, YAP-induced organoids (or “yDucts”) could be passaged for several months even in the absence of doxycycline and, thus, in the absence of exogenous YAP/TAZ (for at least 10 passages, 6 months). Individual organoids could be manually picked and expanded as clonal lines. By morphology, size, and growth pattern, organoids derived from converted acinar cells were comparable with those obtained from handpicked pancreatic duct fragments after whole pancreas dissociation (Huch et al., 2013; Figures 6B and 6D).

As an alternative strategy, we embedded whole pancreatic acini explanted from *R26-rtTA; tetOYAP*^S127A^ in collagen and cultured them under low-serum conditions known to preserve acinar cell identity ex vivo (Means et al., 2005). When treated with doxycycline to induce YAP expression (Figure S6E), pancreatic acini converted within a few days to ductal organoid structures and with high efficiency (>70%) (Movies S1 and S2; Figures S6F–S6H). As a control, acini lacking exogenous YAP expression (e.g., left without doxycycline; Figure S6H) remained as such and never converted to organoids. After transfer to 100% Matrigel-pancreatic organoid medium, the YAP-induced ducts, but not control acini, regrew into organoids and could be maintained for several passages after single-cell dissociation even in the absence of doxycycline (Figure S6G).

To validate that yDucts were indeed derived from differentiated exocrine acinar cells, we carried out genetic lineage tracing experiments using *Ptf1a-CreERTM; R26-LSL-rtTA-IRES-EGFP; tetO-YAP*^S127A^ mice (Figure S6I). In this genetic background, tamoxifen treatment of adult mice causes irreversible genetic tracing exclusively of pancreatic acinar cells (but not of endocrine, ductal, or centroacinar cells), as reported previously by others (Pan et al., 2013) and revalidated here (Figure S6J). After treatment, mice were kept without tamoxifen for 1 week, and then pancreata were explanted to prepare whole acini or single acinar cells that were cultured as above (see experimental outline in Figure S6I). These EGFP-positive cells never formed any organoid in the absence of doxycycline (Figures S6K and S6L). Instead, doxycycline-induced YAP expression caused the formation of expandable yDucts, that retained EGFP positivity over passaging, formally demonstrating their derivation from terminally differentiated exocrine cells (Figures 6E, 6F, S6M, and S6N; Movie S3). As a control of driver specificity in our culture conditions, organoids derived from endogenous ductal progenitors explanted from *Ptf1a-CreERTM; R26-LSL-rtTA-IRES-EGFP; tetO-YAP*^S127A^ pancreata were never labeled by EGFP (Figure S6O). This indicates that EGFP-traced yDucts emerge from exocrine cells and not from pre-existing ductal progenitors. In line with this conclusion, YAP overexpression is inconsequential in endogenous ductal progenitors for their clonogenic and organoid forming ability (Figure S6P), making unlikely the possibility that YAP expression might expand rare pre-existing contaminants.

We also performed a time course analysis of gene expression dynamics occurring at the single-cell level during reprogramming induced by tetO-Yap. As shown in Figure 6G, at the beginning of the experiment, cells expressed exocrine markers (*Amy* and *Ptf1a*) but not markers of pancreatic progenitors or ductal/centroacinar cells (*Pdx1*, *Sox9*, *K19*, and *Car2*), cell proliferation (*CyclinD1*), or the YAP targets *Ctgf* and *Myc* (Zanconato et al., 2015). After 2 days of doxycycline treatment, pancreatic progenitor markers were turned on in most cells and did so more robustly on day 4 and then in yDuct cells (in line with what is shown in Movies S1, S2, and S3). On days 2 and 4 (but not in yDucts), most acinar cells retained concomitant expression of acinar markers and could thus be considered cells caught in transition. We noticed that progenitor markers were already present on day 2, before cells acquired expression of the proliferation marker *CyclinD1*, indicating that phenotypic conversion into a ductal progenitor state can be initially uncoupled from proliferation.

In section, yDuct-derived organoids appeared as epithelial monolayers surrounding a central cavity (Figure 6H). By qRT-PCR and immunofluorescence, organoids lost markers of exocrine differentiation (*Ptf1a*, *α-amylase*, *Elastase*, and *CPA1*) and acquired expression of ductal markers (*K19*, *Sox9*, *Hes1*, and *Cd44*) and proliferative markers (*cMyc* and *CyclinD1*), all to levels comparable with those of native ductal organoids (Figures 6H and S6Q). To determine the extent of YAP-induced conversion of acinar cells and their molecular overlap with native ductal progenitors, we carried out transcriptomic analyses. As shown in Figure 6I, yDucts diverged from parental acinar cells to become ostensibly similar to bona fide pancreatic progenitors. Under differentiating conditions, yDuct-derived cells could be induced to re-express the differentiated exocrine marker CPA1 and to downregulate K19 (Figure S6R). When transplanted into the pancreas of NOD-SCID mice, yDucts remained as such and never formed any tumor (n = 6, data not shown), indicating that yDucts are indeed non-transformed and non-tumorigenic. Together, the results indicate that exocrine cells with a history of exposure to YAP acquired key molecular and biological features of ductal pancreatic progenitors.

## Discussion

Here we report that expression of a single factor into differentiated cells explanted from distinct tissues induces cells with functional and molecular attributes of their corresponding tissue-specific SCs that can be expanded ex vivo. The ySC state can be transmitted through cell generations without the need for continuous expression of ectopic YAP/TAZ, indicating that a transient activation of ectopic YAP or TAZ is sufficient to induce a heritable self-renewing state. Differently from induced pluripotent stem cells (iPSCs) or other reprogramming efforts, ySCs preserve a memory of the tissue of origin, expanding the current reprogramming paradigms by focusing on somatic stem cell generation from related cells of the same lineage.

Several lines of evidence support the notion that ySCs originate from conversion of differentiated cells rather than from amplification of rare pre-existing endogenous SCs. In particular, we used lineage tracing strategies employing established Cre drivers to label differentiated cells and follow their fate after YAP-induction. We found that ySCs indeed retained the genetic label specific of the original differentiated cells. Conversely, colonies and organoids emerging from native SCs were invariably unlabeled by the same genetic tracers. In the case of the mammary gland, we also used FACS to sort terminally differentiated cells from luminal progenitors and show that YAP can effectively operate on LD cells. Beyond lineage tracing, ySCs were induced with relatively high frequency after YAP expression, whereas no outgrowths emerged from differentiated cells expressing control vectors or transcriptionally inactive YAP, a scenario that argues against ySCs emergence from rare, pre-existing SCs. We also entertained the possibility that YAP overexpression may selectively expand rare native stem/progenitor cells. We consider this possibility also unlikely because YAP expression has no effect on the colony-forming capacity and expandability of native SCs. The latter finding is perhaps consistent with the fact that native SCs cultured ex vivo already contain transcriptionally active endogenous YAP/TAZ.

It is worth noting that, in mammary gland and pancreatic acinar cells, we were able to obtain transition to the corresponding tissue SCs starting from mature, adult differentiated cells, highlighting how YAP can imbue these lineages with a remarkable plasticity. However, we carried out our reprogramming experiments on fetal neurons because primary adult neurons cannot be effectively cultured ex vivo. Although they are post-mitotic, these early neurons may be particularly competent for YAP-induced reprogramming. Future work and technological advancements will be required to determine whether adult neurons can be reprogrammed by YAP similarly to fetal neurons.

Our procedure generates cells with normal SC traits as suggested by several lines of evidence: ySCs can be expanded over multiple passages as self-expanding organoids or neurospheres; ySCs readily generate a multilineage progeny reminiscent of the corresponding native SCs (for example, in the case of yMaSCs, reprogrammed SCs generate minigland-like organoids in vitro that, when transplanted in vivo, regenerate a normal ductal tree in the cleared fat pad); ySCs are not transformed and non-tumorigenic; and, at the transcriptional level, ySCs display remarkable overlaps with their native counterparts.

Lineage plasticity and reversion to an SC-like status rarely occur in normal tissues but are associated with tissue repair or oncogenic activation (Blanpain and Fuchs, 2014). Of note, genetic depletion of YAP and/or TAZ in several adult epithelia is inconsequential for normal homeostasis but, in fact, essential for regeneration, tumor growth (Zanconato et al., 2016), and, as shown here, for expansion of somatic SCs in vitro. It is thus tempting to propose that the path for lineage-specific reprogramming outlined here may be activated whenever natural, pathological, or ex vivo conditions demand generation and expansion of new SCs using differentiated cells as facultative SCs without losing tissue memory. Further work is required to validate that YAP reprogramming can occur in vivo and particularly in humans. If it does, it may be worth exploring means to exploit this path to facilitate tissue repair and regeneration in distinct tissues. From this perspective, the inability to cross lineage boundaries might represent a potential limitation for in vitro applications because some differentiated cells may not be readily accessible. However, if the same strategy could be applied directly in vivo, then the ability of a single factor to generate proliferative and multipotent SCs while retaining tissue memory would provide an advantage over other reprogramming and transdifferentiation strategies that employ either complex cocktails of transcription factors or the passing through an embryonic-like pluripotent state (Bar-Nur et al., 2015, Xu et al., 2015).

In conclusion, finding that YAP/TAZ, as a single factor, can reprogram distinct cell types into their corresponding tissue-specific SCs may have implications for regenerative medicine, for discovering still unknown determinants of somatic stemness, and, more broadly, for ad hoc expansion of somatic cells.

## Experimental Procedures

### Primary Mammary Epithelial Cell Isolation and Induction of yMaSCs

Primary mammary epithelial cells (MECs) were isolated from the mammary glands of 8- to 12-week-old virgin C57BL/6J mice (unless otherwise specified) according to standard procedures (Stingl et al., 2006; see Supplemental Experimental Procedures for details). Animal experiments were performed adhering to our institutional guidelines, and approved by OPBA and the Ministry of Health. To separate various MEC subpopulations, cells were stained for 30 min at 4°C with antibodies against CD49f (phycoerythrin [PE]-Cy5, catalog no. 551129, BD Biosciences), CD29 (PE-Cy7, catalog no. 102222, BioLegend), CD61 (PE, catalog no. 553347, BD Biosciences), EpCAM (fluorescein isothiocyanate [FITC], catalog no. 118208, BioLegend), and lineage markers (allophycocyanin [APC] mouse lineage antibody cocktail, catalog no. 51-9003632, BD Biosciences) in DMEM/F12.

The stained cells were then resuspended in PBS/BSA 0.1% and sorted on a BD FACS Aria sorter (BD Biosciences) into LD cells, LP cells, and MaSCs.

Primary sorted subpopulations from FACS were plated on collagen I-coated supports and cultured in two dimensions in mammary gland (MG) medium (DMEM/F12 supplemented with glutamine, antibiotics, 10 ng/ml murine epidermal growth factor [EGF], 10 ng/ml murine basic Fibroblast growth factor (bFGF), and 4 μg/ml heparin with 2% fetal bovine serum [FBS]).

For induction of yMaSCs, LD cells were transduced for 48 hr with FUW-tetO-YAP or FUW-tetO-TAZ in combination with rtTA-encoding lentiviruses. As a (negative) control, LD cells were transduced with either FUW-tetO-EGFP (Figures 1E, 1F and S1F) in combination with rtTA-encoding lentiviruses. After infection, adherent cells were washed and treated with 2 μg/ml doxycycline for 7 days in MG medium for activating tetracycline-inducible gene expression (see scheme in Figure 1D) to obtain yMaSCs. After doxycycline treatment for 7 days in two-dimensional culture, yMaSCs were processed for further assays or analysis. Unless otherwise specified, yMaSCs were generated from wild-type YAP (FUW-tetO-WTYAP). For the experiment depicted in Figures 2B, 2C and S2A, we first FACS-purified LD cells and MaSC-enriched populations (using CD61 and CD49f as described previously for Figure 1A) from *K8-CreERT2; R26-LSL-YFP/+* or *K14-CreERT2; R26-LSL-YFP/+* virgin female mice. These cells were plated, and, after attachment, they were treated with 1 μM 4-hydroxy (4OH)-tamoxifen for 24 hr. Cells were then transduced for 48 hr with FUW-tetO-WTYAP in combination with stable rtTA-encoding lentiviral supernatant. Negative control cells were provided by LD cells transduced with FUW-tetO-MCS (empty vector) in combination with rtTA-encoding lentiviral supernatants. After infection, cells were washed and treated with doxycycline in MG medium as above.

### Primary Neuron Isolation and Induction of yNSCs

Neurons were prepared from hippocampi or cortices of late (E18–19) embryos or post-natal day 1 (P1) pups as described previously (Han et al., 2008). Briefly, hippocampi and cortices were dissected under the microscope in ice-cold Hank’s balanced salt solution (HBSS) as quickly as possible, incubated with 0.05% trypsin (Life Technologies) for 15 min at 37°C, and, after trypsin blocking, resuspended in DMEM/10% FBS supplemented with 0.1 mg/ml DNase I (Roche) and mechanically dissociated by extensive pipetting. Cells were then plated on poly-L-lysine-coated wells in DMEM supplemented with 10% FBS, glutamine, and antibiotics for hippocampal neurons or in DMEM/Neurobasal (1:1) supplemented with 5% FBS, 1× B27, glutamine, and antibiotics for cortical neurons (day 1). After 24 hr (day 2), the medium of both hippocampal and cortical preparations was changed to fresh DMEM/Neurobasal (1:1) supplemented with 5% FBS, 1× B27, glutamine, and antibiotics. For reprogramming experiments, neurons were infected on the following day (day 3) with FUW-tetO-WTYAP and FUdeltaGW-rtTA viral supernatants. Negative controls were provided by neurons transduced with FUdeltaGW-rtTA alone or in combination with FUW-tetO-EGFP or FUW-tetO-MCS (empty vector). After 24 hr (day 4), the medium was changed, and cells were incubated in Neurobasal medium supplemented with 1× B27, glutamine, antibiotics, and 5 μM AraC (Sigma) for an additional 7 days, at the end of which well differentiated, complex network-forming neurons were visible. To induce yNSCs formation, treated neurons were switched to NSC medium (DMEM/F12 supplemented with 1× N2, 20 ng/ml murine EGF, 20 ng/ml murine bFGF, glutamine, and antibiotics) and doxycycline for activating tetracycline-inducible gene expression. After 7 days, fresh doxycycline was added. Sphere formation was evident upon YAP induction after 14 days of doxycycline treatment. Spheres (P0 spheres) were mechanically detached from the plates by tapping and flushing (not by trypsinizing), transferred into a 15-ml plastic tube, and left to sediment (usually 5 min). After discarding the supernatant, spheres were dissociated with 1-–2 ml of TrypLE Express (Life Technologies) and mechanical pipetting. TrypLE Express was then diluted 1:5 in NSC medium, and cells were centrifuged and resuspended in NSC medium without doxycycline. For the successive passages, spheres were harvested and dissociated, and yNSCs were routinely cultured and passaged without doxycycline in NSC medium as for normal NSCs.

### Pancreatic Acinar Cell Isolation and Induction of yDucts

Primary pancreatic acini were isolated from the pancreata of 6- to 9-week-old mice according to standard procedures (Means et al., 2005). Digested tissue was filtered through a 100-μm nylon cell strainer. The quality of isolated acinar tissue was checked under the microscope. For culture of entire acini, explants were seeded in neutralized rat tail collagen type I (Cultrex)/acinar culture medium (1:1) (Means et al., 2005), overlaid with acinar culture medium (Waymouth’s medium [Life Technologies] supplemented with 0.1% FBS [Life Technologies], 0.1% BSA, 0.2 mg/ml soybean trypsin inhibitor [SBTI], 1× insulin-transferrin-selenium-ethanolamine [ITS-X] [Life Technologies], 50 μg/ml bovine pituitary extract [BPE] [Life Technologies], 1μg/ml dexamethasone [Sigma], and antibiotics) when collagen formed a gel. For culture of isolated acinar cells, acini were further digested in 0.05% trypsin for 30 min at 37°C to obtain a single-cell suspension. Single acinar cells were plated in 100% Matrigel. When Matrigel formed a gel, cells were supplemented with pancreatic organoid medium (advanced DMEM/F12 supplemented with 1× B27, 1.25 mM N-acetylcysteine, 10 nM gastrin, 50 ng/ml murine EGF, 100 ng/ml human Noggin, 100 ng/ml human FGF10, 10 mM nicotinamide, 1 μg/ml R-Spondin1, and antibiotics) supplemented with 0.2 mg/ml SBTI. For induction of pancreatic organoids, entire acini or single acinar cells of the indicated genotypes cells were seeded in medium supplemented with 2 μg/ml doxycycline. Negative control cells were cultured under the same conditions in the absence of doxycycline. Cells were treated with 2 μg/ml doxycycline for 7 days, and organoid formation was morphologically followed.

## Author Contributions

T.P. carried out the experiments on neurons and exocrine pancreas; A.F., on neurons; and L.A., on the mammary gland cells. C.F. and G.B. performed the sorting experiments. S. Bresolin, G.B., and S. Bicciato performed microarrays and biostatistics. D.D.B. performed molecular biology and Ifs. A.R. performed mouse surgery and helped with transplantation. S.P. conceived the initial hypothesis and experimental design. M.C. and S.P. planned, discussed, and organized the work. S.P., M.C., L.A., T.P., and A.F. wrote the manuscript.

## Accession Numbers

The accession number for the Affymetrix data reported in this paper is GEO: GSE70174.

## Supplemental Information

Document S1. Supplemental Experimental Procedures and Figures S1–S6 Document S1. Supplemental Experimental Procedures and Figures S1–S6

Movie S1. Time-Lapse Movie Showing Conversion of Acini into yDucts, Related to Figure 6 The movie shows the same field of whole pancreatic acini derived from *R26-rtTA; tetO-YAP*^S127A^ mice during their conversion into cyst-like organoids at the indicated time points (hours) after doxycycline addition. Movie S1. Time-Lapse Movie Showing Conversion of Acini into yDucts, Related to Figure 6

Movie S2. Time-Lapse Movie showing Conversion of a Single Acinus into a yDuct, Related to Figure 6 The movie shows a single pancreatic acinus derived from *R26-rtTA; tetO-YAP*^S127A^ mice during conversion into cyst-like organoid at the indicated time points (hours) after doxycycline addition. Movie S2. Time-Lapse Movie showing Conversion of a Single Acinus into a yDuct, Related to Figure 6

Movie S3. Time-Lapse Movie Showing Conversion of a Lineage-Traced Single Acinus into a yDuct, Related to Figure 6 Bright-field and GFP-fluorescence movie showing a single pancreatic acinus derived from *Ptf1a-CreERTM; R26-LSL-rtTA-IRES-EGFP; tetO-YAP*^S127A^ mice during conversion into cyst-like organoid at the indicated time points (hours) after doxycycline addition. Movie S3. Time-Lapse Movie Showing Conversion of a Lineage-Traced Single Acinus into a yDuct, Related to Figure 6

Document S2. Article plus Supplemental Information Document S2. Article plus Supplemental Information
