# Supplementary material for: Induction of Expandable Tissue-Specific Stem/Progenitor Cells through Transient Expression of YAP/TAZ
Source: Cell Stem Cell. 2016 Dec 1;19(6):725–37. doi: 10.1016/j.stem.2016.08.009 (PMC5145813; doi:10.1016/j.stem.2016.08.009)
Supplement: Document S1. Supplemental Experimental Procedures and Figures S1–S6 [file mmc1.pdf]

Cell Stem Cell, Volume *19*

Supplemental Information

Induction of Expandable Tissue-Specific Stem/Progenitor Cells through Transient Expression of YAP/TAZ

Tito Panciera, Luca Azzolin, Atsushi Fujimura, Daniele Di Biagio, Chiara Frasson, Silvia Bresolin, Sandra Soligo, Giuseppe Basso, Silvio Bicciato, Antonio Rosato, Michelangelo Cordenonsi, and Stefano Piccolo

THIS SECTION CONTAINS:

- SUPPLEMENTAL FIGURES 1-6 AND LEGENDS
- SUPPLEMENTAL MOVIE LEGENDS
- EXTENDED EXPERIMENTAL PROCEDURES
- SUPPLEMENTAL REFERENCES

SUPPLEMENTAL FIGURES AND LEGENDS

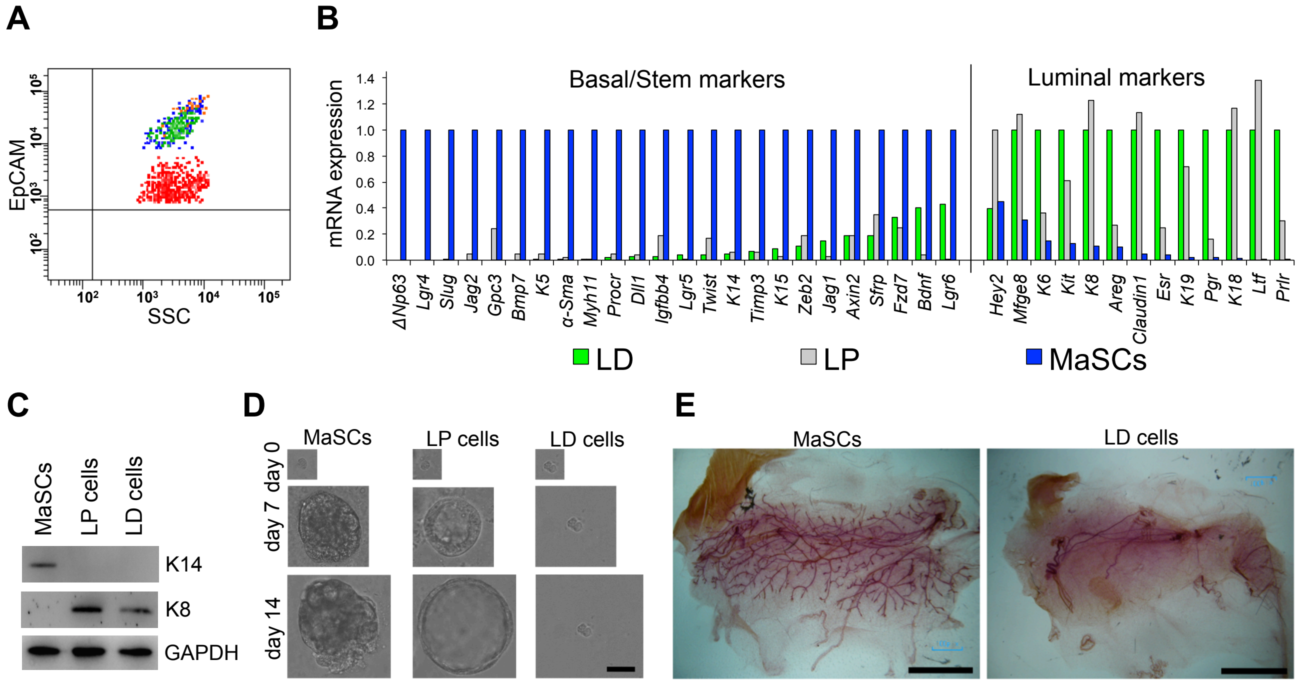

F

1 plated cell in 1 single well; visually verified	Experiment			Mean ± SD
	#1	#2	#3	
**LD: rtTA/YAP wt transduced**				
# of single LD cells	n = 71	n = 68	n = 57	
% of single cells that gave rise to a single LP-like colony	0%	0%	0%	0
% of single cells that gave rise to a single MaSC-like colony	19.7%	14.7%	21.1%	18.5 ± 3.4
**LD: rtTA/EGFP transduced** (negative control)				
# of single LD cells	n = 56	n = 64	n = 70	
% of single cells that gave rise to a single LP-like colony	0%	0%	0%	0
% of single cells that gave rise to a single MaSC-like colony	0%	0%	0%	0
**LD: rtTA/YAP S94A transduced** (transcriptionally inactive, negative control)				
# of single LD cells	n = 73	n = 46	n = 50	
% of single cells that gave rise to a single LP-like colony	0%	0%	0%	0
% of single cells that gave rise to a single MaSC-like colony	0%	0%	0%	0

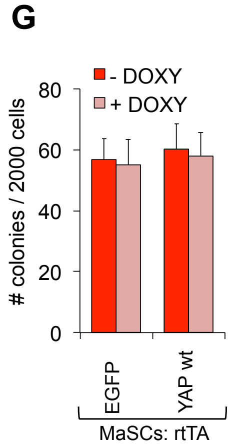

Figure S1

Figure S1. Characterization of FACS-sorted mammary cells and induction of MaSC-like traits in luminal differentiated cells by YAP/TAZ. Related to Figure 1.

A, FACS profile for EpCAM of the experiments represented in Figure 1A.

B-C, qRT-PCRs and western blots for the indicated basal/stem and luminal markers in MaSCs, LP, and LD cells obtained by FACS, as in Figure 1A. In **B**, data are normalized to *Gapdh* expression and are referred to MaSC levels for basal genes, to LP levels for *Hey1*, and to LD levels for all the other luminal markers (each set to 1). Results are representative of at least three independent experiments (each using mammary glands from n=20 mice) performed in triplicate. In **C**, GAPDH serves as loading control.

D, Representative images of mammary colonies formed by the indicated cells, growing at the indicated time points in mammary colony medium. MaSCs formed solid outgrowths, while LD remained as single cells. LP cells, despite being able to form acinar (cavitated) colonies, were unable to self-renew after passaging, or to form organoids when transferred in 100% Matrigel/mammary organoid medium culture system (not shown). Pictures are representative of three independent experiments performed with six technical replicates. Scale bar, 170 μm.

E, Representative images of whole mount hematoxylin staining of cleared fat pads injected with purified MaSCs (leading to outgrowth of a ductal mammary tree) or of LD cells as negative control. LP cells were similarly void of regenerative potential in vivo (not shown). Scale bars, 1 cm.

F, Detailed quantification of the conversion rates of single, visually inspected, LD cells, expressing the indicated constructs and plated in 96-well plates, into a MaSC-like state as determined by % of colony formation. EGFP- and YAP S94A-transduced cells serve as negative control. For quantification of the lentiviral infection and of the organoid-forming efficiency of yMaSCs colonies once transferred in organoid medium, see Methods.

G, Quantifications of mammary colonies formed by the indicated cells, 15 days after seeding in mammary colony medium. Data are presented as mean + s.d.

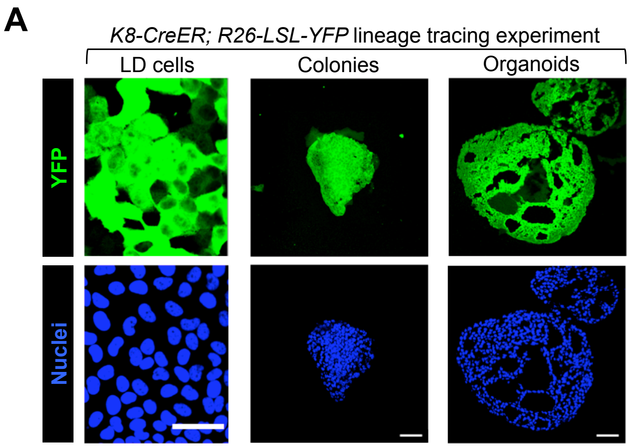
A
K8-CreER; R26-LSL-YFP lineage tracing experiment
LD cells
Colonies
Organoids
YFP
Nuclei

Figure S2

Figure S2. Lineage tracing of yMaSC-derived colonies and organoids.
Related to Figure 2.

A, anti-YFP immunostaining of the lineage tracing experiment showing that yMaSC-derived colonies and organoids originate from tamoxifen-treated *K8-CreERT2; R26-LSL-YFP* LD cells. Scale bars, 49 μm.

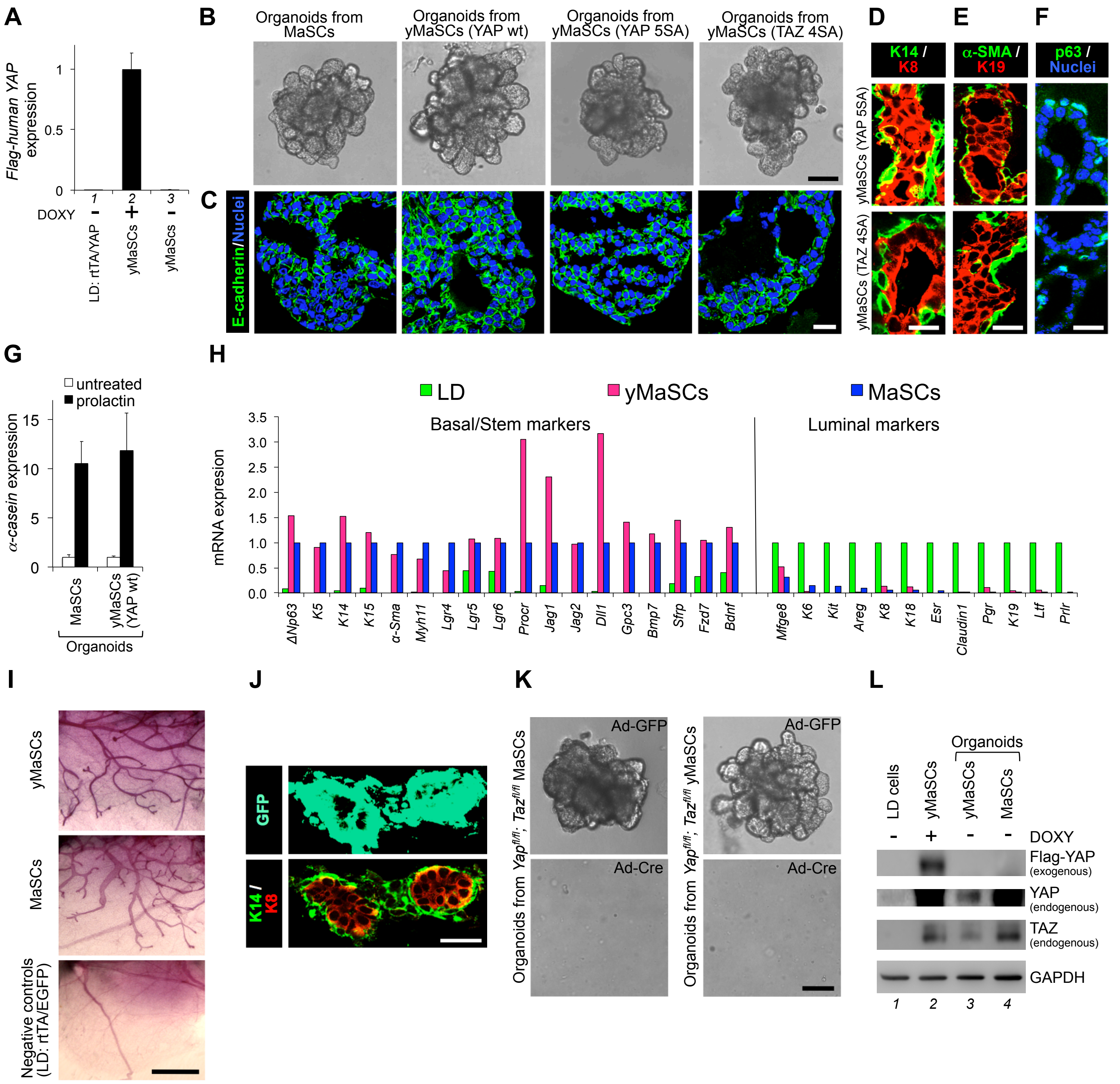

A
Flag-human YAP expression
DOXY
LD: rtTA/YAP
yMaSCs
yMaSCs
B
Organoids from MaSCs
Organoids from yMaSCs (YAP wt)
Organoids from yMaSCs (YAP 5SA)
Organoids from yMaSCs (TAZ 4SA)
C
E-cadherin/Nuclei
D
K14 / K8
E
α-SMA / K19
F
p63 / Nuclei
yMaSCs (YAP 5SA)
yMaSCs (TAZ 4SA)
G
untreated
prolactin
α-casein expression
MaSCs
yMaSCs (YAP wt)
Organoids
H
LD
yMaSCs
MaSCs
Basal/Stem markers
Luminal markers
mRNA expresion
ΔNp63
K5
K14
K15
α-Sma
Myh11
Lgr4
Lgr5
Lgr6
Procr
Jag1
Jag2
Dll1
Gpc3
Bmp7
Sfrp
Fzd7
Bdnf
Mfge8
K6
Kit
Areg
K8
K18
Esr
Claudin1
Pgr
K19
Ltf
Prlr
I
yMaSCs
MaSCs
Negative controls (LD: rtTA/EGFP)
J
GFP
K14 / K8
K
Ad-GFP
Ad-Cre
Organoids from Yap fl/fl; Taz fl/fl MaSCs
Organoids from Yap fl/fl; Taz fl/fl yMaSCs
L
Organoids
LD cells
yMaSCs
yMaSCs
MaSCs
DOXY
Flag-YAP (exogenous)
YAP (endogenous)
TAZ (endogenous)
GAPDH

Figure S3

Figure S3. Characterization of mammary organoids derived from MaSCs and yMaSCS. Related to Figure 3.

A, qRT-PCRs for transgenic Flag-human YAP in the indicated samples. Samples are: LD cells infected with rtTA and tetO-YAP lentiviral particles and either left untreated (lane 1, NO Doxy) or cultured in the presence of doxycycline to induce yMaSCs (lane 2). Next, yMaSCs were maintained in Matrigel as organoids (see Methods and Figure 1D) in the absence of doxycycline for 5 months (lane 3, NO Doxy). Data are normalized to *Gapdh* expression and are presented as mean + s.d. of two independent replicates. Note that yMaSCs kept in absence of doxycycline do not express the transgene.

B, Representative images of MaSCs or yMaSCs organoids (derived from YAPwt, YAP5SA or TAZ4SA, as indicated); see Figure 1D for a diagram of the experimental plan. Scale bar, 250 μm.

C, Organoids from MaSCs (positive control) and the indicated yMaSCs expressed E-cadherin by confocal immunofluorescence on frozen sections. Scale bar, 18 μm.

D-F, Organoids from the indicated yMaSCs expressed basal/stem (K14, α-SMA, p63) and luminal (K8, K19) markers by confocal immunofluorescence on frozen sections. Scale bars, 17 μm.

G, Compendium of Figure 3F. Treatment with prolactin triggers *α-casein* expression in MaSC- and yMaSC-derived organoids, as monitored by qRT-PCR. Data are normalized to *Gapdh* expression. Untreated samples were set to 1. Results are representative of two independent experiments, each performed in triplicate. Data are mean + s.d.

H, Comparison by qRT-PCR of FACS-purified MaSC-enriched basal cells (MaSCs) from the mammary gland and FACS-purified yMaSCs from yMaSC-derived organoids. Purified LD cells were used as control. Data are normalized to *Gapdh* expression and are referred to MaSCs levels for basal genes and to LD levels for all the luminal markers (each set to 1).

I, Representative images of whole-mount hematoxylin staining of cleared fat pad with reconstituted mammary trees from transplanted yMaSCs (from wtYAP), native MaSCs (positive control) and rtTA/EGFP control LD cells (negative control). Scale bar, 0.5 cm.

J, This refers to Figure 3J of the Main Text. Representative sections of virgin mammary gland tree derived from injected MaSCs stained for GFP, K14 and K8. Scale bar, 21 μm.

K, Organoids were obtained from *Yap*$^{fl/fl}$*; Taz*$^{fl/fl}$ MaSCs and yMaSCs. During passaging at the single cell level, they were transduced with Ad-Cre or Ad-GFP as control. Panels are representative images of the resulting outgrowths. No organoid ever formed in absence of YAP/TAZ. Scale bar, 250 μm.

L, Panels are western blots for YAP and TAZ of lysates from the indicated cells. Lane 1: FACS-sorted LD cells. Lane 2: yMaSCs (wtYAP) after seven days of doxycycline treatment (as in Figure 1D); tagged Flag-hYAP (with a higher molecular weight than endogenous mouse YAP) is induced. Lane 3: organoids from yMaSCs cultured in the absence of doxycycline (Flag-hYAP turned off, but endogenous YAP/TAZ remain expressed). Lane 4: control of endogenous MaSCs. GAPDH serves as loading control.

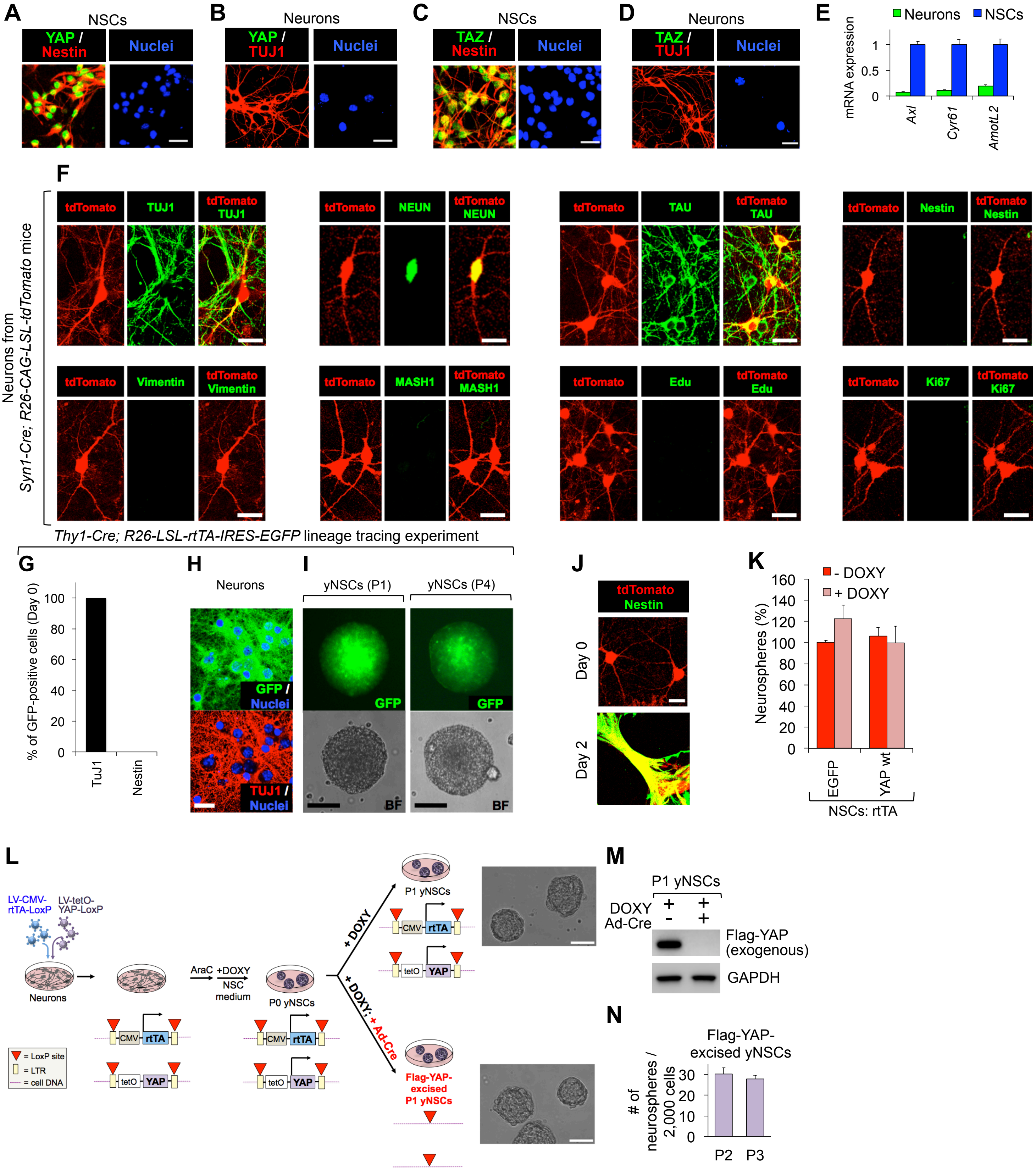
A
NSCs
YAP / Nestin
Nuclei
B
Neurons
YAP / TUJ1
Nuclei
C
NSCs
TAZ / Nestin
Nuclei
D
Neurons
TAZ / TUJ1
Nuclei
E
Neurons
NSCs
mRNA expression
Axl
Cyr61
AmotL2
F
Neurons from Syn1-Cre; R26-CAG-LSL-tdTomato mice
tdTomato
TUJ1
NEUN
TAU
Nestin
Vimentin
MASH1
Edu
Ki67
Thy1-Cre; R26-LSL-rtTA-IRES-EGFP lineage tracing experiment
G
% of GFP-positive cells (Day 0)
TuJ1
Nestin
H
Neurons
GFP / Nuclei
TUJ1 / Nuclei
I
yNSCs (P1)
yNSCs (P4)
GFP
BF
J
tdTomato Nestin
Day 0
Day 2
K
- DOXY
+ DOXY
Neurospheres (%)
EGFP
YAP wt
NSCs: rtTA
L
LV-CMV-rtTA-LoxP
LV-tetO-YAP-LoxP
Neurons
AraC +DOXY NSC medium
P0 yNSCs
+ DOXY
P1 yNSCs
+ DOXY; + Ad-Cre
Flag-YAP-excised P1 yNSCs
CMV
rtTA
tetO
YAP
= LoxP site
= LTR
= cell DNA
M
P1 yNSCs
DOXY
Ad-Cre
Flag-YAP (exogenous)
GAPDH
N
Flag-YAP-excised yNSCs
# of neurospheres / 2,000 cells
P2
P3

Figure S4

Figure S4. Expression of YAP/TAZ in neurons and lineage tracing experiments showing that yNSCs originate from neurons. Related to Figure 4.

A-B, Representative confocal images of NSCs (plated as monolayer) and hippocampal neurons, costained for YAP/Nestin and YAP/TUJ1, respectively. Nuclei were stained with DAPI. Scale bars, 23 μm.

C-D, Representative confocal images of endogenous TAZ costained with Nestin in primary NSCs (**C**) or with TUJ1 in primary hippocampal neurons (**D**). Nuclei were stained with DAPI. Scale bars, 23 μm.

E, qRT-PCRs for the known YAP/TAZ targets genes *Axl, Cyr61 and AmotL2* in hippocampal neurons and NSCs (mean + s.d.). Results are representative of three independent experiments performed in triplicate. Data were normalized to *Gapdh* expression.

F, tdTomato fluorescence and immunostaining for the indicated markers in cortical neurons obtained from *Syn1-Cre; R26-CAG-LSL-tdTomato* mice. Scale bars, 19 μm.

G-I, Lineage tracing experiment with the *Thy1-Cre* driver showing that yNSCs originate from neurons. **G**, quantification of the percentage of EGFP-positive cells (Thy1-traced) expressing TUJ1 or Nestin (n=200). **H**, immunostaining for GFP and TUJ1 in hippocampal neurons obtained from *Thy1-Cre; R26-LSL-rtTA-IRES-EGFP* mice. **I**, bright field and GFP-fluorescence pictures of yNSC-generated neurospheres obtained from neurons as in (G, H) after reprogramming using doxycycline-inducible YAP. Scale bars in **H**, 37 μm, in **I**, 105 μm.

J, Immunofluorescences for Nestin during the conversion of Syn1-Cre/tdTomato-traced YAP-expressing cortical neurons (day 0) to yNSCs (day 2).

K, Quantifications of neurospheres formed by the indicated cells, 7 days after seeding suspension cultures in NSC medium. Data are presented as mean + s.d.

L-N, Hippocampal neurons were transduced with the Cre-excisable vectors encoding for rtTA and doxycycline-inducible Flag-human wild-type YAP, and treated to obtain P0 yNSCs. P0 yNSCs were dissociated at the single cell level and replated in NSC medium + doxycycline to allow P1 yNSCs formation with or without Ad-Cre, that enables the excision of the whole viral integrated cassette. **L,** the panel includes representative images of the yNSCs, before and post-excision. Scale bar, 210 μm. **M**, Flag-human YAP could not be detected post-excision. GAPDH serves as loading control. **N**, quantification of

neurospheres from yNSCs post-excision in two serial passages. Results are representative of two independent experiments, each performed in six replicates. Data are mean + s.d.

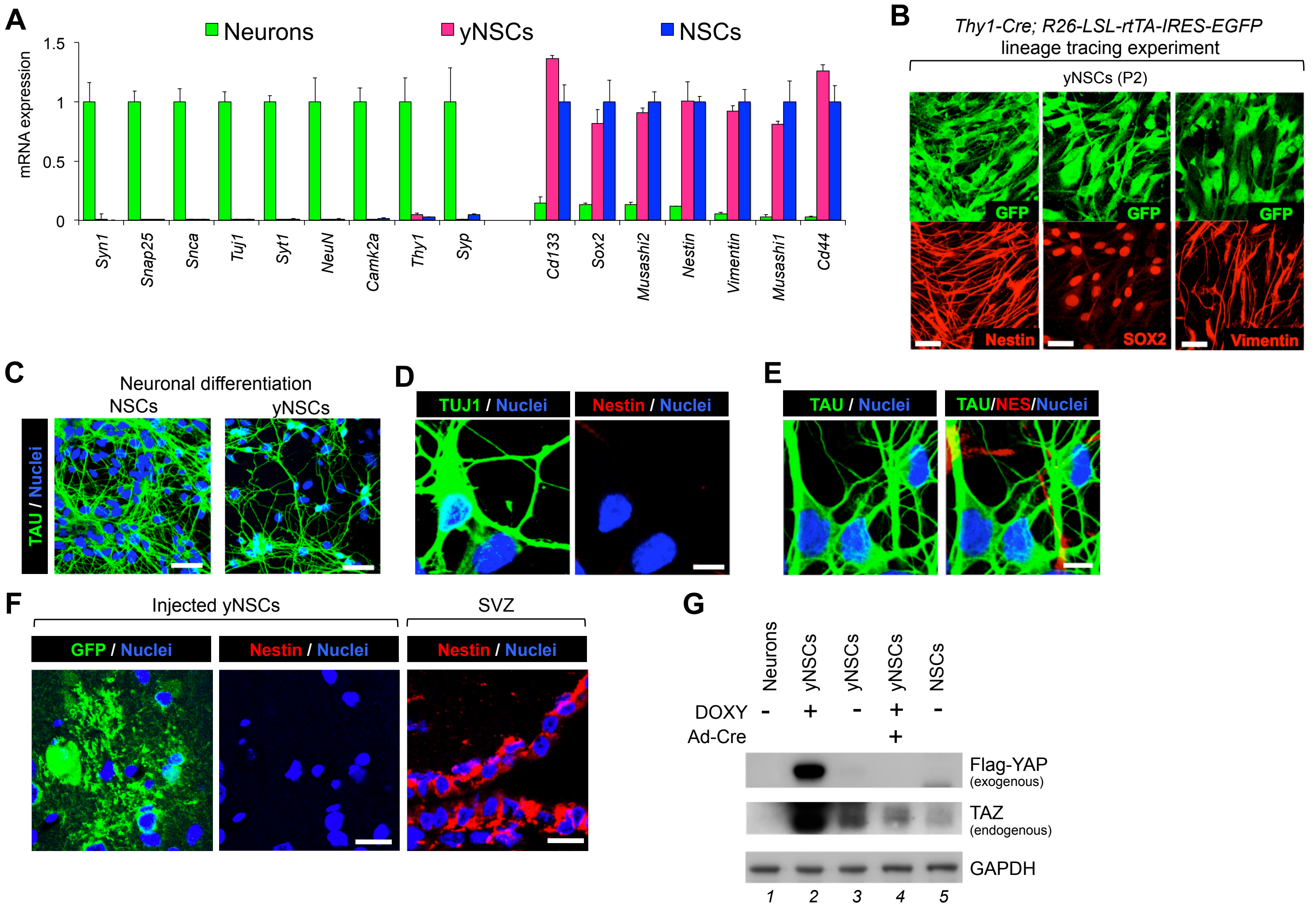
A
Neurons
yNSCs
NSCs
mRNA expression
1.5
1
0.5
0
Syn1
Snap25
Snca
Tuj1
Syt1
NeuN
Camk2a
Thy1
Syp
Cd133
Sox2
Musashi2
Nestin
Vimentin
Musashi1
Cd44
B
Thy1-Cre; R26-LSL-rtTA-IRES-EGFP
lineage tracing experiment
yNSCs (P2)
GFP
Nestin
SOX2
Vimentin
C
Neuronal differentiation
NSCs
yNSCs
TAU / Nuclei
D
TUJ1 / Nuclei
Nestin / Nuclei
E
TAU / Nuclei
TAU/NES/Nuclei
F
Injected yNSCs
SVZ
GFP / Nuclei
Nestin / Nuclei
G
Neurons
yNSCs
NSCs
DOXY
Ad-Cre
Flag-YAP
(exogenous)
TAZ
(endogenous)
GAPDH
1
2
3
4
5

Figure S5

Figure S5. Molecular characterization of yNSCs. Related to Figure 5.

A, qRT-PCRs for the indicated neuronal and neural stem cell markers in cortical neurons, yNSCs and NSCs. Data are normalized to *Gapdh* expression and are referred to neurons for neuronal genes, and to NSCs for neural stem cell markers (each set to 1). Results are representative of at least three independent experiments performed in triplicate.

B, Immunostainings of yNSCs derived as in Figure S4G-I plated in 2D, showing positivity for GFP and neural stem cell markers Nestin, SOX2 and Vimentin. Scale bars, 37μm.

C, This refers to Figure 5E. yNSCs and NSCs were plated and differentiated toward a neuronal fate (see Methods). Panels represent confocal images for neuronal differentiation marker TAU. Scale bars, 50 μm.

D-E, These refer to Figure 5E of the main text and to Figure S5C. By immunofluorescence, neurons differentiated from yNSCs are TUJ1-positive (D) or TAU-positive (E) but are negative for Nestin. Scale bars, 9μm.

F, Panels are representative confocal images showing that injected cells (GFP-positive) lost expression of the NSC marker Nestin. A field of the subventricular zone (SVZ) of the same brain sections is shown as positive control of the anti-Nestin antibody staining.

G, Panels are western blots for YAP and TAZ from protein extracts of the indicated cells. Lane 1: neurons. Lane 2: yNSCs (P0) were obtained using excisable YAP transgene, and maintained in Doxy; as in Figure S4M-N. Cells (from P2-to-P3) were plated as monolayer in presence of Doxy and lysed after 1 day. Lane 3: the same yNSCs of lane 2, kept in absence of Doxy from P2. Lane 4: yNSCs as in lane 2, but after excision of the viral cassette (at P1, as Figure S4L-M). Lane 5: lysates of NSCs as comparison.

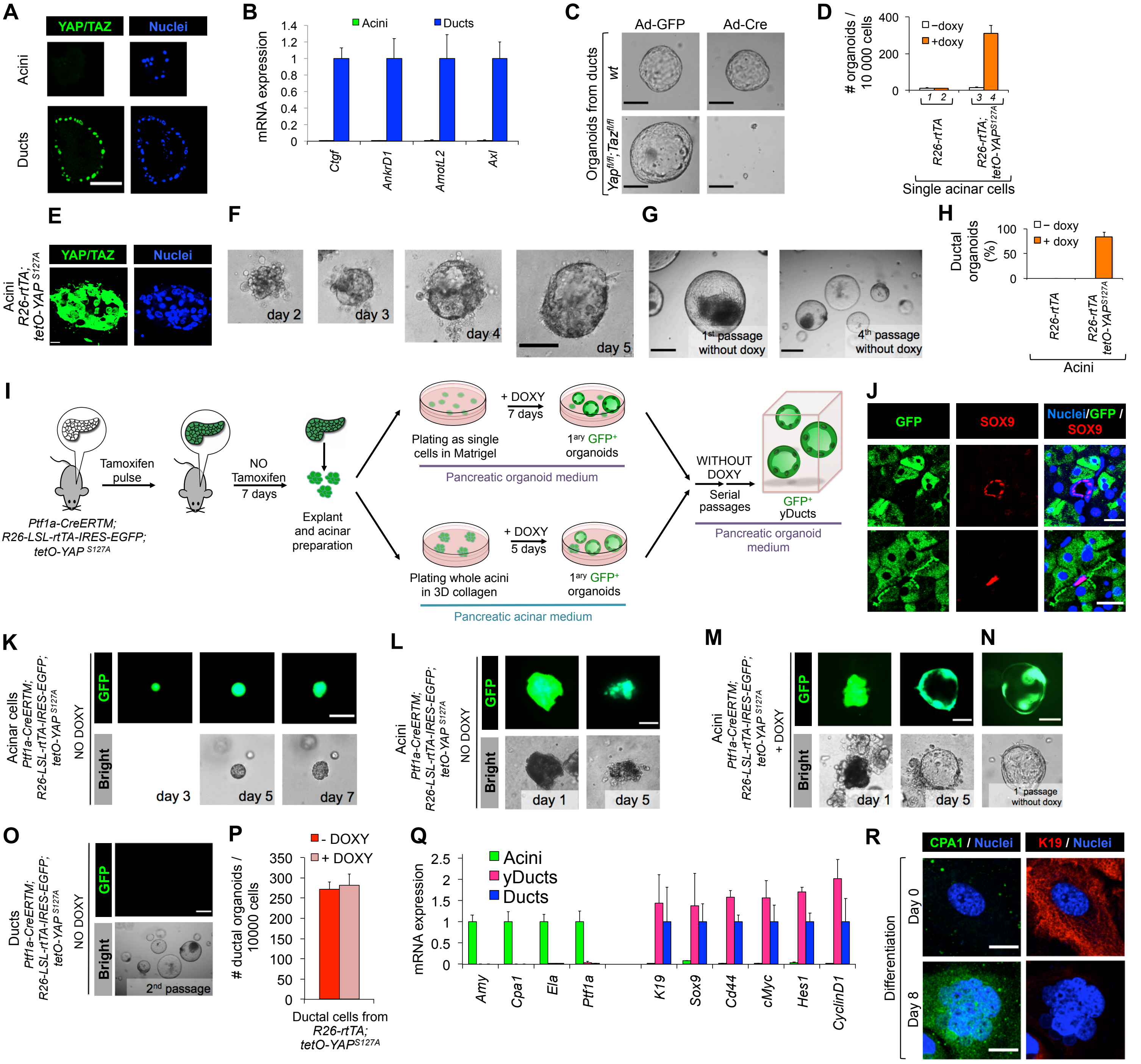
A
YAP/TAZ
Nuclei
Acini
Ducts
B
Acini
Ducts
mRNA expression
Ctgf
AnkrD1
AmotL2
Axl
C
Ad-GFP
Ad-Cre
Organoids from ducts
wt
D
organoids / 10 000 cells
−doxy
+doxy
R26-rtTA
Single acinar cells
E
Acini
F
day 2
day 3
day 4
day 5
G
1st passage without doxy
4th passage without doxy
H
Ductal organoids (%)
− doxy
+ doxy
R26-rtTA
Acini
I
Tamoxifen pulse
NO Tamoxifen 7 days
Explant and acinar preparation
Plating as single cells in Matrigel
+ DOXY 7 days
GFP+ organoids
Pancreatic organoid medium
Plating whole acini in 3D collagen
+ DOXY 5 days
GFP+ organoids
Pancreatic acinar medium
WITHOUT DOXY
Serial passages
GFP+ yDucts
Pancreatic organoid medium
J
GFP
SOX9
Nuclei/GFP / SOX9
K
Acinar cells
NO DOXY
GFP
Bright
day 3
day 5
day 7
L
Acini
NO DOXY
day 1
day 5
M
Acini
+ DOXY
day 1
day 5
N
1st passage without doxy
O
Ducts
NO DOXY
2nd passage
P
- DOXY
+ DOXY
# ductal organoids / 10000 cells
Ductal cells from R26-rtTA; tetO-YAPS127A
Q
Acini
yDucts
Ducts
mRNA expression
Amy
Cpa1
Ela
Ptf1a
K19
Sox9
Cd44
cMyc
Hes1
CyclinD1
R
CPA1 / Nuclei
K19 / Nuclei
Differentiation
Day 0
Day 8

Figure S6

Figure S6. YAP expression converts pancreatic acinar explants to duct-like organoids.

Related to Figure 6.

A, Pancreatic ductal organoids ("Ducts", bottom panels) display nuclear YAP/TAZ by immunofluorescence. YAP/TAZ are not detected in pancreatic acini (top panels). Scale bar, 80μm.

B, qRT-PCRs for the known YAP/TAZ targets genes *Axl, Ctgf, Amotl2 and AnkrD1* in primary pancreatic acini and pancreatic ductal organoids (mean + s.d). Results are representative of three independent experiments performed in triplicate. Data were normalized to *18S rRNA* expression.

C, Ducts were derived from wild-type (*wt*) or $Yap^{fl/fl}$; $Taz^{fl/fl}$ mice and, during passaging at the single cell level, transduced with Ad-Cre or Ad-GFP as control. Panels are representative images of resulting outgrowths. Scale bars, 70 μm.

D, This refers to Figure 6C of the main text. Quantification of primary organoids arising from *R26-rtTA; tetO-*YAP^{S127A} single acinar cells. Lanes 1-3 are negative controls; although rare colonies were still counted in these control wells, at difference of bona fide organoids, in no case these could be amplified in subsequent passages. >90% of the YAP-induced yDucts in lane 4 were large and well-cavitated cysts, as those shown in Figure 6C, that were never found in controls. Data are presented as mean + s.d. and are representative of five independent experiments, performed with four technical replicates.

E, Representative immunostaining for YAP/TAZ showing control of the induction of transgenic YAP in pancreatic acini derived from *R26-rtTA; tetO-*YAP^{S127A} 5 days after Doxycycline addition. Scale bar, 24 μm.

F-G, Serial images of a whole acinus derived from *R26-rtTA; tetO-*YAP^{S127A} growing as cyst-like organoid at the indicated time points after Doxycycline addition (**F**) and after one or four passages in fresh Matrigel in the absence of Doxycycline (**G**). Scale bars, 70 μm in **F**; 290 μm in **G**. See also Movies S1 and S2.

H, Quantification of the ability of whole acini to form ductal organoids upon transgenic YAP overexpression as in Figure S6F. Data are presented as mean + s.d. and are representative of five independent experiments, performed with four technical replicates.

I, Schematic representation of the experiments performed with pancreatic acinar explants for lineage tracing. Pancreatic acini were isolated from *Ptf1a-CreERTM; R26-LSL-rtTA-IRES-EGFP; tetO-*YAP^{S127A} mice and either seeded as single cells in Matrigel or as whole acini in collagen (see Methods). Acinar cells were cultured in the presence of doxycycline

until primary organoids appeared. Organoids obtained from both culture conditions were then passaged in fresh Matrigel in the absence of doxycycline every 10 days.

J, Representative immunofluorescence pictures, showing that, 1 week after tamoxifen induction, *Ptf1a-CreERTM; R26-LSL-rtTA-IRES-EGFP* lineage tracer is labeling acinar cells, but not SOX9-positive ductal (top panels) or SOX9-positive centroacinar (bottom panels) cells. Scale bars, 19 μm.

K, This is a negative control that refers to Figure 6E. Panels are bright field and GFP-fluorescence pictures of transgenic *Ptf1a-CreERTM; R26-LSL-rtTA-IRES-EGFP; tetO-YAP*^{S127A} exocrine cells, at the indicated time points in absence of Doxy treatment. Scale bar, 33μm.

L, Negative control of the experiment shown in Figure S6M,N. Panels are bright field and GFP-fluorescence pictures of *Ptf1a-CreERTM; R26-LSL-rtTA-IRES-EGFP; tetO-YAP*^{S127A} whole exocrine acini, at the indicated time points in absence of Doxy treatment (Negative controls). Scale bar, 33μm

M-N, Lineage-tracing experiments using the *Ptf1a-CreERTM* driver. Panels are bright field and GFP-fluorescence pictures of transgenic YAP-expressing whole exocrine acini derived from *Ptf1a-CreERTM; R26-LSL-rtTA-IRES-EGFP; tetO-YAP*^{S127A} mice, at the indicated time points of Doxy treatment (**M**) and after passaging in absence of Doxy in fresh Matrigel (**N**). Scale bars, 33 μm in **M**; 70 μm in **N.** See also Movie S3.

O, Panels are bright field and GFP-fluorescence pictures of *Ptf1a-CreERTM; R26-LSL-rtTA-IRES-EGFP; tetO-YAP*^{S127A} ductal progenitors, at the indicated passage of organoid culture. Scale bar, 290 μm.

P, Quantifications of ductal organoids formed by the indicated cells 7 days after seeding in pancreatic organoid medium. Data are presented as mean + s.d.

Q, qRT-PCRs for the indicated exocrine and Ductal/progenitor markers in fresh pancreatic acini, yDucts and Ducts. Data are normalized to *18S* rRNA expression and are referred to Acini for exocrine differentiation markers, and to Ducts for Ductal/progenitor genes (each set to 1). Results are representative of four independent experiments performed in triplicate. Data are presented as mean + s.d.

R, Representative immunofluorescences for the ductal marker K19 and the exocrine marker CPA1 before (day 0) and after yDuct differentiation (day 8). Similar results were obtained with organoids from normal ducts (not shown). Scale bars:19 μm (day 0), 16 μm (day8).

SUPPLEMENTAL MOVIE LEGENDS

Movie S1. Time-lapse movie showing conversion of acini into yDucts. Related to Figure 6.

The movie shows the same field of whole pancreatic acini derived from *R26-rtTA; tetO-YAP*^{S127A} mice during their conversion into cyst-like organoids at the indicated time points (hours) after doxycycline addition.

Movie S2. Time-lapse movie showing conversion of a single acinus into yDuct. Related to Figure 6.

The movie shows a single pancreatic acinus derived from *R26-rtTA; tetO-YAP*^{S127A} mice during conversion into cyst-like organoid at the indicated time points (hours) after doxycycline addition.

Movie S3. Time-lapse movie showing conversion of a lineage traced- single acinus into yDuct. Related to Figure 6.

Bright-field and GFP-fluorescence movie showing a single pancreatic acinus derived from *Ptf1a-CreERTM; R26-LSL-rtTA-IRES-EGFP; tetO-YAP*^{S127A} mice during conversion into cyst-like organoid at the indicated time points (hours) after doxycycline addition.

EXTENDED EXPERIMENTAL PROCEDURES

Reagents, plasmids and transfections

Doxycycline hyclate, fibronectin, collagen I, heparin, insulin, dexamethasone, SBTI (Soybean Trypsin Inhibitor), gastrin, N-acethylcysteine, nicotinamide, T3 (Triiodo-L-Thyronine), tamoxifen and 4-OH-tamoxifen were from Sigma. Murine EGF, murine bFGF, human FGF10, human Noggin, human IGF, murine prolactin and BMP4 were from Peprotech. N2, B27 (always containing vitamin A), BPE and ITS-X (Insulin-Transferrin-Selenium-Ethanolamine) supplements were from Life Technologies. R-Spondin1 was from Sino Biological. Matrigel was from BD Biosciences (Corning). Rat tail collagen type I was from Cultrex.

GFP- and Cre-expressing adenoviruses were from University of Iowa, Gene Transfer Vector Core. For inducible expression of YAP and TAZ, cDNA for siRNA-insensitive Flag-hYAP1 wt, S94A (TEAD-binding mutant(Zhao et al., 2008)) and 5SA (LATS-mutant sites(Aragona et al., 2013)) and for Flag-mTAZ4SA(Azzolin et al., 2012) were subcloned in FUW-tetO-MCS, obtained by substituting the Oct4 sequence in FUW-tetO-hOct4 (Addgene #20726(Hockemeyer et al., 2008)) with a new multiple cloning site (MCS). This generated the FUW-tetO-wtYAP, FUW-tetO-YAPS94A, FUW-tetO-YAP5SA, FUW-tetO-TAZ4SA used throughout this study. FUW-tetO-MCS (empty vector) or FUW-tetO-EGFP plasmids were used as controls, as previously indicated(Cordenonsi et al., 2011).

For stable expression of GFP, we used pRRLSIN.cPPT.PGK-GFP.WPRE (gift of L. Naldini) lentiviral vector.

For Cre-excisable expression of rtTA, we used LV-CMV-rtTA-LoxP (Figures S4L-N), obtained by substituting the Cre cDNA in LV-CMV-Cre-LoxP with the cDNA of rtTA from FUdeltaGW-rtTA (Addgene #19780(Maherali et al., 2008)).

For Cre-excisable lentiviral vector containing the tetO-Flag-hYAP wt cassette, we used LV-tetO-YAP wt-LoxP (Figures S4L-N), obtained by substituting the CMV-Cre cassette in LV-CMV-Cre-LoxP with the tetO-Flag-hYAP wt cassette from FUW-tetO-Flag-hYAP1 wt.

All constructs were confirmed by sequencing.

siRNA transfections were done with Lipofectamine RNAi-MAX (Life technologies) in antibiotics-free medium according to manufacturer instructions. Sequences of siRNAs targeting murine *Yap* and *Taz* were as previously decribed (Azzolin et al., 2014).

DNA transfections were done with TransitLT1 (Mirus Bio) according to manufacturer instructions.

Lentiviral preparation

HEK293T cells (checked routinely for absence of mycoplasma contaminations) were kept in DMEM supplemented with 10% FBS (Life Technologies), Glutamine and Antibiotics (HEK medium). Lentiviral particles were prepared by transiently transfecting HEK293T with lentiviral vectors (10 micrograms/10 cm dishes) together with packaging vectors pMD2-VSVG (2.5 micrograms) and pPAX2 (7.5 micrograms) by using TransIT-LT1 (Mirus Bio) according to manufacturer instructions. Specifically, 60 μl of TransIT-LT1 reagent was diluted in 1.5 ml of Opti-MEM (Life Technologies) for each 10 cm dish, incubated with the vector DNA 15 min at RT and gently distributed over to the cell medium (dish contained about 10 ml of HEK medium). After 8 hr, HEK medium was changed. 48 hr post-transfection supernatant was collected, filtered through 0.45 micrometers and directly stored at -20°C; we did not concentrate viral supernatants. We also seldom measured the lentiviral titer of various supernatants used in our experiments using the QuickTiter Lentivirus Titer kit (lentivirus-associated HIV p24; Cell Biolabs) according to the manufacturer's protocol. As reference, viral supernatants were in the range 16 - 20 ng of p24/ml. This titer corresponds to a viral particle concentration of about $2x10^8$ particles/ml. As determined by PCR of integrated lentiviral DNA of HEK293T transduced with pRRL-EGFP, this roughly corresponds to $2x10^6$ transduction units (TU)/ml in the unconcentrated viral supernatant stock.

Lentiviral Infection of primary cells

As example, for a typical infection of a 3 cm dish containing primary cells, we used 500 μl of each unconcentrated viral supernatant diluted to 1x cell-specific medium in 2 ml final volume. For mammary gland cells, we mixed one volume (e.g., 500 μl/3.5 cm dish) of FUdeltaGW-rtTA supernatant, one volume of the FUW-tetO-YAP (or TAZ) supernatant, and added two volumes of serum-free MG medium with 2X concentrations of supplements. For neurons, for a 3.5 cm well, we mixed 500 μl of FUdeltaGW-rtTA with 250 μl of FUW-tetO-wtYAP (or negative controls) viral supernatants, 250 μl of HEK medium with 1.5 ml of serum-free Neurobasal medium with 2X B27.

Mammary gland dissociation

Primary MECs were isolated from the mammary glands of 8- to 12-week-old virgin C57BL/6J mice (unless otherwise specified), according to standard procedures (Stingl et

al., 2006). Mammary glands were minced and then digested with 6000 U/ml collagenase I (Life Technologies) and 2000 U/ml hyaluronidase (Sigma) in the DMEM/F12 (Life Technologies) at 37°C for 1 hour with vigorous shaking. The digested samples were pipetted, spun down at 1500 rpm for 5 min, and incubated 3 min in 0.64% buffered NH_4Cl (Sigma) in order to eliminate contaminating red blood cells. After washing with DMEM/F12 + 5% FBS, cells were plated for 1 hour at 37°C in DMEM/F12+5% FBS: in this way, the majority of fibroblasts attached to the tissue culture plastic, whereas mammary epithelial populations did not; MEC were thus recovered in the supernatant and pelleted. After washing in PBS/EDTA 0.02%, MECs were further digested with 0.25% trypsin (Life Technologies) for 5 min and 5 mg/ml dispase (Sigma) plus 100 μg/ml DNase I (Roche) for other 10 min. The digested cells were diluted in DMEM/F12+5%FBS and filtered through 40 μm cell strainers to obtain single cell suspensions cells and washed once in the same medium.

Matrigel culture of mammary colonies and organoids

After infection in 2D cultures and induction with doxycycline for 7 days, mammary cells were detached with trypsin and seeded at a density of 1,000 cells/well in 24-well ultralow attachment plates (Corning) in mammary colony medium (DMEM/F12 containing glutamine, antibiotics, 5% Matrigel, 5% FBS, 10 ng/ml murine EGF, 20 ng/ml murine bFGF, and 4 μg/ml heparin) containing doxycycline (2 μg/ml). Primary colonies were counted 14 days after seeding. To show the self-renewal capacity of yMaSCs independently of exogenous YAP/TAZ supply (i.e, independently of doxycycline administration), primary colonies were recovered from the MG-colony medium by collecting the samples and incubation with an excess volume of ice cold HBSS in order to solubilize Matrigel. After 1 hour, colonies were rinsed 3 times in cold HBSS by centrifugation at 1000 rpm for 5 min and incubated in trypsin 0.05% for 30 min to obtain a single cell suspension. Cells were counted and re-seeded at 1,000 cells/well in 24-well ultralow attachment plates in MG colony medium without doxycycline for further passaging.

For mammary organoid formation, primary colonies were recovered from MG colony medium in cold HBSS and transferred in 100% Matrigel. After Matrigel formed a gel at 37°C, MG organoid medium was added (Advanced DMEM/F12 supplemented with Hepes, GlutaMax, antibiotics, B27 1X, 50 ng/ml murine EGF, 20 ng/ml murine bFGF, 4 μg/ml heparin, 100 ng/ml Noggin and 1 μg/ml R-Spondin1). Note that at this step we do not

dissociate at single cell level the primary colonies but simply transfer them to organoid culture conditions. After few days, colonies started to form budding organoids. 64-75% (depending on the experiment) of yMaSC colonies evolved as organoids and were maintained and passaged without doxycycline. Also note that direct plating of MaSCs, LD control EGFP-infected, as well as YAP-infected cells, directly into organoid culture conditions did not result in any outgrowth, indicating that the intermediate step in colony culture conditions is required for organoid development. 2 weeks after seeding, organoids were removed from Matrigel, trypsin-dissociated and transferred to fresh Matrigel. Passages were performed in a 1:4-1:8 split ratio every 2 weeks. For analysis, colonies and organoids were recovered from Matrigel as before, and either embedded in OCT medium (PolyFreeze, Sigma) to obtain frozen sections for immunofluorescence or processed for protein or RNA extraction.

For the experiment depicted in Figure S1F, cells were plated into 96-well plate as single cells visually verified and their colony forming capacity was monitored. Based on the fluorescence of EGFP-positive LD cells (negative control cells), we measured that our rate of infection is about 50%.

For α- and *β-casein* induction (Figures 3F and S3G), Matrigel-embeded organoids derived from yMaSCs or MaSCs were treated with MG organoid medium supplemented with insulin (10 μg/ml) and dexamethasone (1 μg/ml) in the absence or presence of lactogenic hormone prolactin (5 μg/ml) for 7 days. Organoids were then recovered from Matrigel as before and processed for RNA extraction.

Cleared Mammary Fat Pad Transplantation

For induction of yMaSCs meant for in vivo injection (Figures 3H-J and S3I, J), adherent luminal differentiated cells were transduced for 48 hours with FUW-tetO-wtYAP in combination with stable rtTA- and EGFP-encoding lentiviruses to trace with EGFP fluorescence the generation of transgenic mammary glands from yMaSCs. For this, we mixed 500 μl of each FUdeltaGW-rtTA, pRRL-CMV-GFP and the FUW-tetO-wtYAP viral supernatants, diluted with 1.5 ml of serum-free MG medium with 2X concentrations of supplements, added to each 3 cm dish. Negative control LD cells were transduced with FUW-tetO-EGFP, rtTA and pRRL-CMV-GFP. After infection, cells were treated as before (washed, induced with doxycycline for 7 days in MG medium) and then injected in the cleared fat pads (see below). For the experiment of Figures 3K, L, cells were transduced as above and then cultured as clonal colonies and organoids; the latter were injected in the cleared fat pad of

recipient mice.

Cell aliquots or organoids resuspended in 10 μl PBS/10% Matrigel were injected into the inguinal mammary fat pads of NOD-SCID mice (Charles River), which had been cleared of endogenous mammary epithelium at 3 weeks of age. Animals were then administered doxycycline in the drinking water for 2 weeks and then maintained without doxycycline for additional 8-10 weeks. Transplanted mammary fat pads were examined for gland reconstitution by whole-mount staining, GFP native fluorescence and immunofluorescence on sections from paraffin-embedded biopsies. Only the presence of GFP-positive branched ductal trees with lobules and/or terminal end buds was scored as positive reconstitution. For whole-mount analysis of mammary glands, freshly-explanted glands were fixed in PFA 4% (2 hours) and ethanol 70% (overnight). Glands were rehydrated, stained overnight with hematoxylin, subsequently dehydrated in graded ethanols, cleared by incubation in benzyl-alcohol/benzyl benzoate (1:2; Sigma) and imaged.

Lineage tracing of neurons to yNSCs conversion

For the experiment depicted in Figures 4C-F and S4F, J, we obtained cortical neurons from *Syn1-Cre; R26-CAG-LSL-tdTomato/+* embryos (day 1 and 2 as described in experimental procedures). When indicated, these cells were transduced as described in experimental procedures (day 3). Cells were then treated with AraC/B27 containing medium as before and, after 7 days, switched to doxycycline containing-NSC medium to activate YAP expression and induce yNSC formation from tdTomato-positive neurons. *Syn1-Cre; R26-CAG-LSL-tdTomato/+* neurons transduced with FUdeltaGW-rtTA in combination with FUW-tetO-EGFP or FUW-tetO-YAPS94A never gave rise to any neurospheres and died after few days after transfer to NSC medium. Each embryo genotype was confirmed on tail biopsies post-brain dissociation; as separate negative controls, neurons derived from *R26-CAG-LSL-tdTomato/+* littermates (*Syn1-Cre* negative) were transduced with FUW-tetO-wtYAP and FUdeltaGW-rtTA viral supernatants, and never gave rise to tdTomato-positive yNSCs. These same neurons transduced with FUdeltaGW-rtTA in combination with FUW-tetO-EGFP or FUW-tetO-YAPS94A never gave rise to any neurospheres.

For the experiment depicted in Figure 4G, we obtained hippocampal neurons from *Thy1-Cre; R26-LSL-LacZ/+* embryos (day 1 and 2 as described in experimental procedures). These cells were transduced as described in experimental procedures (day 3). Cells were then treated with AraC/B27 containing medium as before and, after 7 days, switched to doxycycline

containing-NSC medium to activate YAP expression and induce yNSC formation from LacZ-positive neurons. *Thy1-Cre; R26-LSL-LacZ/+* neurons transduced with FUdeltaGW-rtTA in combination with FUW-tetO-EGFP or FUW-tetO-YAPS94A never gave rise to any neurospheres. Each embryo genotype was confirmed on tail biopsies post-brain dissociation; as separate negative controls, neurons derived from *R26-LSL-LacZ/+* littermates (Thy1-Cre negative) were transduced with FUW-tetO-wtYAP and FUdeltaGW-rtTA viral supernatants, and never gave rise to LacZ-positive yNSCs. These same neurons transduced with FUdeltaGW-rtTA in combination with FUW-tetO-EGFP or FUW-tetO-YAPS94A never gave rise to any neurospheres.

To validate the specificity of the neuronal drivers, primary NSCs were derived as below from *Syn1-Cre; R26-CAG-LSL-tdTomato/+* and *Thy1-Cre; R26-LSL-LacZ/+* embryos and we verified the negativity for tdTomato and LacZ, respectively (see Figures 4F, G)

For the experiment depicted in Figures 4H, S4G-I and S5B, we obtained cortical neurons from *Syn1-Cre; R26-LSL-rtTA-IRES-EGFP/+* embryos and hippocampal neurons from *Thy1-Cre; R26-LSL-rtTA-IRES-EGFP/+* (day 1 and 2 as described in experimental procedures). Each embryo genotype was confirmed on tail biopsies post-brain dissociation. These neurons were transduced with FUW-tetO-YAP wt alone (or FUW-tetO-empty vector or FUW-tetO-YAPS94A as negative controls). Cells were then treated with AraC/B27 containing medium as before and, after 7 days, switched to doxycycline containing-NSC medium to activate YAP expression and induce yNSC formation from GFP-positive neurons. For additional negative controls, neurons derived from *R26-LSL-rtTA-IRES-EGFP/+*littermates (*Thy1-Cre* negative) were treated as all the others, and never gave rise yNSCs.

For the experiment with excisable YAP vectors (Figures S4L-N), neurons were transduced (day 3) for 24 hours with LV-tetO-wtYAP-LoxP in combination with LV-CMV-rtTA-LoxP. Neurons were then treated with AraC/B27 containing medium as before and, after 7 days, switched to doxycycline containing-NSC medium to activate YAP expression.

Primary neural stem cells (NSCs) isolation and culture

Neural stem cells (NSCs) were isolated as previously reported (Ray and Gage, 2006) from the telencephalon of C57BL/6J E18 embryos or from mice of the indicated genotype. Telencephalons were minced and digested in trypsin 0.05% for 10 min at 37°C. The cell suspension was treated with DNaseI (Roche) and washed. NSCs were cultured in DMEM/F12 supplemented with 1% N2, 20 ng/ml murine EGF, 20 ng/ml murine bFGF, glutamine and

antibiotics. For passages, neurospheres were dissociated into single cells with TrypLE Express (Life Technologies).

NSCs/yNSCs transfection, infection and differentiation

Prior to transfection with siRNA, yNSCs were plated on fibronectin coated-plate in NSC medium, to allow a 2D culture; the next day, cells were transfected with siRNA and after 24 hours, replated in ultra-low attachment plates to allow neurosphere formation. Neurospheres were counted after 7 days from plating.

For adenoviral infection of wild-type (wt) or double $Yap^{fl/fl}$; $Taz^{fl/fl}$ NSCs (Figure 5K), single cells were plated in NSC medium containing adeno-Cre on ultra-low attachment plates and allowed to form neurospheres for 7 days.

For lentiviral infection of NSCs (Figure S4K), cells plated on fibronectin coated-plate in NSC medium, to allow a 2D culture; the next day, cells were infected with FUW-tetO-wtYAP and FUdeltaGW-rtTA viral supernatants. Negative controls were provided by neurons transduced with FUdeltaGW-rtTA in combination with FUW-tetO-EGFP. After 24 hours single cells were replated at a density of 20000 cells/well to allow neurosphere formation. Neurospheres were counted after 7 days from plating.

For neuronal differentiation (Choi et al., 2014), NSCs or yNSCs were cultured over a thin Matrigel layer. Differentiation medium was Neurobasal supplemented with 1X B27, glutamine.

For astrocyte differentiation (Bonaguidi et al., 2005), NSCs or yNSCs were plated on fibronectin coated-plate in NSC medium, to allow a 2D culture. The next day, medium was changed to DMEM (Life Technologies) containing 25 ng/ml LIF, 25 ng/ml BMP4, glutamine, and antibiotics for 2 weeks.

For oligodendrocyte differentiation (Hsieh et al., 2004), NSCs or yNSCs were plated on fibronectin coated-plate in NSC medium, to allow a 2D culture. The next day, medium was changed to Neurobasal (Life Technologies) containing 1x B27, 500 ng/ml IGF, 30 ng/ml T3, glutamine, and antibiotics for 2 weeks.

NSCs transplantation

P0 CD1 mice pups were used for cell transplantations. Pups were anesthetized by hypothermia (3 minutes) and fixed on ice-cold block during cell injection. Cells were resuspended in ice-cold HBSS ($5x10^4$ cells/μl) and injected into both hemispheres of neonatal mice with a 5μl-volume Hamilton syringe (2μl/injection). One month after the procedures, the

grafted animals were perfused with PBS and 4%PFA, and the brains were excised and processed for immunofluorescence.

Lineage tracing of pancreatic exocrine acinar cells

For the experiment depicted in Figures 6E-F (see also scheme in Figure S6I), we obtained acinar explants from 6 week-old *Ptf1a-CreERTM; R26-LSL-rtTA-IRES-EGFP/+; tetO-YAP*^{S127A} mice (Pan et al., 2013). Mice with this genotype were given tamoxifen by three daily i.p. injections of a 10 mg/ml solution in corn oil. 1 week after the last tamoxifen injection, mice were sacrificed and pancreas was dissociated to obtain *rtTA-IRES-EGFP*$^{+}$-labeled exocrine acinar cells. Primary pancreatic acinar cells were isolated and cultured as described in experimental procedures. For induction of pancreatic organoids, acinar explants were treated with 2 μg/ml doxycycline.

Matrigel culture of yDucts organoids

To show the self-renewal capacity of pancreatic organoids independently of exogenous YAP supply (i.e, independently of doxycycline administration), organoids were recovered from Matrigel or collagen cultures, trypsinized to obtain a single cell suspension and re-seeded in 100% Matrigel covered with pancreatic organoid medium. For analysis, organoids were recovered from Matrigel as before and processed for immunofluorescence or for protein or RNA extraction.

For the differentiation experiments shown in Figure S6R, yDucts were removed from Matrigel, trypsin-dissociated and seeded as single cells in Matrigel- coated (1:50) chamber slides. Cells were expanded in DMEM supplemented with 0.5% BSA, 1% ITS-X and 1x N2 and 50 ng/ml EGF and antibiotics for 5 days. For differentiation, cells were switched to DMEM/F12 supplemented with 1% ITS-X, 10 ng/ml bFGF, 10 mM nicotinamide, 50 ng/ml Exendin-4 and 10 ng/ml BMP4 and antibiotics for further 8 days. Cells were fixed in 4% PFA at Day 0 or Day 8 of differentiation and processed for immunofluorescence.

Culture of pancreatic ductal organoids (Ducts)

For culture of pancreatic duct-derived organoids, pancreatic ducts were isolated from the bulk of the pancreas as previously described(Huch et al., 2013) with minor modifications. The whole pancreas of 6- to 9-week-old mice of the indicated genotypes was grossly minced and digested by collagenase/dispase dissociation: DMEM medium (Life Technologies)

supplemented with collagenase type XI 0.012% (w/v) (Sigma), dispase 0.012% (w/v) (Life Technologies), 1% FBS (Life Technologies) and antibiotics at 37°C for 1 hour. Isolated pancreatic duct fragments were hand-picked under a dissecting microscope, carefully washed in DMEM medium and embedded in 100% Matrigel. After Matrigel formed a gel, pancreatic organoid medium (Advanced DMEM/F12 supplemented with 1x B27, 1.25mM N-Acetylcysteine, 10 nM gastrin, 50 ng/ml murine EGF, 100 ng/ml human Noggin, 100 ng/ml human FGF10, 10 mM Nicotinamide, 1 μg/ml R-Spondin1 and antibiotics) was added. Ductal fragments rapidly expanded to form cyst-like organoids within 5 days. Organoids were removed from Matrigel by incubation in ice cold HBSS, dissociated with trypsin 0.05% for 30 min to obtain a single cells suspension and reseeded in 100% fresh Matrigel. Organoid cultures were maintained for at least 9 months passaging every 10 days. For analysis, organoids were recovered from Matrigel and processed for immunofluorescence or for protein or RNA extraction.

For the experiments depicted in Figure S6C, pancreatic duct fragments were isolated from 9 weeks old *Yap$^{fl/fl}$; Taz$^{fl/fl}$* mice, embedded in 100% Matrigel and cultured as above. Organoids were passaged once every 10 days. After at least 3 months of culture, organoids were removed from Matrigel by incubation in ice cold HBSS, trypsin-dissociated and transduced with adenovirus encoding for CRE recombinase to induce Yap/Taz knockout (or with GFP-encoding adenovirus as control). Single cells were resuspended in 2 ml Advanced DMEM/F12, transduced for 2 hours at 37°C with adenovirus, washed in Advanced DMEM/F12 and seeded in 100% Matrigel. After Matrigel formed a gel, cells were maintained in pancreatic organoid medium and organoid formation capacity was morphologically monitored over a period of 10 days. Pancreatic ductal organoids obtained from *wt* mice were used as additional controls and treated as above.

Single-cell gene expression analysis during the conversion of pancreatic acinar cells to yDucts

For the experiment depicted in Figure 6G, we dissociated and harvested single cells from pancreatic cultures of the *R26-rtTA; tetO-YAP S127A* genotype during the YAP-induced conversion from acinar cells to yDucts, that is at: day 0 = starting acini (without doxycycline); day 2 = cultures that have experienced 48 hours of doxycycline; day 4 = cultures that have experienced 96 hours of doxycycline; yDucts (n= 12/13 each time point). Single cells were collected by pipetting into 200 μl- conical tubes and processed for total RNA extraction,

retro-transcription and pre-amplification with Single Cell-to-CT kit (Ambion), according to manufacturer's protocol. Specific target amplification products were then processed for qRT-PCR analysis on QuantStudio 6 Flex Real-time PCR System, 384-well (Applied Biosystems) with the following Taqman Gene Expression Assays: *18S* (Hs99999901_s1), *Amy2a5* (Mm02342486_mH), *Ptf1a* (Mm00479622_m1), *Nestin* (Mm00450205_m1), *Krt19* (Mm00492980_m1), *Sox9* (Mm00448840_m1), *Car2* (Mm00501576_m1), *Pdx1* (Mm00435565_m1), *Ctgf* (Mm01192933_g1), *Myc* (Mm00487804_m1), *Ccnd1* (Mm00432359_m1). Expression levels were normalized to *18S-rRNA* for each sample.

Immunofluorescence and microscopy

Immunofluorescences on PFA-fixed samples were performed as previously described(Cordenonsi et al., 2011). Briefly, samples were fixed 10 min at room temperature with 4% PFA solution. Slides were permeabilized 10 min at RT with PBS 0.3% Triton X-100, and processed for immunofluorescence using the following conditions: blocking in 10% Goat Serum (GS) in PBS 0.1% Triton X-100 (PBST) for 1 hr followed by incubation with primary antibodies (diluted in 2% GS in PBST) overnight at 4°C, four washes in PBST and incubation with secondary antibodies (1:200 in 2% GS in PBST) for 2 hours at room temperature. Samples were counterstained with ProLong-DAPI (Molecular Probes, Life Technologies) to label cell nuclei.

For immunofluorescence on mammary colonies and organoids (Figures 2B-C, 3B-E, S2, S3C-F), outgrowths freshly recovered from Matrigel were embedded in OCT tissue-freezing medium (PolyFreeze, Sigma) and frozen on dry ice. 8 µm cryostat sections for all types of organoids were cut at –20 °C. Sections were mounted on glass slides and dried for at least 30 min. The sections were then fixed with 4% formaldehyde for 10 min. After washing with PBS the sections were processed as described above. For immunofluorescence on pancreatic organoids or acini (Figures 6H and S6A, E), pancreatic acini and organoids were fixed overnight in PBS 4% PFA at 4°C, permeabilized with two washes in PBS 0.5% NP40 for 20 minutes at 4°C, followed by one wash in PBS 0.3% Triton X-100 for 20 minutes at room temperature. After two washes in PBS 0.1% Triton X-100 (PBST) for 15 minutes at room temperature, acini or organoids were blocked with two washes in PBST 10% GS for 1 hour at room temperature, and incubated overnight with primary antibodies. The following day, cells were washed twice in PBST 2% GS for 15 minutes at 4°C, and five more times in PBT 2% GS for 1 hour at 4°C. Secondary antibodies were incubated overnight. The third day, cells were washed five times in PBST for 15 minutes, incubated 20 min with DAPI solution and

mounted in glycerol.

For immunofluorescence on mammary and brain tissue, biopsies were fixed with PFA, paraffin-embedded and cut in 10 μm-thick sections. Sections were re-hydrated and antigen retrieval was performed by incubation in citrate buffer 0.01 M pH 6 at 95°C for 20 minutes. Slides were then permeabilized (10 min at RT with PBS 0.3% Triton X-100 for mammary sections and 10 min at RT with PBS 1% Triton X-100 for brain sections) and processed as described above.

Primary antibodies: anti-YAP (4912; 1:25) polyclonal antibody, anti-CNPase (5664S; 1:100) polyclonal antibody, anti-SOX2 (4900; 1:50) monoclonal antibody were from Cell Signaling Technology. anti-TAZ (anti-WWTR1, HPA007415; 1:25) polyclonal antibody, anti-α-SMA (A2547; 1:400) mouse monoclonal antibody and anti-amylase (A8273: 1:200) rabbit polyclonal antibody were from Sigma. anti-TUJ1 (anti β-III-tubulin; MMS435P-100; 1:500) mouse monoclonal antibody was from Covance. Anti-Synaptophysin (M0776; 1:200) mouse monoclonal antibody and anti-GFAP (Z0334; 1:1000) rabbit polyclonal antibody was from Dako. anti-Nestin (MAB353; 1:300) mouse monoclonal antibody and anti-SOX9 (AB5535; 1:200) and anti-NEUN (ABN78; 1:1000) rabbit polyclonal antibodies were from Millipore. anti-E-cadherin (610181; 1:1000) and anti-MASH1 (556604; 1:100) monoclonal antibody was from BD Biosciences. anti-K14 (Ab7800; 1:100) mouse monoclonal antibody, anti-NEUN (Ab177487; 1:100) rabbit monoclonal antibody, anti-K8 (Ab14053; 1:100) chicken polyclonal antibody and anti-GFP (Ab13970; 1:100) polyclonal antibody were from Abcam. anti-GFP (A6455; 1:100) rabbit serum was from Life Technologies. Anti-YAP (63.7; sc-101199; 1:200) mouse monoclonal antibody, anti-SV2a (E-8; sc-376234; 1:200) mouse monoclonal antibody, anti-p63 (H137, sc-8343; 1:50) polyclonal antibody and anti-Vimentin (Vim C-20, sc-7557-R; 1:100) rabbit polyclonal antibody were from Santa Cruz. anti-TAU (1:100) rabbit polyclonal antibody was from Axell. Anti-Ki67 (M3060; 1:100) rabbit monoclonal antibody was from Spring Bioscience. K19 was detected using the monoclonal rat anti-*Troma-III* antibody (DSHB; 1:50). Alexa-conjugated secondary antibodies (Life Technologies): Alexa-Fluor-488 donkey anti-mouse IgG (A21202); Alexa Fluor-568 goat anti-mouse IgG (A11031); Alexa-Fluor-647 donkey anti-mouse (A31571); Alexa Fluor-488 goat anti-mouse IgG_{2a} (A21131), Alexa Fluor-647 goat anti-mouse IgG_1 (A21240), Alexa Fluor-488 donkey anti-rabbit IgG (A21206), Alexa-Fluor-568 goat anti-rabbit IgG (A11036), Alexa-Fluor-647 donkey anti-rabbit IgG (A31573); Alexa Fluor-555 goat anti-chicken IgG (A21437). Goat anti-rat Cy3 (112-165-167) was from Jackson Immunoresearch.

For X-gal staining (Figure 4G), samples were permeabilized in PBS/NP-40 0.02%, fixed 1 hour in PFA 4% in PBS, washed twice in PBS/NP-40 0.02% and stained with the staining solution (X-gal (Sigma, B4252) 25 μg/ml, 4 mM potassium ferricyanide crystalline, 4 mM potassium ferricyanide trihydrate, 2mM $MgCl_2$, 0.02% NP-40 in PBS).

Confocal images were obtained with a Leica TCS SP5 equipped with a CCD camera. Bright field and native-GFP (or tdTomato) images were obtained with a Leica DM IRB inverted microscope equipped with a CCD camera (Leica DFC 450C). Live cell imaging was performed with a A1Rsi+ laser scanning confocal microscope (Nikon) equipped with NIS-Elements Advanced Research Software.

Western blot

Western blots were carried out as described in Ref. (Cordenonsi et al., 2011). Anti-YAP/TAZ (63.7; sc-101199) and anti-p63 (4A4; sc-8431) monoclonal antibodies were from Santa Cruz. anti-GAPDH (MAB347) monoclonal antibody was from Millipore. Anti-K14 (Ab7800) mouse monoclonal antibody and anti-K8 (Ab14053) chicken polyclonal antibody were from Abcam.

Quantitative Real-Time PCR (qRT-PCR)

Cells or tissues were harvested in TriPure (Roche) for total RNA extraction, and contaminant DNA was removed by DNase treatment. qRT-PCR analyses were carried out on retrotranscribed cDNAs with Rotor-Gene Q (Qiagen) thermal cycler and analyzed with Rotor-Gene Analysis6.1 software. Expression levels are always given relative to *Gapdh,* except for Figures S6B and S6Q in which expression levels were normalized to *18-S rRNA*. PCR oligo sequences for mouse samples are listed in our website http://www.bio.unipd.it/piccolo/protocols_and_tools.html.

Microarray experiments

For microarray experiments, Mouse Genome 430 2.0 arrays (Affymetrix, Santa Clara, CA, USA) were used. Total RNA was extracted using TriPure (Roche) from:

1) luminal differentiated mammary cells (3 replicates), organoids derived from yMaSCs (3 replicates), and MaSCs (3 replicates);

2) cortical neurons (3 replicates), yNSCs (from YAP wild type-transduced cortical neurons, passage 2; 3 replicates), and native NSCs (3 replicates);

3) pancreatic exocrine acini (4 replicates), yDucts (passage 10; 4 replicates), and Ducts (passage 10; 4 replicates).
RNA quality and purity were assessed on the Agilent Bioanalyzer 2100 (Agilent Technologies, Waldbronn, Germany); RNA concentration was determined using the NanoDrop ND-1000 Spectrophotometer (NanoDrop Technologies Inc.). RNA was then treated with DNaseI (Ambion). In vitro transcription, hybridization and biotin labeling were performed according to Affymetrix 3'IVT protocol (Affymetrix). As control of effective gene modulation and of the whole procedure, we monitored the expression levels of tissue-specific markers of differentiated cells or stem/progenitors by qRT-PCR prior to microarray hybridization and in the final microarray data.
All data analyses were performed in R (version 3.1.2) using Bioconductor libraries (BioC 3.0) and R statistical packages. Probe level signals were converted to log2 expression values using robust multi-array average procedure RMA(Irizarry et al., 2003) of Bioconductor *affy* package. Raw data are available at Gene Expression Omnibus under accession number GSE70174.
Global unsupervised clustering was performed using the function *hclust* of R *stats* package with Pearson correlation as distance metric and average agglomeration method. Gene expression heatmaps have been generated using the function *heatmap.2* of R *gplots* package after row-wise standardization of the expression values. Before unsupervised clustering, to reduce the effect of noise from non-varying genes, we removed those probe sets with a coefficient of variation smaller than the 90th percentile of the coefficients of variation in the entire dataset. The filter retained 4511 probe sets that are more variable across samples in any of the 3 subsets (i.e., mammary, neuron, and pancreatic).
Differentially expressed genes were identified using Significance Analysis of Microarray algorithm coded in the *samr* R package (as in Ref. (Tusher et al., 2001)). To identify differentially expressed genes, we selected those probe sets with an FDR ≤ 1%.
GO terms associated to genes coherently upregulated to Ducts and yDucts in comparison with acinar cells included RNA processing, positive regulation of cell cycle and control of cell death.

Mice

C57BL/6J mice and NOD-SCID mice were purchased from Charles River. Transgenic lines used in the experiments were gently provided by: Duojia Pan(Zhang et al., 2010) (*Yap1*$^{fl/fl}$ and *R26-LSL-LacZ*); Cedric Blanpain (*K8-CreERT2/R26-LSL-YFP)*(Van Keymeulen et al., 2011);

Pierre Chambon (*K14-CreERT2*)(Li et al., 2000); Doron Merckler (*Thy1.2-Cre*)(Dewachter et al., 2002); Ivan De Curtis and Riccardo Brambilla (*Syn1-Cre*)(Zhu et al., 2001); Giorgio Carmignoto (*R26-CAG-LSL-tdTomato*)(Madisen et al., 2010); Fernando Camargo (*tetO-YAP^{S127A}*)(Camargo et al., 2007). *Taz$^{fl/fl}$* and double *Yap$^{fl/fl}$; Taz$^{fl/fl}$* conditional knock-out mice were as described in Ref. (Azzolin et al., 2014). *Ptf1a-CreERTM* (stock #019378), *R26-LSL-rtTA-IRES-EGFP* (stock #005670) and *R26-rtTAM2* mice (stock #006965) were purchased from The Jackson Laboratory. Animals were genotyped with standard procedures(Morsut et al., 2010) and with the recommended set of primers. Animal experiments were performed adhering to our institutional guidelines as approved by OPBA and authorized by the Ministry of Health.

To obtain *Thy1-Cre; R26-LSL-LacZ/+* mice, we crossed *Thy1-Cre* hemizygous males with *R26-LSL-LacZ/LSL-LacZ* females. Littermate embryos derived from these crossings were harvested at E18-19 and kept separate for neurons/NSCs derivation; genotypes were confirmed on embryonic tail biopsies.

To obtain *Thy1-Cre; R26-LSL-rtTA-IRES-EGFP/+* mice, we crossed *Thy1-Cre* hemizygous mice with *R26-LSL-rtTA-IRES-EGFP/LSL-rtTA-IRES-EGFP* mice. Littermate embryos derived from these crossings were harvested at E18-19 and kept separate for neurons derivation; genotypes were confirmed on embryonic tail biopsies.

To obtain *Syn1-Cre* lineage tracing studies, we used *Syn1-Cre* hemizygous females (as transgene expression in male mice results in germline recombination(Rempe et al., 2006)) with *R26-LSL-rtTA-IRES-EGFP* or with *R26-CAG-LSL-tdTomato* homozygous males. Littermate embryos derived from these crossings were harvested at E18-19 and kept separate for neurons derivation; genotypes were confirmed on embryonic tail biopsies.

To obtain *R26-rtTAM2/+ ; tetO-YAP^{S127A}* mice, we crossed *R26-rtTAM2/+* mice with *tetO-YAP^{S127A}* mice. *R26-rtTAM2/+* littermates were used as negative control.

To obtain *Ptf1a-CreERTM; R26-LSL-rtTA-IRES-EGFP/+; tetO-YAP^{S127A}* mice, we crossed *Ptf1a-CreERTM; R26-LSL-rtTA-IRES-EGFP/LSL-rtTA-IRES-EGFP* mice with *tetO-YAP^{S127A}* mice. *Ptf1a-CreERTM; R26-LSL-rtTA-IRES-EGFP/+* littermates were used as negative control.

SUPPLEMENTAL REFERENCES

Aragona, M., Panciera, T., Manfrin, A., Giulitti, S., Michielin, F., Elvassore, N., Dupont, S., and Piccolo, S. (2013). A mechanical checkpoint controls multicellular growth through YAP/TAZ regulation by actin-processing factors. Cell *154*, 1047-1059.

Azzolin, L., Panciera, T., Soligo, S., Enzo, E., Bicciato, S., Dupont, S., Bresolin, S., Frasson, C., Basso, G., Guzzardo, V.*, et al.* (2014). YAP/TAZ incorporation in the beta-catenin destruction complex orchestrates the Wnt response. Cell *158*, 157-170.

Azzolin, L., Zanconato, F., Bresolin, S., Forcato, M., Basso, G., Bicciato, S., Cordenonsi, M., and Piccolo, S. (2012). Role of TAZ as mediator of Wnt signaling. Cell *151*, 1443-1456.

Bonaguidi, M.A., McGuire, T., Hu, M., Kan, L., Samanta, J., and Kessler, J.A. (2005). LIF and BMP signaling generate separate and discrete types of GFAP-expressing cells. Development *132*, 5503-5514.

Camargo, F.D., Gokhale, S., Johnnidis, J.B., Fu, D., Bell, G.W., Jaenisch, R., and Brummelkamp, T.R. (2007). YAP1 increases organ size and expands undifferentiated progenitor cells. Curr Biol *17*, 2054-2060.

Choi, S.H., Kim, Y.H., Hebisch, M., Sliwinski, C., Lee, S., D'Avanzo, C., Chen, H., Hooli, B., Asselin, C., Muffat, J.*, et al.* (2014). A three-dimensional human neural cell culture model of Alzheimer's disease. Nature *515*, 274-278.

Cordenonsi, M., Zanconato, F., Azzolin, L., Forcato, M., Rosato, A., Frasson, C., Inui, M., Montagner, M., Parenti, A.R., Poletti, A.*, et al.* (2011). The Hippo transducer TAZ confers cancer stem cell-related traits on breast cancer cells. Cell *147*, 759-772.

Dewachter, I., Reverse, D., Caluwaerts, N., Ris, L., Kuiperi, C., Van den Haute, C., Spittaels, K., Umans, L., Serneels, L., Thiry, E.*, et al.* (2002). Neuronal deficiency of presenilin 1 inhibits amyloid plaque formation and corrects hippocampal long-term potentiation but not a cognitive defect of amyloid precursor protein [V717I] transgenic mice. The Journal of neuroscience : the official journal of the Society for Neuroscience *22*, 3445-3453.

Hockemeyer, D., Soldner, F., Cook, E.G., Gao, Q., Mitalipova, M., and Jaenisch, R. (2008). A drug-inducible system for direct reprogramming of human somatic cells to pluripotency. Cell Stem Cell *3*, 346-353.

Hsieh, J., Aimone, J.B., Kaspar, B.K., Kuwabara, T., Nakashima, K., and Gage, F.H. (2004). IGF-I instructs multipotent adult neural progenitor cells to become oligodendrocytes. The Journal of cell biology *164*, 111-122.

Huch, M., Bonfanti, P., Boj, S.F., Sato, T., Loomans, C.J., van de Wetering, M., Sojoodi, M., Li, V.S., Schuijers, J., Gracanin, A., *et al.* (2013). Unlimited in vitro expansion of adult bi-potent pancreas progenitors through the Lgr5/R-spondin axis. The EMBO journal *32*, 2708-2721.

Irizarry, R.A., Hobbs, B., Collin, F., Beazer-Barclay, Y.D., Antonellis, K.J., Scherf, U., and Speed, T.P. (2003). Exploration, normalization, and summaries of high density oligonucleotide array probe level data. Biostatistics (Oxford, England) *4*, 249-264.

Li, M., Indra, A.K., Warot, X., Brocard, J., Messaddeq, N., Kato, S., Metzger, D., and Chambon, P. (2000). Skin abnormalities generated by temporally controlled RXRalpha mutations in mouse epidermis. Nature *407*, 633-636.

Madisen, L., Zwingman, T.A., Sunkin, S.M., Oh, S.W., Zariwala, H.A., Gu, H., Ng, L.L., Palmiter, R.D., Hawrylycz, M.J., Jones, A.R., *et al.* (2010). A robust and high-throughput Cre reporting and characterization system for the whole mouse brain. Nature neuroscience *13*, 133-140.

Maherali, N., Ahfeldt, T., Rigamonti, A., Utikal, J., Cowan, C., and Hochedlinger, K. (2008). A high-efficiency system for the generation and study of human induced pluripotent stem cells. Cell Stem Cell *3*, 340-345.

Morsut, L., Yan, K.P., Enzo, E., Aragona, M., Soligo, S.M., Wendling, O., Mark, M., Khetchoumian, K., Bressan, G., Chambon, P., *et al.* (2010). Negative control of Smad activity by ectodermin/Tif1gamma patterns the mammalian embryo. Development *137*, 2571-2578.

Pan, F.C., Bankaitis, E.D., Boyer, D., Xu, X., Van de Casteele, M., Magnuson, M.A., Heimberg, H., and Wright, C.V. (2013). Spatiotemporal patterns of multipotentiality in Ptf1a-expressing cells during pancreas organogenesis and injury-induced facultative restoration. Development *140*, 751-764.

Ray, J., and Gage, F.H. (2006). Differential properties of adult rat and mouse brain-derived neural stem/progenitor cells. Molecular and cellular neurosciences *31*, 560-573.

Rempe, D., Vangeison, G., Hamilton, J., Li, Y., Jepson, M., and Federoff, H.J. (2006). Synapsin I Cre transgene expression in male mice produces germline recombination in progeny. Genesis *44*, 44-49.

Tusher, V.G., Tibshirani, R., and Chu, G. (2001). Significance analysis of microarrays applied to the ionizing radiation response. Proceedings of the National Academy of Sciences of the United States of America *98*, 5116-5121.

Van Keymeulen, A., Rocha, A.S., Ousset, M., Beck, B., Bouvencourt, G., Rock, J., Sharma, N., Dekoninck, S., and Blanpain, C. (2011). Distinct stem cells contribute to mammary gland development and maintenance. Nature *479*, 189-193.

Zhang, N., Bai, H., David, K.K., Dong, J., Zheng, Y., Cai, J., Giovannini, M., Liu, P., Anders, R.A., and Pan, D. (2010). The Merlin/NF2 tumor suppressor functions through the YAP oncoprotein to regulate tissue homeostasis in mammals. Dev Cell *19*, 27-38.

Zhao, B., Ye, X., Yu, J., Li, L., Li, W., Li, S., Yu, J., Lin, J.D., Wang, C.-Y., Chinnaiyan, A.M.*, et al.* (2008). TEAD mediates YAP-dependent gene induction and growth control. Genes & development *22*, 1962-1971.

Zhu, Y., Romero, M.I., Ghosh, P., Ye, Z., Charnay, P., Rushing, E.J., Marth, J.D., and Parada, L.F. (2001). Ablation of NF1 function in neurons induces abnormal development of cerebral cortex and reactive gliosis in the brain. Genes & development *15*, 859-876.
