# Supplementary figures and images for: Induction of Expandable Tissue-Specific Stem/Progenitor Cells through Transient Expression of YAP/TAZ
Source: Cell Stem Cell. 2016 Dec 1;19(6):725–37. doi: 10.1016/j.stem.2016.08.009 (PMC5145813; doi:10.1016/j.stem.2016.08.009)

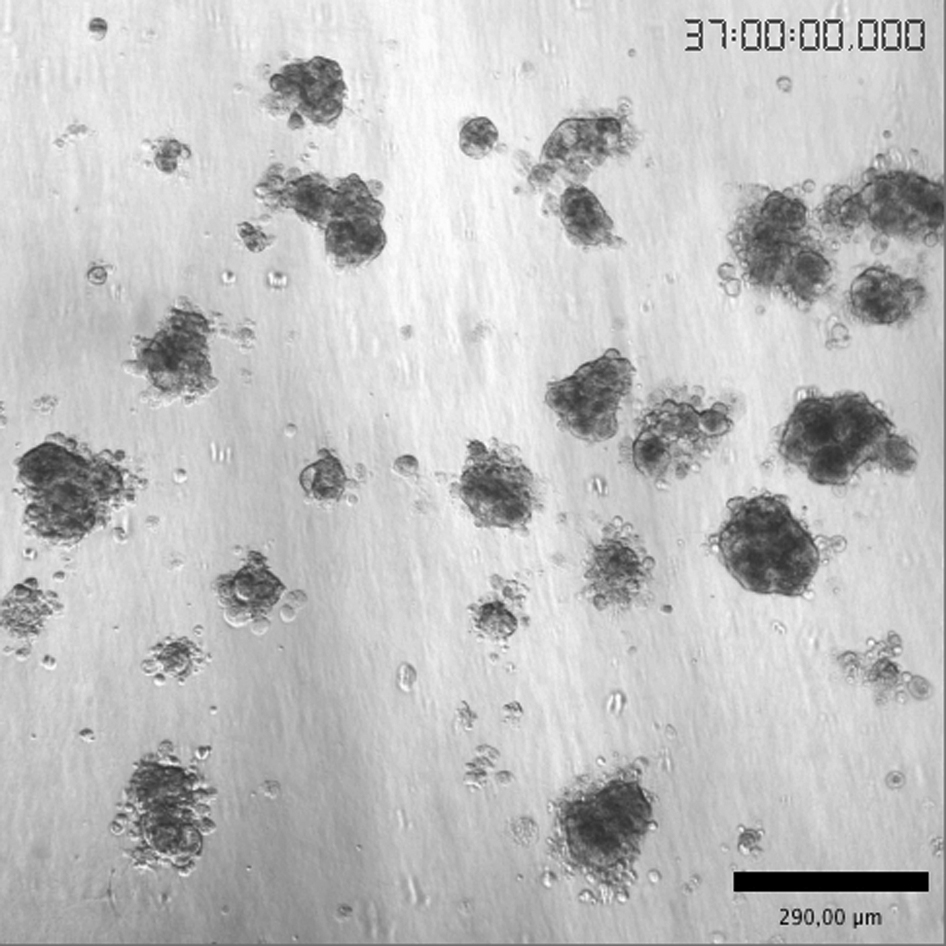

Supplement: Movie S1. Time-Lapse Movie Showing Conversion of Acini into yDucts, Related to Figure 6 — The movie shows the same field of whole pancreatic acini derived from R26-rtTA; tetO-YAPS127A mice during their conversion into cyst-like organoids at the indicated time points (hours) after doxycycline addition. [file mmc2.jpg]

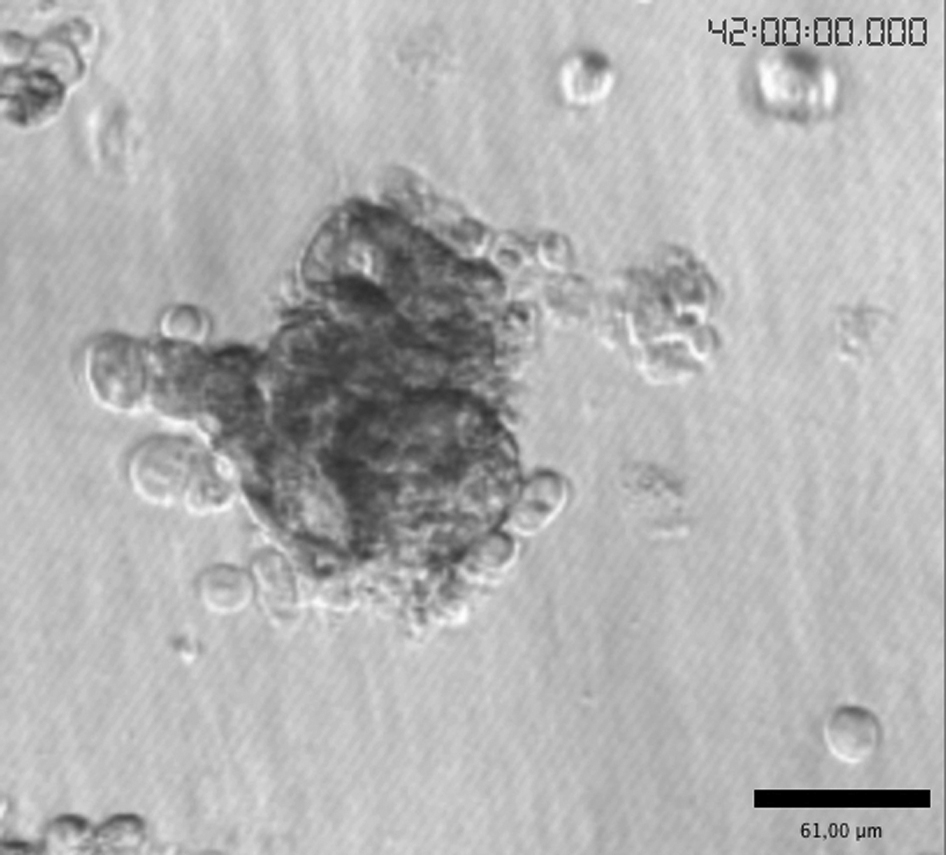

Supplement: Movie S2. Time-Lapse Movie showing Conversion of a Single Acinus into a yDuct, Related to Figure 6 — The movie shows a single pancreatic acinus derived from R26-rtTA; tetO-YAPS127A mice during conversion into cyst-like organoid at the indicated time points (hours) after doxycycline addition. [file mmc3.jpg]

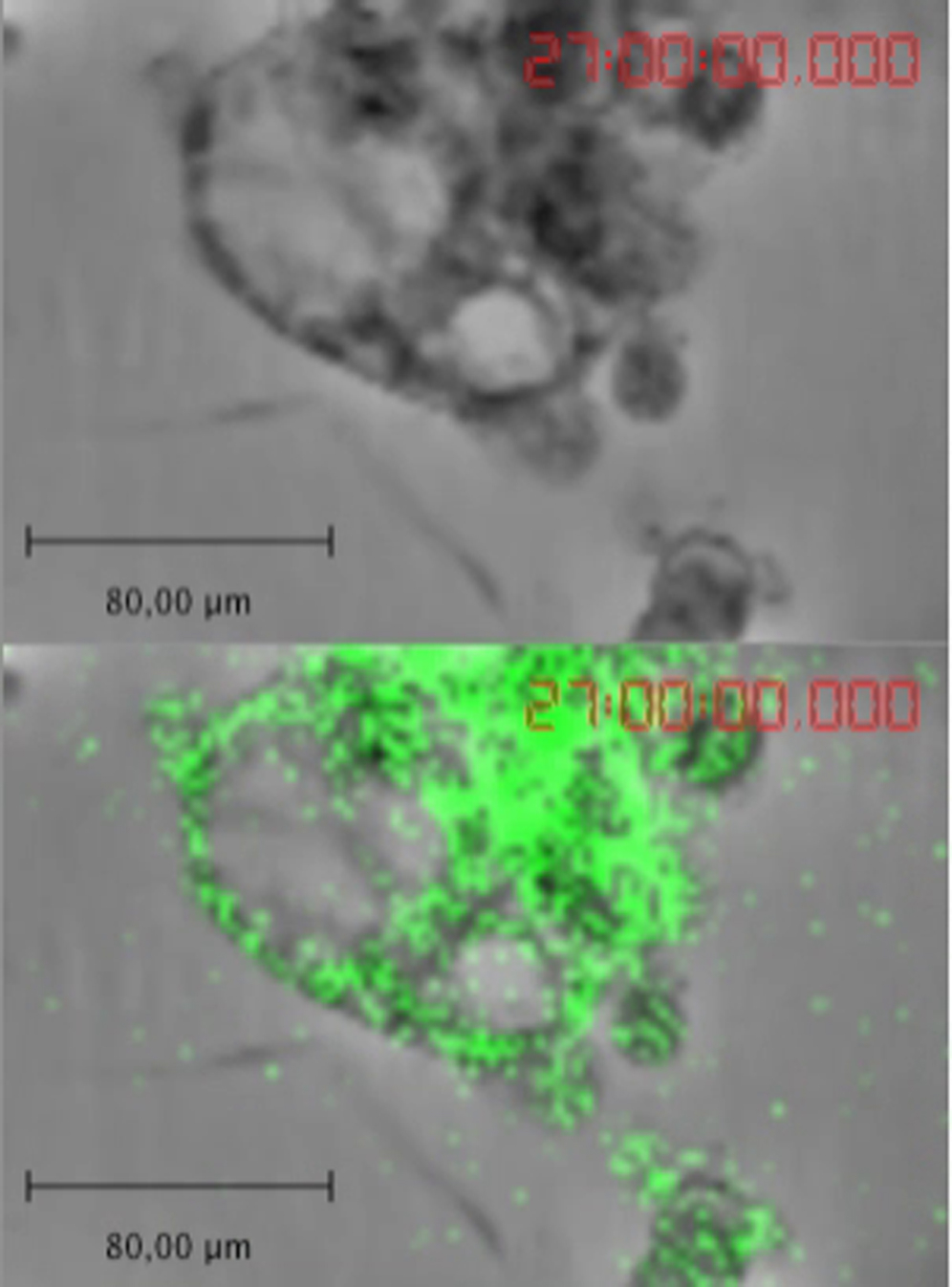

Supplement: Movie S3. Time-Lapse Movie Showing Conversion of a Lineage-Traced Single Acinus into a yDuct, Related to Figure 6 — Bright-field and GFP-fluorescence movie showing a single pancreatic acinus derived from Ptf1a-CreERTM; R26-LSL-rtTA-IRES-EGFP; tetO-YAPS127A mice during conversion into cyst-like organoid at the indicated time points (hours) after doxycycline addition. [file mmc4.jpg]
