# Supplementary material for: Induction of Expandable Tissue-Specific Stem/Progenitor Cells through Transient Expression of YAP/TAZ
Source: Cell Stem Cell. 2016 Dec 1;19(6):725–37. doi: 10.1016/j.stem.2016.08.009 (PMC5145813; doi:10.1016/j.stem.2016.08.009)
Supplement: Document S2. Article plus Supplemental Information [file mmc5.pdf]

Cell Stem Cell

Article

Induction of Expandable Tissue-Specific Stem/Progenitor Cells through Transient Expression of YAP/TAZ

Graphical Abstract

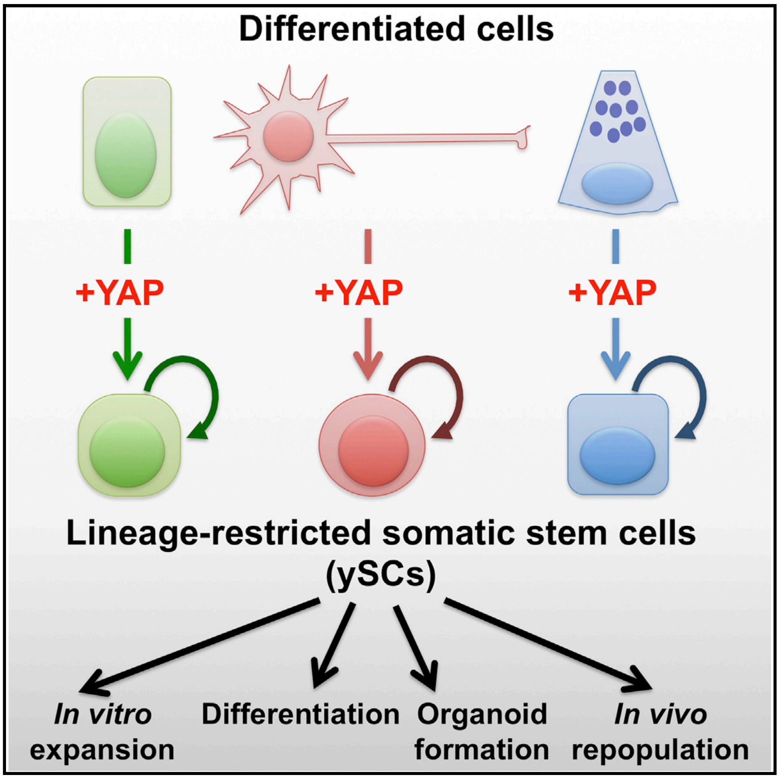

Highlights

- YAP/TAZ expression turns differentiated mammary gland cells into mammary stem cells
- YAP-induced MaSCs form organoids and have reconstitution capacity
- Induction of YAP in differentiated fetal neurons yields tripotent neural stem cells
- Pancreatic exocrine cells are also converted to progenitors by YAP expression

Authors

Tito Panciera, Luca Azzolin, Atsushi Fujimura, ..., Antonio Rosato, Michelangelo Cordenonsi, Stefano Piccolo

Correspondence

piccolo@bio.unipd.it

In Brief

Reprogramming and lineage conversions have highlighted the plasticity of differentiated cell states. Here Panciera et al. build on these principles by showing that expression of YAP/TAZ can convert a range of differentiated cells into somatic stem cells of the same tissue, respecting lineage restrictions.

Accession Numbers

GSE70174

Panciera et al., 2016, Cell Stem Cell *19*, 725–737
December 1, 2016 © 2016 The Authors. Published by Elsevier Inc.
http://dx.doi.org/10.1016/j.stem.2016.08.009

Cell Stem Cell
Article

Induction of Expandable Tissue-Specific Stem/Progenitor Cells through Transient Expression of YAP/TAZ

Tito Panciera,[1,5] Luca Azzolin,[1,5] Atsushi Fujimura,[1,5] Daniele Di Biagio,[1] Chiara Frasson,[2] Silvia Bresolin,[2] Sandra Soligo,[1] Giuseppe Basso,[2] Silvio Bicciato,[3] Antonio Rosato,[4] Michelangelo Cordenonsi,[1] and Stefano Piccolo[1,6,*]
[1]Department of Molecular Medicine, University of Padua School of Medicine, viale Colombo 3, 35126 Padua, Italy
[2]Department of Woman and Child Health, Haemato-Oncology Laboratory, University of Padua, via Giustiniani 3, 35128 Padua, Italy
[3]Center for Genome Research, Department of Life Sciences, University of Modena and Reggio Emilia, via G. Campi 287, 41100 Modena, Italy
[4]Istituto Oncologico Veneto IOV-IRCCS and Department of Surgery, Oncology, and Gastroenterology, University of Padua School of Medicine, via Gattamelata 64, 35128 Padua, Italy
[5]Co-first author
[6]Lead Contact
*Correspondence: piccolo@bio.unipd.it
http://dx.doi.org/10.1016/j.stem.2016.08.009

SUMMARY

The ability to induce autologous tissue-specific stem cells in culture could have a variety of applications in regenerative medicine and disease modeling. Here we show that transient expression of exogenous YAP or its closely related paralogue TAZ in primary differentiated mouse cells can induce conversion to a tissue-specific stem/progenitor cell state. Differentiated mammary gland, neuronal, and pancreatic exocrine cells, identified using a combination of cell sorting and lineage tracing approaches, efficiently convert to proliferating cells with properties of stem/progenitor cells of their respective tissues after YAP induction. YAP-induced mammary stem/progenitor cells show molecular and functional properties similar to endogenous MaSCs, including organoid formation and mammary gland reconstitution after transplantation. Because YAP/TAZ function is also important for self-renewal of endogenous stem cells in culture, our findings have implications for understanding the molecular determinants of the somatic stem cell state.

INTRODUCTION

Stem cells (SCs) display the capacity to self-renew when they divide and to generate a differentiated progeny. Somatic SCs operate in multiple adult organs for continuous tissue renewal or repair after injury. However, these cells are still mainly defined by operational definitions and cell surface markers, rather than the molecular traits that govern their special status (Blanpain and Fuchs, 2014). Unlimited availability of normal, somatic SCs will be critical for effective organ repopulation in regenerative medicine applications to understand SC biology and for disease modeling in the Petri dish. However, these efforts remain limited by the fact that SCs are rare and difficult to purify or expand from native tissues.

Direct conversion of terminally differentiated cells back into their corresponding tissue-specific SCs may represent an attractive approach to obtain somatic SCs. Indeed, several reports have recently highlighted a surprising plasticity in somatic cell fates because differentiated cells can return to a SC status under special conditions, such as tissue damage (Blanpain and Fuchs, 2014). However, the identity of the factors able to control the somatic SC status remains poorly understood, limiting the exploitation of such plasticity.

Dysregulation of the Hippo signaling pathway has been recently associated with cell fate plasticity. In tumors, activation of the Hippo pathway transcriptional effectors YAP/TAZ can reprogram non-stem cancer cells into cancer SCs (Cordenonsi et al., 2011). Genetic inactivation of the Hippo cascade induces liver overgrowth, in part by influencing liver cell fate and zonation (Lee et al., 2016; Yimlamai et al., 2014; Fitamant et al., 2015). Nuclear YAP/TAZ proteins are found in anatomical locations enriched in tissue SCs, possibly as a consequence of their regulation by structural and chemical signals associated with the SC niche (Piccolo et al., 2014). That said, genetic ablation of YAP and/or TAZ from several adult organs in mice, such as liver, pancreas, intestine, and mammary gland, revealed that these factors are surprisingly dispensable during normal tissue homeostasis. Strikingly, however, in those same tissues, YAP/TAZ become essential for organ regrowth after tissue damage or oncogenic transformation (Azzolin et al., 2014; Zanconato et al., 2016). This suggests that YAP/TAZ remain latent—and, thus, apparently dispensable—in normal adult tissues but are called into action to generate new stem cells.

With this background in mind, we considered the possibility that ectopic expression of YAP or TAZ may be instrumental to turn differentiated cells into somatic stem-like cells. Here we tested this hypothesis in vitro by using distinct paradigms of terminal differentiation; that is, luminal mammary gland cells, neurons, and pancreatic exocrine cells. We found that transient expression of YAP/TAZ indeed converts differentiated cells into cells displaying multiple features of their corresponding tissue-specific SCs. Notably, the ability of YAP/TAZ to impart an SC state pairs with their endogenous function in isolated native SCs, where they are essential for preserving organoid-forming

This is an open access article under the CC BY license (http://creativecommons.org/licenses/by/4.0/).

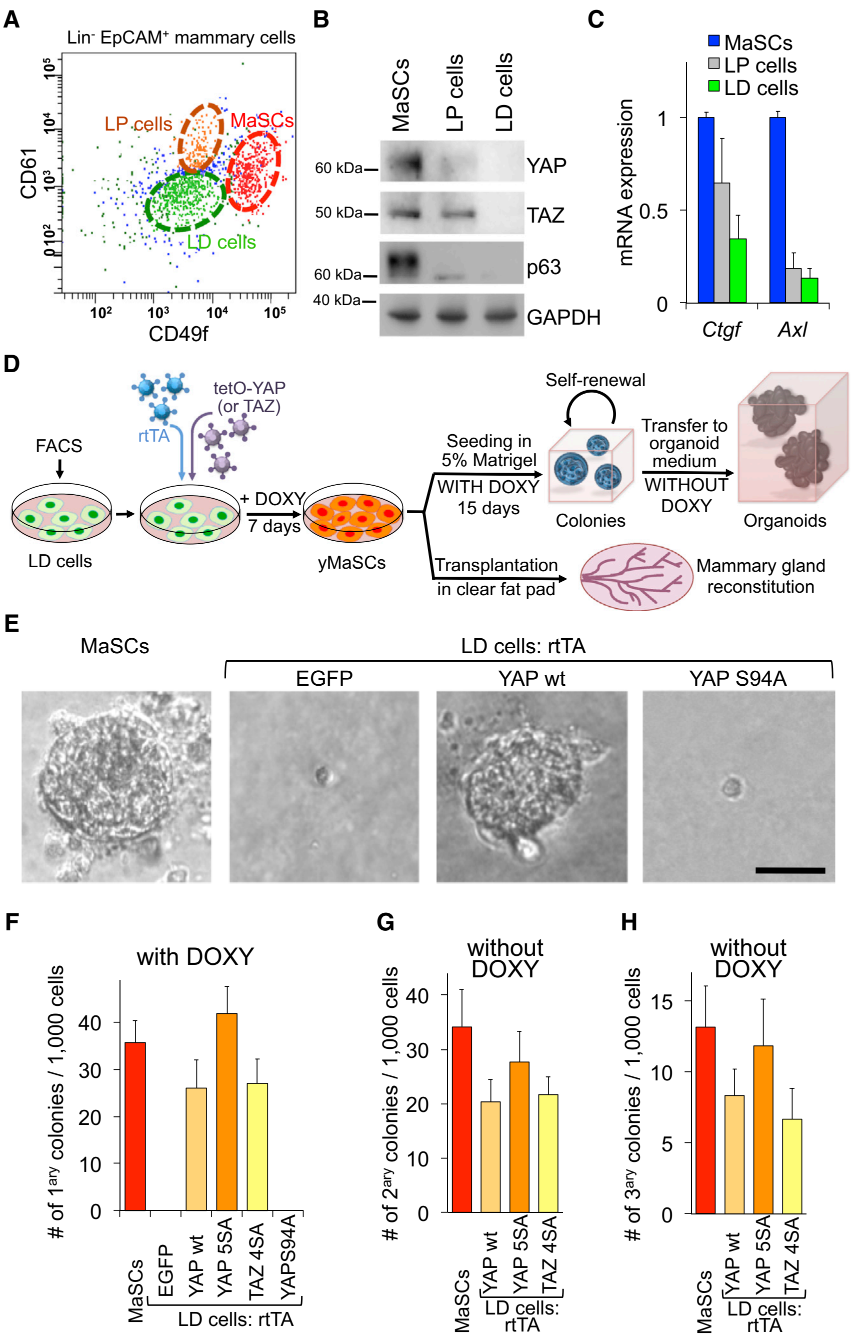

Figure 1. YAP and TAZ Convert Luminal Differentiated Cells in yMaSCs

(A) FACS profile showing the distribution of Lin–/EpCAM+ mammary cells according to their CD49f/CD61 antigenic profile. Three subpopulations were separated: a MaSC-enriched fraction ($EpCAM^{low}CD49f^{high}CD61^{+}$), luminal progenitors ($EpCAM^{high}CD49f^{low}CD61^{+}$), and luminal differentiated cells ($EpCAM^{high}CD49f^{low}CD61^{-}$). See also Figure S1A for the FACS pattern of EpCAM and Figures S1B–S1E for characterization of the three subpopulations.

(B) Western blots for YAP, TAZ, and the MaSC marker p63 in the indicated purified populations. GAPDH served as a loading control.

(C) qRT-PCRs for *Ctgf* and *Axl* in the indicated cell populations (mean + SD). The results are representative of three independent experiments (each using mammary glands from 20 mice) performed in triplicate.

(D) Schematic of the experiments performed with LD cells.

(E and F) Representative images (E) and quantifications (F) of mammary colonies formed by the indicated cells 15 days after seeding in mammary colony medium. The data in (F) are presented as mean + SD and are representative of five independent experiments, each with six technical replicates.

(G and H) Quantifications of secondary (G) and tertiary (H) colonies formed by primary mammary colonies after dissociation and re-seeding in mammary colony medium without doxycycline. The data are representative of three independent experiments performed with six technical replicates and presented as mean + SD.

See also Figure S1.

potential. Our work therefore reveals that a single factor can induce somatic stem cell features in cells of different lineages.

RESULTS

YAP/TAZ Revert Differentiated Cells of the Mammary Gland into MaSC-like Cells

The mammary gland represents a classic model system for the study of epithelial SCs and tissue regeneration. Remarkably, implantation of mammary gland SCs (MaSCs) into the mammary fat pad is sufficient to regenerate an entire ductal tree, with MaSCs contributing to both the luminal and myoepithelial lineages (Blanpain and Fuchs, 2014). Given that YAP/TAZ can reprogram non-stem mammary tumor cells into their corresponding cancer stem cells (CSCs) (Cordenonsi et al., 2011), we hypothesized that expression of YAP/TAZ may bestow stem-like characteristics on normal mammary cells as well.

To address this, we used fluorescence-activated cell sorting (FACS) to isolate terminally differentiated luminal cells (LD, $EpCAM^{high}CD49f^{low}CD61^{-}$) from dissected mouse mammary glands (Guo et al., 2012; Figure 1A). LD cells expressed markers of terminal differentiation and, in culture, were void of any proliferative potential and remained as single cells (Figures S1B–S1D). This differs from the MaSC-enriched or luminal progenitor (LP) fractions, which formed solid and cavitated colonies, respectively (Figure S1D). Endogenous YAP/TAZ proteins and their transcriptional targets *Ctgf* and *Axl* were highly expressed in the MaSC-containing population but at much lower levels in differentiated cells (Figures 1B and 1C).

To investigate whether ectopic expression of YAP or TAZ in LD cells could impart MaSC-like properties, FACS-purified LD cells

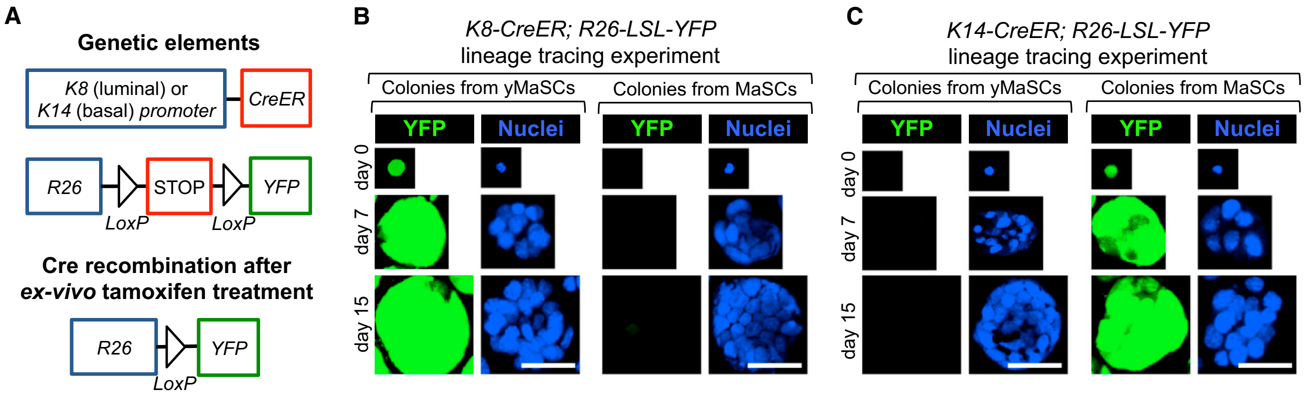

Figure 2. Lineage Tracing of yMaSCs

(A) Schematic of the genetic lineage tracing strategies to trace different mammary cell lineages.

(B and C) Immunostainings with anti-YFP of K8-CreER/R26-YFP-traced (B) or K14-CreER/R26-YFP-traced (C) cells during colony formation. Days indicate the time in mammary colony medium. Scale bars, 62 μm.

See also Figure S2.

were plated on collagen-coated dishes and transduced with doxycycline (Doxy)-inducible lentiviral vectors encoding for wild-type (WT) YAP or the activated versions of YAP and TAZ (i.e., YAP5SA or TAZ4SA, lacking inhibitory phosphorylation sites) (see the diagram in Figure 1D). As a control, cells were infected with an inducible EGFP vector. Transduced cells were cultured for 7 days in doxycycline-containing medium and then plated at clonogenic density in three-dimensional 5% Matrigel cultures (Experimental Procedures). Strikingly, cells expressing either YAP or TAZ formed solid colonies indistinguishable from those generated by MaSCs (Figures 1E and 1F) and very distinct from the cysts generated by LP cells (Figure S1D). EGFP-expressing control cells invariably remained as single cells without ever originating even a single colony in 33 experiments. As a further control, the expression of transcriptionally deficient YAPS94A (i.e., unable to interact with its DNA-binding partner TEAD) also had no effect.

We then asked whether YAP/TAZ expression converted luminal differentiated cells to a MaSC-like state. This includes the ability to form colonies that can be serially passaged. Indeed, YAP/TAZ-induced colonies, similarly to those generated from MaSCs, could form additional generations of colonies after single-cell dissociation (Figures 1G and 1H). Notably, colonies could be passaged even after expression of ectopic YAP had been turned off (by removing doxycycline) (Figures 1G, 1H and S3A). This suggests that transient expression of YAP/TAZ is sufficient to stably endow self-renewal potential to differentiated mammary cells. We thus designated the YAP/TAZ-induced “MaSC-like” cells as “yMaSCs.”

To verify whether the switch from LD to yMaSC could be recapitulated at the single-cell level, individual LD cells were seeded in 96-well plates (visually verified) and induced to express YAP. By monitoring the resulting outgrowths, we found that these individual cells formed solid colonies with high frequency (Figure S1F; 18.5% on average in the three independent experiments). From this experiment, we also noticed that this frequency of conversion, combined with the lack of colony-forming cells in controls (0%), argues against the hypothesis that yMaSCs arise from rare, contaminating, pre-existing stem/progenitors in our LD preparations.

Of note, we also found that overexpressing YAP in the endogenous MaSC-enriched cell population does not increase its colony-forming capacity (Figure S1G). In other words, even if rare contaminant MaSCs were present, then these would remain rare and not be expanded by YAP expression.

Validation of LD-to-yMaSC Conversion by Lineage Tracing

To validate the notion that YAP expression converts differentiated cells to an SC fate, we carried out reprogramming of LD cells purified from *K8-CreERT2; R26-LSL-YFP* mice (Figure 2A), allowing for a lineage tracing strategy to genetically label luminal cells (Van Keymeulen et al., 2011). For this experiment, we first FACS-purified LD cells (as in Figure 1A). After plating, cells were exposed to a pulse of tamoxifen to activate the YFP tracer exclusively in K8-positive cells and then infected with empty or YAP-expressing vectors. Colonies generated by YAP reprogramming of LD cells were entirely YFP-positive, confirming their origin from the luminal lineage (Figures 2B and S2A). As a control, we validated that the K8-CreERT2 tracing was restricted to luminal cells. Tamoxifen-treated MaSCs from the *K8-CreERT2; R26-LSL-YFP* mammary gland formed colonies that were exclusively YFP-negative (n = 154, 0% YFP+) (Figure 2B). These results also argue against the possibility that YFP-labeled yMaSCs could emerge from contaminating endogenous MaSCs.

The same conclusion was further validated by a complementary experiment in which we genetically labeled the basal/MaSC-enriched cell population by using *K14-CreERT2; R26-LSL-YFP* mice (Van Keymeulen et al., 2011; Figure 2A). LD cells and MaSCs were sorted by FACS as above and then treated with a pulse of tamoxifen to label K14-positive cells. YAP-reprogrammed LD cells purified from this genetic setup generated yMaSC colonies that were invariably void of any YFP expression (0%, n = 122) (Figure 2C). As a positive control, endogenous MaSCs traced by *K14-CreERT2* labeling formed YFP-positive

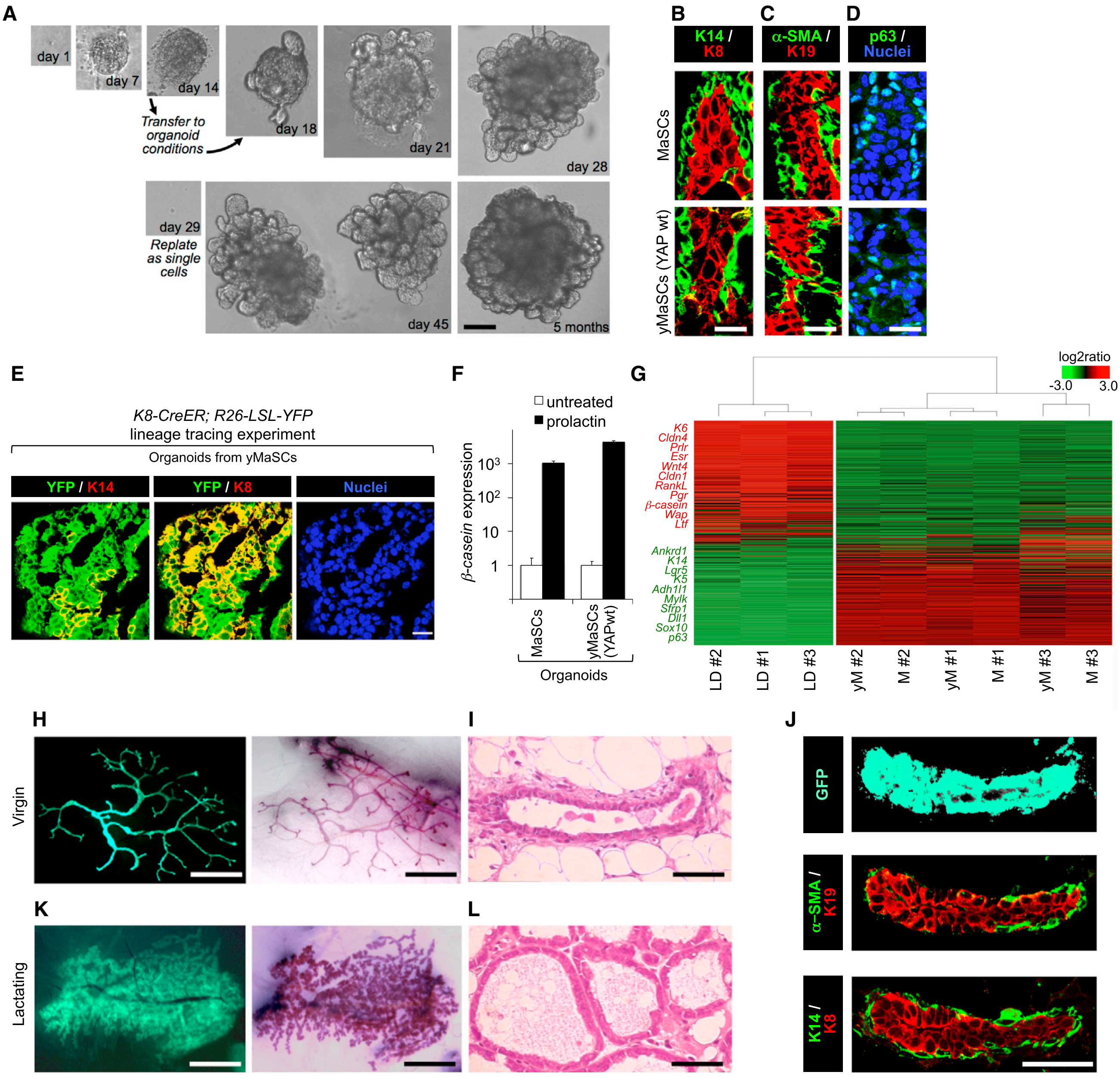

Figure 3. Characterization of yMaSCs

(A) Representative images of yMaSC outgrowths at the indicated time points. Until day 14, cultures were in mammary colony medium. After transfer to organoid conditions (see scheme in Figure 1D), 64%–75% of yMaSC colonies evolved as organoids and were maintained and passaged without doxycycline. Scale bar, 250 μm. See also Figure S3A for transgene expression.

(B–D) Organoids from MaSCs and yMaSCs (from YAP WT) expressed basal and/or stem (α-SMA, K14, p63) and luminal markers (K8, K19). Scale bars in IF pictures, 17 μm). See also Figures S3D–S3F for yMaSCs from phosphomutant YAP/TAZ.

(E) Immunostainings with anti-YFP combined with either anti-K14 or anti-K8 antibodies of yMaSC-derived organoids obtained from K8-CreER/R26-YFP-traced LD cells as in Figure 2A and Figure S2A. Scale bars, 49 μm.

(F) Organoids from MaSCs and yMaSCs (from YAP WT) expressed *β-casein* (qRT-PCR) when treated with prolactin. Data were normalized to *Gapdh* expression and are presented as mean + SD. The results are representative of two independent experiments performed in triplicate. See also Figure S3G.

(G) Unsupervised hierarchical clustering of gene expression profiles in LD cells, organoids from MaSCs (M), and organoids from yMaSCs (yM). Each column represents one separated biological sample. Genes are ordered according to the decreasing average expression level in LD cells. Representative genes up-regulated in LD cells (red) or in MaSC- and yMaSC-derived organoids (green) are shown on the left.

(H–J) Mammary gland reconstitution generated by stably GFP-expressing yMaSCs (from YAP WT) in virgin females. (H) Whole-mount images (left, native GFP fluorescence; right, hematoxylin staining). (I) Histological section. (J) Representative sections stained for GFP and the indicated markers. See Figures S3I and S3J for controls. Scale bars, 0.5 cm in (H) and 21 μm in (I) and (J).

(legend continued on next page)

colonies (Figure 2C). Collectively, several lines of evidence indicate that yMaSCs do not emerge from rare contaminating MaSCs pre-existing in our LD preparation. We conclude from these lineage-tracing experiments that YAP acts in differentiated cells to reprogram them into a MaSC-like state.

The Expansion, Differentiation, and Regenerative Potential of yMaSCs

We then examined whether yMaSCs are functionally equivalent to mammary SCs, as determined by additional characteristic properties of SCs, such as the ability to self-organize in vitro into mammary tissue-like structures, to differentiate along distinct lineages, and to regenerate a mammary tree in vivo after injection into a cleared mammary fat pad. For this, we sought to establish a long-term culture system that allows mammary SCs to form mammary gland-like structures in vitro. MaSC- and yMaSC-derived colonies were transferred and embedded into 100% Matrigel and overlaid with “organoid” medium (Sato et al., 2009) in the absence of doxycycline. Under these conditions, colonies underwent extensive budding and, by 2 weeks, grew into large epithelial organoids (Figure 3A; Figures S3B and S3C; see the legend of Figure 3A for quantification). Organoids derived from yMaSCs were indistinguishable in growth pattern, size, and frequency from those generated by natural MaSCs. yMaSC-derived organoids were dissociated, replated as single cells every 2 weeks, and cultured for at least 12 months (i.e., >25 passages) without changes in growth pattern, plating efficiency, and differentiation potential.

By histological examination, both MaSC and yMaSC organoids were composed by a stratified epithelium reminiscent of the histology of the mammary gland (E-cadherin-positive; Figure S3C). Internal cells surrounding a lumen-like cavity expressed differentiated luminal markers such as K8 and K19 (Figures 3B and 3C). Outer cells (either at external surfaces or bordering inner folds) displayed expression of basal, myoepithelial, and SC markers (K14, α-smooth muscle actin [α-SMA], p63) (Figures 3B–3D). yMaSCs derived from *K8-CreERT2; R26-LSL-YFP*-traced LD cells (as in Figure 2B) generated organoids with YFP-positive and K14-positive basal cells (Figures 3E and S2A), attesting to their origin from reprogrammed LD cells. Furthermore, addition of a lactogenic stimulus triggered expression of *α*- and *β-casein*, indicative of alveolar (milk-producing) cell differentiation (Figures 3F and S3G).

To molecularly characterize yMaSCs, we FACS-purified yMaSCs from organoids and evaluated the expression of luminal and basal and/or stem cell markers (Figure S3H). yMaSCs were remarkably similar to native MaSCs (freshly purified from the mammary gland) because they express basal markers (including myoepithelial markers such as *α-Sma* and *Myh11*) but not luminal markers (*Claudin1*, *K8*, *K18*, and *K19*). yMaSCs also express genes previously associated with various types of adult mammary SCs, including *ΔNp63*, *Lgr4/5/6*, and *Procr*, and all to levels comparable with MaSCs (Chakrabarti et al., 2014; Plaks et al., 2013; Wang et al., 2015).

By unsupervised hierarchical clustering of gene expression profiles, organoids from MaSCs or yMaSCs could not be distinguished (Figure 3G). Taken together, the results indicate that, similar to authentic MaSCs, yMaSCs display self-renewal potential, generate self-organizing epithelial structures reminiscent of the normal mammary gland, and retain multilineage differentiation ability.

Next we tested whether yMaSCs displayed mammary gland reconstituting activity. For this, FACS-purified LD cells were transduced with vectors encoding for EGFP and inducible wild-type YAP. Cells were treated with doxycycline for 7 days and then transplanted (10^3–10^4 cells) into the cleared mammary fat pads of non-obese diabetic (NOD)-severe combined immunodeficiency (SCID) mice. Strikingly, cells that had experienced transient expression of wild-type YAP had also acquired the ability to regenerate the mammary gland (25%, n = 16) (Figures 3H and 3I). Ductal tree and terminal end buds were regenerated when as few as 100 YAP-reprogrammed LD cells were implanted (33%, n = 6). As a control, LD cells transduced with the sole EGFP vector did not display any reconstituting activity at any inoculum dose (0%, n = 28, 10^2–5×10^4) (Figure S3I). Histological analyses revealed that the epithelial outgrowths obtained from yMaSCs were EGFP-positive and morphologically indistinguishable from those generated by endogenous MaSCs and consisted of a bilayered epithelium composed of a basal and/or myoepithelial layer (positive for K14 and α-SMA) overlaid by luminal cells (positive for K8 and K19) (Figures 3J and S3J).

To explore the reconstituting potential of a single yMaSC, we injected in the cleared fat pads single-cell derived organoids and found that these were also able to regenerate the mammary gland (33%, n = 6). Notably, when these mice were impregnated, reconstituted mammary glands generated a dense ductal system ending in clusters of milk-secreting alveoli, indicating that yMaSCs retain full differentiation potential in vivo (Figures 3K and 3L). We conclude from this collective set of experiments that transient expression of YAP/TAZ in differentiated cells of the mammary gland is able to convert them into bona fide MaSCs.

YAP/TAZ are not only instrumental for reprogramming of differentiated mammary cells but also endogenously required in MaSCs for preserving their self-renewal potential. Indeed, we generated organoids from either endogenous MaSCs or yMaSCs obtained from *Yap*$^{fl/fl}$*; Taz*$^{fl/fl}$ mice; deletion of endogenous YAP/TAZ by Adeno-Cre severely affected the ability of organoids to self-renew upon passaging (Figure S3K). Consistently, we also found that conversion to the ySC state was accompanied by activation of endogenous YAP/TAZ proteins. As shown in Figure S3L, induction of exogenous YAP in LD cells turned on expression of endogenous YAP and TAZ that remained expressed in ySC-derived organoids after ectopic YAP expression had been turned off (see Figure S3A for on-off control of exogenous YAP expression). We conclude that transient exposure to YAP/TAZ is sufficient to empower a self-sustaining loop of endogenous YAP/TAZ expression.

(K and L) Mammary gland reconstitution generated by yMaSC organoids in an impregnated female. (K) Whole-mount images (left, native GFP fluorescence; right, hematoxylin staining). (L) Histological section. Note that, upon gestation and lactation, the mammary gland is constituted by alveoli filled with milk. Scale bars, 0.5 cm in (K) and 21 μM in (L).
See also Figure S3.

CellPress

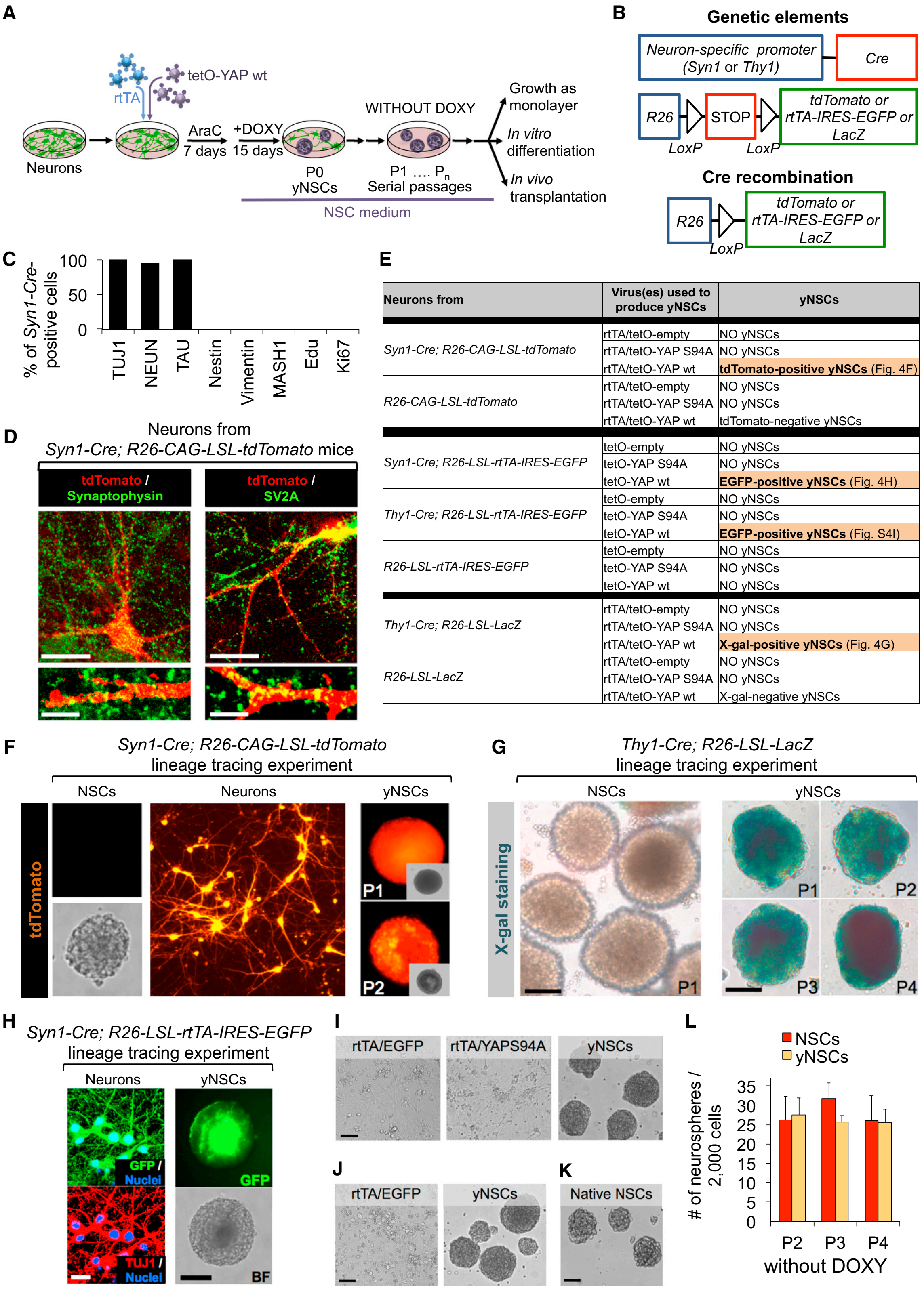

Neurons from	Virus(es) used to produce yNSCs	yNSCs
Syn1-Cre; R26-CAG-LSL-tdTomato	rtTA/tetO-empty	NO yNSCs
	rtTA/tetO-YAP S94A	NO yNSCs
	rtTA/tetO-YAP wt	**tdTomato-positive yNSCs** (Fig. 4F)
R26-CAG-LSL-tdTomato	rtTA/tetO-empty	NO yNSCs
	rtTA/tetO-YAP S94A	NO yNSCs
	rtTA/tetO-YAP wt	tdTomato-negative yNSCs
Syn1-Cre; R26-LSL-rtTA-IRES-EGFP	tetO-empty	NO yNSCs
	tetO-YAP S94A	NO yNSCs
	tetO-YAP wt	**EGFP-positive yNSCs** (Fig. 4H)
Thy1-Cre; R26-LSL-rtTA-IRES-EGFP	tetO-empty	NO yNSCs
	tetO-YAP S94A	NO yNSCs
	tetO-YAP wt	**EGFP-positive yNSCs** (Fig. S4I)
R26-LSL-rtTA-IRES-EGFP	tetO-empty	NO yNSCs
	tetO-YAP S94A	NO yNSCs
	tetO-YAP wt	NO yNSCs
Thy1-Cre; R26-LSL-LacZ	rtTA/tetO-empty	NO yNSCs
	rtTA/tetO-YAP S94A	NO yNSCs
	rtTA/tetO-YAP wt	**X-gal-positive yNSCs** (Fig. 4G)
R26-LSL-LacZ	rtTA/tetO-empty	NO yNSCs
	rtTA/tetO-YAP S94A	NO yNSCs
	rtTA/tetO-YAP wt	X-gal-negative yNSCs

Figure 4. YAP Converts Neurons in yNSCs

(A) Schematic of the experiments performed with hippocampal or cortical neurons.

(B) Schematic of the genetic lineage tracing strategy used to trace neurons ex vivo.

(legend continued on next page)

YAP Turns Neurons into Neural SC-like Cells

Neurons are post-mitotic cells that have been long considered refractory to any cell fate change. Endogenous YAP and TAZ proteins are highly expressed and transcriptionally active in neural stem cells (NSCs) but absent in neurons (Figures S4A–S4E), prompting us to determine whether ectopic expression of YAP in neurons was sufficient to convert them into NSCs. Primary cultures enriched in post-mitotic neurons (Figures 5A and S5A; see below) were obtained from the hippocampus or cortex from late mouse embryos (embryonic day 19 [E19]) or newborns. Cells were cultured in serum-free neuronal differentiation medium (Neurobasal with B27 containing vitamin A) also containing cytosine β-D-arabinofuranoside (AraC) for 7 days to support neuronal differentiation and eliminate proliferating cells (Figure 4A).

To follow the fate of post-mitotic neurons during YAP reprogramming, we adopted redundant lineage tracing strategies to stably label post-mitotic neurons. For this we prepared primary neurons from the brain of *Syn1-Cre* or *Thy1-Cre* transgenic mice expressing the Cre recombinase exclusively in post-mitotic neurons under the control of a neuron-specific promoter (Zhu et al., 2001; Dewachter et al., 2002) also bearing Cre-inducible tracers from the R26 locus (Figure 4B). We validated that expression of the lineage tracer was restricted to cells displaying unambiguous neuronal morphology and markers, whereas none of these traced cells was ever positive for NSCs and/or progenitor markers, as detailed in Figures 4C, 4D, and 4H and Figures S4F–S4H.

Primary cells were infected with lentiviral vectors encoding for reverse tetracycline transactivator (rtTA) and inducible wild-type YAP (Experimental Procedures). After AraC, neurons were shifted to NSC medium in the presence of doxycycline (see experimental outline in Figure 4A). Remarkably, after 2 weeks, neurosphere-like structures emerged from YAP-expressing neurons (P0 spheres) but never from neurons transduced with rtTA alone or rtTA combined with empty vectors or transcriptionally inactive YAPS94A (Figure 4E). Lineage-traced neurons from *Syn1-Cre; R26-CAG-LSL-tdTomato* or *Thy1-Cre; R26-LSL-LacZ* transgenic mice gave rise to tdTomato-positive or β-galactosidase (β-gal)-positive neurospheres, respectively (Figures 4F and 4G). Similarly, infection with tetO-YAP lentiviruses of neurons from mice bearing *Syn1-Cre* or *Thy1-Cre* and the *R26-LSL-rtTA-IRES-EGFP* reporter generated EGFP-positive yNSCs (Figures 4H and S4G–S4I). In contrast, NSCs derived from the same strains were invariably unlabeled (Figures 4F, left panel, and 4G, left panel). This confirms that, as originally described (Zhu et al., 2001; Dewachter et al., 2002), these drivers are not active in natural NSCs; hence, YAP-induced NSCs, or "yNSCs," originate from neurons rather than through amplification of pre-existing, contaminating, endogenous NSCs.

By monitoring the process more closely using lineage-traced neurons from *Syn1-Cre; R26-CAG-LSL-tdTomato,* we found that Doxy addition triggered YAP expression in about 30% of these neurons, and that, of these, 25% became positive for the NSC marker Nestin 2–3 days after switching them to the NSC medium (Figure S4J). This frequency of conversion, combined with the fact that no Nestin-positive neurons were present before YAP induction and no Nestin-positive neurons ever appeared in control cultures, argues against the possibility that yNSCs arose from rare pre-existing stem/progenitor cells in our neuronal preparations. This is in line with the above conclusions drawn from lineage tracing experiments.

Of note, we also found that overexpressing YAP in endogenous NSCs does not increase neurosphere-forming capacity (Figure S4K). Thus, even if rare contaminant NSCs were present, then it seems these would remain rare and not be expanded by YAP expression.

P0 spheres of yNSCs were transferred to new plates for further growth and could then be propagated for several passages as clonal outgrowths after single-cell dissociation, similarly to native NSCs (Figures 4I–4L). Lineage tracing was retained upon passaging (Figures 4F and 4G), indicating that YAP-reprogrammed neurons had acquired self-renewing properties.

The propagation of yNSCs as neurospheres did not require addition of doxycycline, indicating that transient exposure to

(C) We confirmed, in primary neuronal cultures prepared from the brain of *Syn1-Cre; R26-LSL-rtTA-IRES-EGFP* or *Syn1-Cre; R26-CAG-LSL-tdTomato* transgenic mice, that expression of the lineage tracer is indeed restricted to post-mitotic neurons expressing TUJ1 (100%, n = 635 cells), TAU (100%, n = 140 cells), and NEUN (95%, n = 252 cells). None of these traced cells were ever positive for the NSCs and/or progenitor markers Nestin, Vimentin, or MASH1 or for proliferation markers (5-Ethynyl-2'-deoxyridine [EdU], Ki67) (0%, n > 4,000 cells). See Figure S4F for representative images. See also Figure S4G for similar characterizations on *Thy1-Cre*-traced neuronal preparations.

(D) Immunofluorescence for the synaptic markers Synaptophysin and SV2A in cortical neurons derived from *Syn1-Cre; R26-CAG-LSL-tdTomato* mice. Scale bars, 11 μm (top) and 5.7 μm in magnifications (bottom).

(E) Table summarizing the results obtained in different lineage tracing experiments.

(F) Lineage tracing experiment showing that yNSCs originate from neurons. The images are bright-field and tdTomato-fluorescence pictures of primary cortical neurons from *Syn1-Cre; R26-CAG-LSL-tdTomato* mice and yNSCs derived from them (at the indicated passages). Neurospheres from *Syn1-Cre; R26-CAG-LSL-tdTomato* NSCs are presented as a negative control.

(G) Panels are X-gal stainings of yNSCs derived from hippocampal neurons from *Thy1-Cre; R26-LSL-LacZ* mice (scale bars, 210 μm) at the indicated passages. Neurospheres from *Thy1-Cre; R26-LSL-LacZ* NSCs (scale bar, 210 μm) are presented as a negative control.

(H) Lineage tracing experiment with the *Syn1-Cre* driver showing that yNSCs originate from Syn1-traced neurons. Left: immunostaining for GFP and TUJ1 in cortical neurons obtained from *Syn1-Cre; R26-LSL-rtTA-IRES-EGFP* mice. Right: bright-field and GFP fluorescence pictures of neurospheres derived from yNSCs obtained from the same neurons after transduction with doxycycline-inducible YAP. See also Figures S4G–S4I for similar results with the alternative lineage tracing strategy using *Thy1-Cre; R26-LSL-rtTA-IRES-EGFP* mice.

(I–K) Representative images of yNSCs neurospheres (second passage, P2) derived from hippocampal (I) or cortical (J) neurons. Images from negative control transduced neurons are shown as a reference (I and J). Neurospheres from native NSCs are presented for comparison (K). Scale bars, 210 μm.

(L) P1 yNSCs were dissociated to single cells and replated at clonal density for neurosphere formation in the absence of doxycycline for further passages (P2, P3, and P4). Native NSCs are presented for comparison. Graphs are quantifications of neurospheres formed by the indicated cells. Results are representative of at least eight (P2), six (P3), and three (P4) independent experiments performed in six replicates. Data are presented as mean + SD.

See also Figure S4.

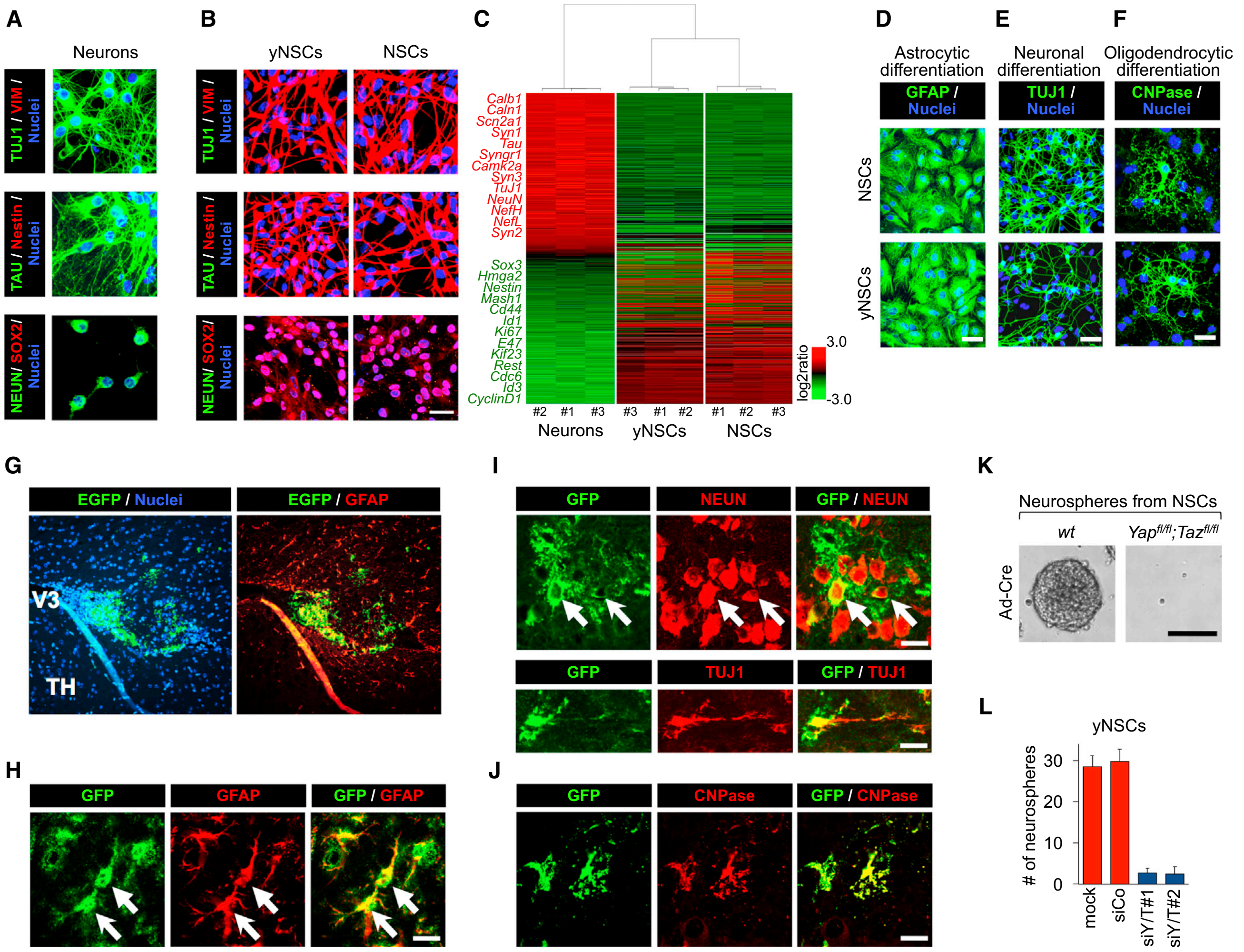

Figure 5. Characterization of yNSCs

(A and B) Immunofluorescence for the indicated markers in hippocampal neurons (A) and P3 yNSCs plated as a monolayer (B). Endogenous NSCs served as a positive control (scale bar, 23 μm). As shown in Figure S5B, Nestin-, SOX2-, and Vimentin-positive yNSCs derived from *Thy1-Cre; R26-LSL-rtTA-IRES-EGFP* neuronal preparations are co-stained with EGFP, confirming their origin from differentiated cells.

(C) Unsupervised hierarchical clustering of gene expression profiles in cortical neurons, yNSCs, and NSCs. Each column represents one separated biological sample. Genes are ordered according to the decreasing average expression level in neurons. Representative genes upregulated in neurons (red) or in NSCs and yNSCs (green) are shown on the left.

(D–F) YAP-induced yNSCs and endogenous NSCs as a positive control were plated and differentiated toward an astrocytic, neuronal, or oligodendrocytic fate (Experimental Procedures). Confocal images showing the astrocytic marker GFAP (D), the neuronal differentiation marker TUJ1 (E), and the oligodendrocytic marker CNPase (F) are displayed. The results are representative of three independent experiments performed in triplicate. Scale bars, 50 μm. For neuronal differentiation, see also Figures S5C–S5E, attesting acquisition of TAU and loss of Nestin expression.

(G–J) yNSCs were transduced with a constitutive EGFP-expressing vector and injected into the brains of recipient mice. Four weeks later, the brains were fixed and processed for immunofluorescence analyses. (G and H) Representative confocal images showing that injected yNSCs (GFP-positive) integrate in the brain parenchyma and massively differentiate into an astrocytic fate, as indicated by GFAP expression (arrows). V3, third ventricle; TH, thalamus. (I and J) Representative confocal images showing injected yNSCs (GFP-positive) differentiated in NEUN- and TUJ1-positive neurons (I, arrows) or CNPase-positive oligodendrocytes (J). Scale bars, 19 μm.

(K) Representative images of neurospheres from WT or *Yap*$^{fl/fl}$; *Taz*$^{fl/fl}$ NSCs transduced with Ad-Cre. Scale bar, 250 μm.

(L) yNSCs (passage 4 as neurospheres) were dissociated, plated on fibronectin-coated dishes, and transfected with the indicated siRNAs. The graph represents the quantification (mean ± SD) of neurospheres derived from the indicated cells.

See also Figure S5.

exogenous YAP is sufficient to induce self-renewal properties that are autonomously maintained. In line, as shown by experiments with a Cre-excisable tetO-YAP lentiviral vector, post-reprogramming deletion of the whole YAP-encoding viral cassette had no effects on yNSC maintenance (Figures S4L–S4N).

Characterization of yNSCs

Next we characterized yNSCs by marker gene expression using immunofluorescence, qRT-PCR, and gene profiling. As shown in Figures 5A and 5B and Figures S5A and S5B, yNSCs completely lost expression of the terminal differentiation markers present in

the original neurons (such as TUJ1, TAU, and NEUN) and, instead, expressed high levels of NSC markers (Nestin, SOX2, and Vimentin). Furthermore, we compared the transcriptome of parental neurons, yNSCs, and native NSCs and found that yNSCs completely lost their neuronal identity and acquired a gene expression profile closely similar to native NSCs (Figure 5C).

Neural SCs are defined as tripotent, as attested to by their ability to differentiate in astrocytes, neurons, and oligodendrocytes. We therefore examined the differentiation potential of yNSCs by placing them under appropriate culture conditions (Experimental Procedures). yNSCs could differentiate into astrocytes, neurons, and oligodendrocytes as defined by markers and morphology (Figures 5D–5F and S5C–S5E) and thus are indeed tripotent. To investigate the in vivo differentiation potential of yNSCs, we transplanted EGFP-labeled yNSCs in the brain of newborn mice (n = 5). Four weeks after transplantation, grafted yNSCs invariably lost Nestin positivity (Figure S5F) and mainly remained close to the injection site, where they primarily acquired expression of GFAP, indicative of astrocyte differentiation (Figures 5G and 5H). Injected yNSCs also differentiated into NEUN- and TUJ1-positive neurons or CNPase-positive oligodendrocytes (Figures 5I and 5J). Importantly, no tumor formation was ever observed after histological examination of the yNSCs-injected brain parenchyma. Thus, YAP induces conversion of neurons into cells that have functional properties similar to those of normal NSCs.

As for MaSCs, endogenous YAP/TAZ are essential to sustain the expansion of native NSCs in vitro because ex-vivo Adeno-Cre-mediated deletion of YAP/TAZ from *Yap*$^{fl/fl}$*; Taz*$^{fl/fl}$ NSCs blunted neurosphere formation (Figure 5K). We further established that the self-renewal properties of yNSCs are also sustained by reactivation of endogenous YAP/TAZ. Two lines of evidence support this conclusion. First, endogenous TAZ is induced in yNSCs and remains as such after doxycycline withdrawal and Cre-mediated excision of the tetO-YAP cassette (Figure S5G). Second, YAP/TAZ depletion in yNSCs by transfecting independent pairs of small interfering RNAs (siRNAs) greatly impairs their self-renewal properties (Figure 5L). These results raise an interesting parallel between the requirement of YAP/TAZ in native NSCs and induced yNSCs.

Ex Vivo Generation of Pancreatic Progenitors from Exocrine Cells

Pancreatic progenitors are rare in the normal pancreas but can be regenerated by differentiated acinar exocrine cells upon injury (Pan et al., 2013). Pancreatic progenitors are expandable in vitro as ductal organoids (Huch et al., 2013; Figures 6A and 6B), and, like MaSCs and NSCs, display nuclear and transcriptionally active YAP/TAZ and genetically require YAP/TAZ for their propagation (Figures S6A–S6C). Considering the intrinsic plasticity of acinar cells, we used them as a third reprogramming paradigm, asking whether a transient pulse of YAP expression could be sufficient to convert them into progenitors. To this end, we isolated pancreatic acini from *R26-rtTA; tetOYAP*^{S127A} adult mice and dissociated them to obtain a single cell preparation. Cells were plated in 100% Matrigel and cultured in the presence of doxycycline in pancreas organoid medium. In just a few days, acinar cells induced to express YAP, but not cells left without doxycycline, expanded as cyst-like organoids (Figures 6C and S6D). Acinar cells derived from control *R26-rtTA* mice remained as single cells or, more rarely, formed small cysts but never expandable organoids (Figure S6D). After initial derivation, YAP-induced organoids (or ‘‘yDucts’’) could be passaged for several months even in the absence of doxycycline and, thus, in the absence of exogenous YAP/TAZ (for at least 10 passages, 6 months). Individual organoids could be manually picked and expanded as clonal lines. By morphology, size, and growth pattern, organoids derived from converted acinar cells were comparable with those obtained from handpicked pancreatic duct fragments after whole pancreas dissociation (Huch et al., 2013; Figures 6B and 6D).

As an alternative strategy, we embedded whole pancreatic acini explanted from *R26-rtTA; tetOYAP*^{S127A} in collagen and cultured them under low-serum conditions known to preserve acinar cell identity ex vivo (Means et al., 2005). When treated with doxycycline to induce YAP expression (Figure S6E), pancreatic acini converted within a few days to ductal organoid structures and with high efficiency (>70%) (Movies S1 and S2; Figures S6F–S6H). As a control, acini lacking exogenous YAP expression (e.g., left without doxycycline; Figure S6H) remained as such and never converted to organoids. After transfer to 100% Matrigel-pancreatic organoid medium, the YAP-induced ducts, but not control acini, regrew into organoids and could be maintained for several passages after single-cell dissociation even in the absence of doxycycline (Figure S6G).

To validate that yDucts were indeed derived from differentiated exocrine acinar cells, we carried out genetic lineage tracing experiments using *Ptf1a-CreERTM; R26-LSL-rtTA-IRES-EGFP; tetO-YAP*^{S127A} mice (Figure S6I). In this genetic background, tamoxifen treatment of adult mice causes irreversible genetic tracing exclusively of pancreatic acinar cells (but not of endocrine, ductal, or centroacinar cells), as reported previously by others (Pan et al., 2013) and revalidated here (Figure S6J). After treatment, mice were kept without tamoxifen for 1 week, and then pancreata were explanted to prepare whole acini or single acinar cells that were cultured as above (see experimental outline in Figure S6I). These EGFP-positive cells never formed any organoid in the absence of doxycycline (Figures S6K and S6L). Instead, doxycycline-induced YAP expression caused the formation of expandable yDucts, that retained EGFP positivity over passaging, formally demonstrating their derivation from terminally differentiated exocrine cells (Figures 6E, 6F, S6M, and S6N; Movie S3). As a control of driver specificity in our culture conditions, organoids derived from endogenous ductal progenitors explanted from *Ptf1a-CreERTM; R26-LSL-rtTA-IRES-EGFP; tetO-YAP*^{S127A} pancreata were never labeled by EGFP (Figure S6O). This indicates that EGFP-traced yDucts emerge from exocrine cells and not from pre-existing ductal progenitors. In line with this conclusion, YAP overexpression is inconsequential in endogenous ductal progenitors for their clonogenic and organoid forming ability (Figure S6P), making unlikely the possibility that YAP expression might expand rare pre-existing contaminants.

We also performed a time course analysis of gene expression dynamics occurring at the single-cell level during reprogramming induced by tetO-Yap. As shown in Figure 6G, at the beginning of the experiment, cells expressed exocrine markers (*Amy*

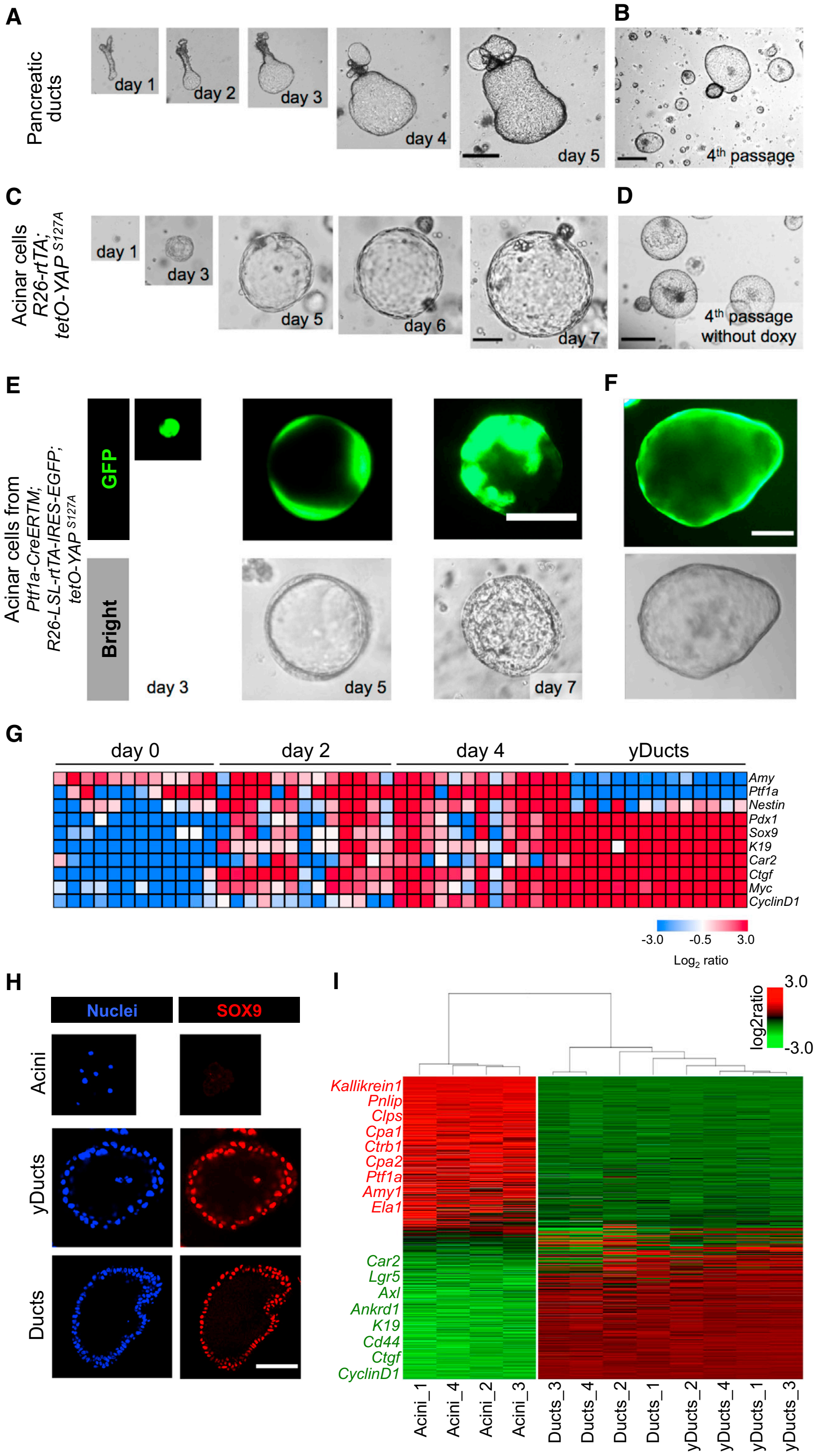

Figure 6. YAP Converts Pancreatic Acinar Cells to Duct-like Organoids

(A and B) Representative images of a pancreatic duct fragment growing in pancreatic organoid medium at the indicated times (A) and after four passages in fresh Matrigel (B). Pictures are representative of three independent experiments performed with four technical replicates. Scale bars, 290 μm.

(C and D) Serial images of a single acinar cell derived from *R26-rtTA; tetO-YAP*^{S127A} growing as cyst-like organoids at the indicated time points after Doxy addition (C) and after four passages in fresh Matrigel in the absence of Doxy (D). Pictures are representative of five independent experiments performed with four technical replicates. Scale bars, 70 μm in (C) and 290 μm in (D).

(E and F) Lineage tracing experiments using the *Ptf1a-CreERTM* driver. Images are bright-field and GFP fluorescence pictures of transgenic YAP-expressing exocrine cells at the indicated time points of Doxy treatment (E) and after passaging in absence of Doxy (F). See also Figure S6I for a schematic of the experiment. Scale bars, 70 μm in (E) and 130 μm in (F).

(G) Single-cell gene expression profile of pancreatic cultures of the *R26-rtTA; tetO-YAP*^{S127A} genotype during the YAP-induced conversion of acinar cells to yDucts. Rows are evaluated genes, and columns are individual cells. Day 0, starting acini (without doxycycline); day 2, cultures that experienced 48 hr of doxycycline; day 4, cultures that experienced 96 hr of doxycycline. The heatmap represents expression levels as log2 ratio normalized to *18S* rRNA.

(H) Organoids from duct fragments (Ducts, bottom, as in B) and YAP-induced organoids (yDucts, center) expressed the ductal marker SOX9 and were negative for the exocrine marker Amylase (data not shown) by immunofluorescence. Acinar cells (top) are shown as a control. Scale bar, 80 μm.

(I) Unsupervised hierarchical clustering of gene expression profiles in acini, yDucts, and Ducts. Each column represents one separated biological sample. Genes are ordered according to the decreasing average expression level in acini. Representative genes upregulated in acinar cells (red) or in Ducts and yDucts (green) are shown on the left.

See also Figure S6.

robustly on day 4 and then in yDuct cells (in line with what is shown in Movies S1, S2, and S3). On days 2 and 4 (but not in yDucts), most acinar cells retained concomitant expression of acinar markers and could thus be considered cells caught in transition. We noticed that progenitor markers were already present on day 2,

and *Ptf1a*) but not markers of pancreatic progenitors or ductal/centroacinar cells (*Pdx1*, *Sox9*, *K19*, and *Car2*), cell proliferation (*CyclinD1*), or the YAP targets *Ctgf* and *Myc* (Zanconato et al., 2015). After 2 days of doxycycline treatment, pancreatic progenitor markers were turned on in most cells and did so more before cells acquired expression of the proliferation marker *CyclinD1*, indicating that phenotypic conversion into a ductal progenitor state can be initially uncoupled from proliferation.

In section, yDuct-derived organoids appeared as epithelial monolayers surrounding a central cavity (Figure 6H). By

qRT-PCR and immunofluorescence, organoids lost markers of exocrine differentiation (*Ptf1a*, *α-amylase*, *Elastase*, and *CPA1*) and acquired expression of ductal markers (*K19*, *Sox9*, *Hes1*, and *Cd44*) and proliferative markers (*cMyc* and *CyclinD1*), all to levels comparable with those of native ductal organoids (Figures 6H and S6Q). To determine the extent of YAP-induced conversion of acinar cells and their molecular overlap with native ductal progenitors, we carried out transcriptomic analyses. As shown in Figure 6I, yDucts diverged from parental acinar cells to become ostensibly similar to bona fide pancreatic progenitors. Under differentiating conditions, yDuct-derived cells could be induced to re-express the differentiated exocrine marker CPA1 and to downregulate K19 (Figure S6R). When transplanted into the pancreas of NOD-SCID mice, yDucts remained as such and never formed any tumor (n = 6, data not shown), indicating that yDucts are indeed non-transformed and non-tumorigenic. Together, the results indicate that exocrine cells with a history of exposure to YAP acquired key molecular and biological features of ductal pancreatic progenitors.

DISCUSSION

Here we report that expression of a single factor into differentiated cells explanted from distinct tissues induces cells with functional and molecular attributes of their corresponding tissue-specific SCs that can be expanded ex vivo. The ySC state can be transmitted through cell generations without the need for continuous expression of ectopic YAP/TAZ, indicating that a transient activation of ectopic YAP or TAZ is sufficient to induce a heritable self-renewing state. Differently from induced pluripotent stem cells (iPSCs) or other reprogramming efforts, ySCs preserve a memory of the tissue of origin, expanding the current reprogramming paradigms by focusing on somatic stem cell generation from related cells of the same lineage.

Several lines of evidence support the notion that ySCs originate from conversion of differentiated cells rather than from amplification of rare pre-existing endogenous SCs. In particular, we used lineage tracing strategies employing established Cre drivers to label differentiated cells and follow their fate after YAP-induction. We found that ySCs indeed retained the genetic label specific of the original differentiated cells. Conversely, colonies and organoids emerging from native SCs were invariably unlabeled by the same genetic tracers. In the case of the mammary gland, we also used FACS to sort terminally differentiated cells from luminal progenitors and show that YAP can effectively operate on LD cells. Beyond lineage tracing, ySCs were induced with relatively high frequency after YAP expression, whereas no outgrowths emerged from differentiated cells expressing control vectors or transcriptionally inactive YAP, a scenario that argues against ySCs emergence from rare, pre-existing SCs. We also entertained the possibility that YAP overexpression may selectively expand rare native stem/progenitor cells. We consider this possibility also unlikely because YAP expression has no effect on the colony-forming capacity and expandability of native SCs. The latter finding is perhaps consistent with the fact that native SCs cultured ex vivo already contain transcriptionally active endogenous YAP/TAZ.

It is worth noting that, in mammary gland and pancreatic acinar cells, we were able to obtain transition to the corresponding tissue SCs starting from mature, adult differentiated cells, highlighting how YAP can imbue these lineages with a remarkable plasticity. However, we carried out our reprogramming experiments on fetal neurons because primary adult neurons cannot be effectively cultured ex vivo. Although they are post-mitotic, these early neurons may be particularly competent for YAP-induced reprogramming. Future work and technological advancements will be required to determine whether adult neurons can be reprogrammed by YAP similarly to fetal neurons.

Our procedure generates cells with normal SC traits as suggested by several lines of evidence: ySCs can be expanded over multiple passages as self-expanding organoids or neurospheres; ySCs readily generate a multilineage progeny reminiscent of the corresponding native SCs (for example, in the case of yMaSCs, reprogrammed SCs generate minigland-like organoids in vitro that, when transplanted in vivo, regenerate a normal ductal tree in the cleared fat pad); ySCs are not transformed and non-tumorigenic; and, at the transcriptional level, ySCs display remarkable overlaps with their native counterparts.

Lineage plasticity and reversion to an SC-like status rarely occur in normal tissues but are associated with tissue repair or oncogenic activation (Blanpain and Fuchs, 2014). Of note, genetic depletion of YAP and/or TAZ in several adult epithelia is inconsequential for normal homeostasis but, in fact, essential for regeneration, tumor growth (Zanconato et al., 2016), and, as shown here, for expansion of somatic SCs in vitro. It is thus tempting to propose that the path for lineage-specific reprogramming outlined here may be activated whenever natural, pathological, or ex vivo conditions demand generation and expansion of new SCs using differentiated cells as facultative SCs without losing tissue memory. Further work is required to validate that YAP reprogramming can occur in vivo and particularly in humans. If it does, it may be worth exploring means to exploit this path to facilitate tissue repair and regeneration in distinct tissues. From this perspective, the inability to cross lineage boundaries might represent a potential limitation for in vitro applications because some differentiated cells may not be readily accessible. However, if the same strategy could be applied directly in vivo, then the ability of a single factor to generate proliferative and multipotent SCs while retaining tissue memory would provide an advantage over other reprogramming and transdifferentiation strategies that employ either complex cocktails of transcription factors or the passing through an embryonic-like pluripotent state (Bar-Nur et al., 2015; Xu et al., 2015).

In conclusion, finding that YAP/TAZ, as a single factor, can reprogram distinct cell types into their corresponding tissue-specific SCs may have implications for regenerative medicine, for discovering still unknown determinants of somatic stemness, and, more broadly, for ad hoc expansion of somatic cells.

EXPERIMENTAL PROCEDURES

Primary Mammary Epithelial Cell Isolation and Induction of yMaSCs

Primary mammary epithelial cells (MECs) were isolated from the mammary glands of 8- to 12-week-old virgin C57BL/6J mice (unless otherwise specified) according to standard procedures (Stingl et al., 2006; see Supplemental Experimental Procedures for details). Animal experiments were performed adhering to our institutional guidelines, and approved by OPBA and the Ministry of Health. To separate various MEC subpopulations, cells were stained for

30 min at 4°C with antibodies against CD49f (phycoerythrin [PE]-Cy5, catalog no. 551129, BD Biosciences), CD29 (PE-Cy7, catalog no. 102222, BioLegend), CD61 (PE, catalog no. 553347, BD Biosciences), EpCAM (fluorescein isothiocyanate [FITC], catalog no. 118208, BioLegend), and lineage markers (allophycocyanin [APC] mouse lineage antibody cocktail, catalog no. 51-9003632, BD Biosciences) in DMEM/F12.

The stained cells were then resuspended in PBS/BSA 0.1% and sorted on a BD FACS Aria sorter (BD Biosciences) into LD cells, LP cells, and MaSCs.

Primary sorted subpopulations from FACS were plated on collagen I-coated supports and cultured in two dimensions in mammary gland (MG) medium (DMEM/F12 supplemented with glutamine, antibiotics, 10 ng/ml murine epidermal growth factor [EGF], 10 ng/ml murine basic Fibroblast growth factor (bFGF), and 4 μg/ml heparin with 2% fetal bovine serum [FBS]).

For induction of yMaSCs, LD cells were transduced for 48 hr with FUW-tetO-YAP or FUW-tetO-TAZ in combination with rtTA-encoding lentiviruses. As a (negative) control, LD cells were transduced with either FUW-tetO-EGFP (Figures 1E, 1F and S1F) in combination with rtTA-encoding lentiviruses. After infection, adherent cells were washed and treated with 2 μg/ml doxycycline for 7 days in MG medium for activating tetracycline-inducible gene expression (see scheme in Figure 1D) to obtain yMaSCs. After doxycycline treatment for 7 days in two-dimensional culture, yMaSCs were processed for further assays or analysis. Unless otherwise specified, yMaSCs were generated from wild-type YAP (FUW-tetO-WTYAP). For the experiment depicted in Figures 2B, 2C and S2A, we first FACS-purified LD cells and MaSC-enriched populations (using CD61 and CD49f as described previously for Figure 1A) from *K8-CreERT2; R26-LSL-YFP/+* or *K14-CreERT2; R26-LSL-YFP/+* virgin female mice. These cells were plated, and, after attachment, they were treated with 1 μM 4-hydroxy (4OH)-tamoxifen for 24 hr. Cells were then transduced for 48 hr with FUW-tetO-WTYAP in combination with stable rtTA-encoding lentiviral supernatant. Negative control cells were provided by LD cells transduced with FUW-tetO-MCS (empty vector) in combination with rtTA-encoding lentiviral supernatants. After infection, cells were washed and treated with doxycycline in MG medium as above.

Primary Neuron Isolation and Induction of yNSCs

Neurons were prepared from hippocampi or cortices of late (E18–19) embryos or post-natal day 1 (P1) pups as described previously (Han et al., 2008). Briefly, hippocampi and cortices were dissected under the microscope in ice-cold Hank's balanced salt solution (HBSS) as quickly as possible, incubated with 0.05% trypsin (Life Technologies) for 15 min at 37°C, and, after trypsin blocking, resuspended in DMEM/10% FBS supplemented with 0.1 mg/ml DNase I (Roche) and mechanically dissociated by extensive pipetting. Cells were then plated on poly-L-lysine-coated wells in DMEM supplemented with 10% FBS, glutamine, and antibiotics for hippocampal neurons or in DMEM/Neurobasal (1:1) supplemented with 5% FBS, 1× B27, glutamine, and antibiotics for cortical neurons (day 1). After 24 hr (day 2), the medium of both hippocampal and cortical preparations was changed to fresh DMEM/Neurobasal (1:1) supplemented with 5% FBS, 1× B27, glutamine, and antibiotics. For reprogramming experiments, neurons were infected on the following day (day 3) with FUW-tetO-WTYAP and FUdeltaGW-rtTA viral supernatants. Negative controls were provided by neurons transduced with FUdeltaGW-rtTA alone or in combination with FUW-tetO-EGFP or FUW-tetO-MCS (empty vector). After 24 hr (day 4), the medium was changed, and cells were incubated in Neurobasal medium supplemented with 1× B27, glutamine, antibiotics, and 5 μM AraC (Sigma) for an additional 7 days, at the end of which well differentiated, complex network-forming neurons were visible. To induce yNSCs formation, treated neurons were switched to NSC medium (DMEM/F12 supplemented with 1× N2, 20 ng/ml murine EGF, 20 ng/ml murine bFGF, glutamine, and antibiotics) and doxycycline for activating tetracycline-inducible gene expression. After 7 days, fresh doxycycline was added. Sphere formation was evident upon YAP induction after 14 days of doxycycline treatment. Spheres (P0 spheres) were mechanically detached from the plates by tapping and flushing (not by trypsinizing), transferred into a 15-ml plastic tube, and left to sediment (usually 5 min). After discarding the supernatant, spheres were dissociated with 1-–2 ml of TrypLE Express (Life Technologies) and mechanical pipetting. TrypLE Express was then diluted 1:5 in NSC medium, and cells were centrifuged and resuspended in NSC medium without doxycycline. For the successive passages, spheres were harvested and dissociated, and yNSCs were routinely cultured and passaged without doxycycline in NSC medium as for normal NSCs.

Pancreatic Acinar Cell Isolation and Induction of yDucts

Primary pancreatic acini were isolated from the pancreata of 6- to 9-week-old mice according to standard procedures (Means et al., 2005). Digested tissue was filtered through a 100-μm nylon cell strainer. The quality of isolated acinar tissue was checked under the microscope. For culture of entire acini, explants were seeded in neutralized rat tail collagen type I (Cultrex)/acinar culture medium (1:1) (Means et al., 2005), overlaid with acinar culture medium (Waymouth's medium [Life Technologies] supplemented with 0.1% FBS [Life Technologies], 0.1% BSA, 0.2 mg/ml soybean trypsin inhibitor [SBTI], 1× insulin-transferrin-selenium-ethanolamine [ITS-X] [Life Technologies], 50 μg/ml bovine pituitary extract [BPE] [Life Technologies], 1μg/ml dexamethasone [Sigma], and antibiotics) when collagen formed a gel. For culture of isolated acinar cells, acini were further digested in 0.05% trypsin for 30 min at 37°C to obtain a single-cell suspension. Single acinar cells were plated in 100% Matrigel. When Matrigel formed a gel, cells were supplemented with pancreatic organoid medium (advanced DMEM/F12 supplemented with 1× B27, 1.25 mM N-acetylcysteine, 10 nM gastrin, 50 ng/ml murine EGF, 100 ng/ml human Noggin, 100 ng/ml human FGF10, 10 mM nicotinamide, 1 μg/ml R-Spondin1, and antibiotics) supplemented with 0.2 mg/ml SBTI. For induction of pancreatic organoids, entire acini or single acinar cells of the indicated genotypes cells were seeded in medium supplemented with 2 μg/ml doxycycline. Negative control cells were cultured under the same conditions in the absence of doxycycline. Cells were treated with 2 μg/ml doxycycline for 7 days, and organoid formation was morphologically followed.

ACCESSION NUMBERS

The accession number for the Affymetrix data reported in this paper is GEO: GSE70174.

SUPPLEMENTAL INFORMATION

Supplemental Information includes Supplemental Experimental Procedures, six figures, and three movies and can be found with this article online at http://dx.doi.org/10.1016/j.stem.2016.08.009.

AUTHOR CONTRIBUTIONS

T.P. carried out the experiments on neurons and exocrine pancreas; A.F., on neurons; and L.A., on the mammary gland cells. C.F. and G.B. performed the sorting experiments. S. Bresolin, G.B., and S. Bicciato performed microarrays and biostatistics. D.D.B. performed molecular biology and Ifs. A.R. performed mouse surgery and helped with transplantation. S.P. conceived the initial hypothesis and experimental design. M.C. and S.P. planned, discussed, and organized the work. S.P., M.C., L.A., T.P., and A.F. wrote the manuscript.

ACKNOWLEDGMENTS

We thank O. Wessely, G. Martello, S. Dupont, and F. Zanconato for comments; V. Guzzardo for histology; C. Mucignat for advice on brain stainings; A. Migliorati, P. Raffa, and V. Di Iorio for help with live cell imaging; D.J. Pan, F. Camargo, C. Blanpain, G. Carmignoto, P. Chambon, D. Merkler, I. De Curtis, and R. Brambilla for gifts of mice; and L. Naldini for plasmids. We are indebted to P.G. Pellici and A. Santoro for advice on mammary fat pad transplantation, M. Forcato for help with bioinformatic analyses, and V. Broccoli and A. Sessa for advice on NSC transplantation. *R26-rtTA*, *R26-LSL-rtTA-IRES-EGFP*, and *Ptf1a-CreERTM* mice were purchased from The Jackson Laboratory, where they were deposited by R. Jaenisch, A. Nagy, and C. Wright, respectively. L.A. is supported by a fellowship from the Italian Association for Cancer Research (AIRC). A.F. is the recipient of an Astellas Foundation for Research on Metabolic Disorders fellowship and Japan Society for the Promotion of Science fellowship. This work is supported by a Fondazione Città della Speranza grant and CARIPARO Bando Ricerca Pediatrica (to G.B.), by AIRC Special Program Molecular Clinical Oncology "5 per mille" and an AIRC PI grant (to S.P.),

and by Epigenetics Flagship project CNR-Miur grants (to S.P.). This project has received funding from the European Research Council (ERC) under the European Union's Horizon 2020 Research and Innovation Program (Grant Agreement No. 670126-DENOVOSTEM).

Received: March 15, 2016
Revised: July 13, 2016
Accepted: August 12, 2016
Published: September 15, 2016

REFERENCES

Azzolin, L., Panciera, T., Soligo, S., Enzo, E., Bicciato, S., Dupont, S., Bresolin, S., Frasson, C., Basso, G., Guzzardo, V., et al. (2014). YAP/TAZ incorporation in the β-catenin destruction complex orchestrates the Wnt response. Cell *158*, 157–170.

Bar-Nur, O., Verheul, C., Sommer, A.G., Brumbaugh, J., Schwarz, B.A., Lipchina, I., Huebner, A.J., Mostoslavsky, G., and Hochedlinger, K. (2015). Lineage conversion induced by pluripotency factors involves transient passage through an iPSC stage. Nat. Biotechnol. *33*, 761–768.

Blanpain, C., and Fuchs, E. (2014). Stem cell plasticity. Plasticity of epithelial stem cells in tissue regeneration. Science *344*, 1242281.

Chakrabarti, R., Wei, Y., Hwang, J., Hang, X., Andres Blanco, M., Choudhury, A., Tiede, B., Romano, R.A., DeCoste, C., Mercatali, L., et al. (2014). DeltaNp63 promotes stem cell activity in mammary gland development and basal-like breast cancer by enhancing Fzd7 expression and Wnt signalling. Nat. Cell Biol. *16*, 1004–1015.

Cordenonsi, M., Zanconato, F., Azzolin, L., Forcato, M., Rosato, A., Frasson, C., Inui, M., Montagner, M., Parenti, A.R., Poletti, A., et al. (2011). The Hippo transducer TAZ confers cancer stem cell-related traits on breast cancer cells. Cell *147*, 759–772.

Dewachter, I., Reversé, D., Caluwaerts, N., Ris, L., Kuipéri, C., Van den Haute, C., Spittaels, K., Umans, L., Serneels, L., Thiry, E., et al. (2002). Neuronal deficiency of presenilin 1 inhibits amyloid plaque formation and corrects hippocampal long-term potentiation but not a cognitive defect of amyloid precursor protein [V717I] transgenic mice. J. Neurosci. *22*, 3445–3453.

Fitamant, J., Kottakis, F., Benhamouche, S., Tian, H.S., Chuvin, N., Parachoniak, C.A., Nagle, J.M., Perera, R.M., Lapouge, M., Deshpande, V., et al. (2015). YAP Inhibition Restores Hepatocyte Differentiation in Advanced HCC, Leading to Tumor Regression. Cell Rep. *10*, 1692–1707.

Guo, W., Keckesova, Z., Donaher, J.L., Shibue, T., Tischler, V., Reinhardt, F., Itzkovitz, S., Noske, A., Zürrer-Härdi, U., Bell, G., et al. (2012). Slug and Sox9 cooperatively determine the mammary stem cell state. Cell *148*, 1015–1028.

Han, X.J., Lu, Y.F., Li, S.A., Kaitsuka, T., Sato, Y., Tomizawa, K., Nairn, A.C., Takei, K., Matsui, H., and Matsushita, M. (2008). CaM kinase I alpha-induced phosphorylation of Drp1 regulates mitochondrial morphology. J. Cell Biol. *182*, 573–585.

Huch, M., Bonfanti, P., Boj, S.F., Sato, T., Loomans, C.J., van de Wetering, M., Sojoodi, M., Li, V.S., Schuijers, J., Gracanin, A., et al. (2013). Unlimited in vitro expansion of adult bi-potent pancreas progenitors through the Lgr5/R-spondin axis. EMBO J. *32*, 2708–2721.

Lee, D.H., Park, J.O., Kim, T.S., Kim, S.K., Kim, T.H., Kim, M.C., Park, G.S., Kim, J.H., Kuninaka, S., Olson, E.N., et al. (2016). LATS-YAP/TAZ controls lineage specification by regulating TGFbeta signaling and Hnf4alpha expression during liver development. Nat. Commun. *7*, 11961.

Means, A.L., Meszoely, I.M., Suzuki, K., Miyamoto, Y., Rustgi, A.K., Coffey, R.J., Jr., Wright, C.V., Stoffers, D.A., and Leach, S.D. (2005). Pancreatic epithelial plasticity mediated by acinar cell transdifferentiation and generation of nestin-positive intermediates. Development *132*, 3767–3776.

Pan, F.C., Bankaitis, E.D., Boyer, D., Xu, X., Van de Casteele, M., Magnuson, M.A., Heimberg, H., and Wright, C.V. (2013). Spatiotemporal patterns of multipotentiality in Ptf1a-expressing cells during pancreas organogenesis and injury-induced facultative restoration. Development *140*, 751–764.

Piccolo, S., Dupont, S., and Cordenonsi, M. (2014). The biology of YAP/TAZ: hippo signaling and beyond. Physiol. Rev. *94*, 1287–1312.

Plaks, V., Brenot, A., Lawson, D.A., Linnemann, J.R., Van Kappel, E.C., Wong, K.C., de Sauvage, F., Klein, O.D., and Werb, Z. (2013). Lgr5-expressing cells are sufficient and necessary for postnatal mammary gland organogenesis. Cell Rep. *3*, 70–78.

Sato, T., Vries, R.G., Snippert, H.J., van de Wetering, M., Barker, N., Stange, D.E., van Es, J.H., Abo, A., Kujala, P., Peters, P.J., and Clevers, H. (2009). Single Lgr5 stem cells build crypt-villus structures in vitro without a mesenchymal niche. Nature *459*, 262–265.

Stingl, J., Eirew, P., Ricketson, I., Shackleton, M., Vaillant, F., Choi, D., Li, H.I., and Eaves, C.J. (2006). Purification and unique properties of mammary epithelial stem cells. Nature *439*, 993–997.

Van Keymeulen, A., Rocha, A.S., Ousset, M., Beck, B., Bouvencourt, G., Rock, J., Sharma, N., Dekoninck, S., and Blanpain, C. (2011). Distinct stem cells contribute to mammary gland development and maintenance. Nature *479*, 189–193.

Wang, D., Cai, C., Dong, X., Yu, Q.C., Zhang, X.O., Yang, L., and Zeng, Y.A. (2015). Identification of multipotent mammary stem cells by protein C receptor expression. Nature *517*, 81–84.

Xu, J., Du, Y., and Deng, H. (2015). Direct lineage reprogramming: strategies, mechanisms, and applications. Cell Stem Cell *16*, 119–134.

Yimlamai, D., Christodoulou, C., Galli, G.G., Yanger, K., Pepe-Mooney, B., Gurung, B., Shrestha, K., Cahan, P., Stanger, B.Z., and Camargo, F.D. (2014). Hippo pathway activity influences liver cell fate. Cell *157*, 1324–1338.

Zanconato, F., Forcato, M., Battilana, G., Azzolin, L., Quaranta, E., Bodega, B., Rosato, A., Bicciato, S., Cordenonsi, M., and Piccolo, S. (2015). Genome-wide association between YAP/TAZ/TEAD and AP-1 at enhancers drives oncogenic growth. Nat. Cell Biol. *17*, 1218–1227.

Zanconato, F., Cordenonsi, M., and Piccolo, S. (2016). YAP/TAZ at the Roots of Cancer. Cancer Cell *29*, 783–803.

Zhu, Y., Romero, M.I., Ghosh, P., Ye, Z., Charnay, P., Rushing, E.J., Marth, J.D., and Parada, L.F. (2001). Ablation of NF1 function in neurons induces abnormal development of cerebral cortex and reactive gliosis in the brain. Genes Dev. *15*, 859–876.

Cell Stem Cell, Volume *19*

Supplemental Information

Induction of Expandable Tissue-Specific Stem/Progenitor Cells through Transient Expression of YAP/TAZ

Tito Panciera, Luca Azzolin, Atsushi Fujimura, Daniele Di Biagio, Chiara Frasson, Silvia Bresolin, Sandra Soligo, Giuseppe Basso, Silvio Bicciato, Antonio Rosato, Michelangelo Cordenonsi, and Stefano Piccolo

THIS SECTION CONTAINS:

- SUPPLEMENTAL FIGURES 1-6 AND LEGENDS
- SUPPLEMENTAL MOVIE LEGENDS
- EXTENDED EXPERIMENTAL PROCEDURES
- SUPPLEMENTAL REFERENCES

SUPPLEMENTAL FIGURES AND LEGENDS

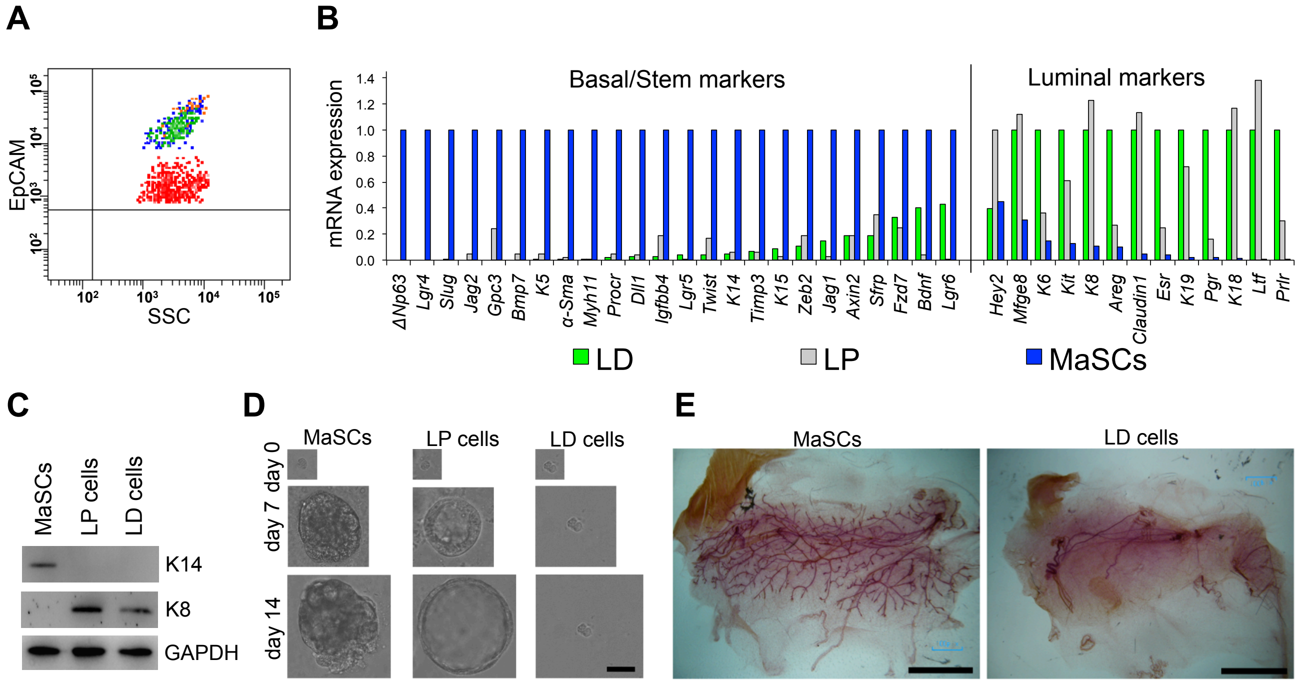

F

1 plated cell in 1 single well; visually verified	Experiment			Mean ± SD
	#1	#2	#3	
**LD: rtTA/YAP wt transduced**				
# of single LD cells	n = 71	n = 68	n = 57	
% of single cells that gave rise to a single LP-like colony	0%	0%	0%	0
% of single cells that gave rise to a single MaSC-like colony	19.7%	14.7%	21.1%	18.5 ± 3.4
**LD: rtTA/EGFP transduced** (negative control)				
# of single LD cells	n = 56	n = 64	n = 70	
% of single cells that gave rise to a single LP-like colony	0%	0%	0%	0
% of single cells that gave rise to a single MaSC-like colony	0%	0%	0%	0
**LD: rtTA/YAP S94A transduced** (transcriptionally inactive, negative control)				
# of single LD cells	n = 73	n = 46	n = 50	
% of single cells that gave rise to a single LP-like colony	0%	0%	0%	0
% of single cells that gave rise to a single MaSC-like colony	0%	0%	0%	0

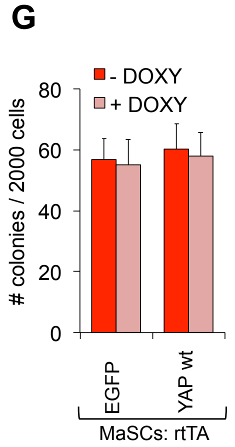

Figure S1

Figure S1. Characterization of FACS-sorted mammary cells and induction of MaSC-like traits in luminal differentiated cells by YAP/TAZ. Related to Figure 1.

A, FACS profile for EpCAM of the experiments represented in Figure 1A.

B-C, qRT-PCRs and western blots for the indicated basal/stem and luminal markers in MaSCs, LP, and LD cells obtained by FACS, as in Figure 1A. In **B**, data are normalized to *Gapdh* expression and are referred to MaSC levels for basal genes, to LP levels for *Hey1*, and to LD levels for all the other luminal markers (each set to 1). Results are representative of at least three independent experiments (each using mammary glands from n=20 mice) performed in triplicate. In **C**, GAPDH serves as loading control.

D, Representative images of mammary colonies formed by the indicated cells, growing at the indicated time points in mammary colony medium. MaSCs formed solid outgrowths, while LD remained as single cells. LP cells, despite being able to form acinar (cavitated) colonies, were unable to self-renew after passaging, or to form organoids when transferred in 100% Matrigel/mammary organoid medium culture system (not shown). Pictures are representative of three independent experiments performed with six technical replicates. Scale bar, 170 μm.

E, Representative images of whole mount hematoxylin staining of cleared fat pads injected with purified MaSCs (leading to outgrowth of a ductal mammary tree) or of LD cells as negative control. LP cells were similarly void of regenerative potential in vivo (not shown). Scale bars, 1 cm.

F, Detailed quantification of the conversion rates of single, visually inspected, LD cells, expressing the indicated constructs and plated in 96-well plates, into a MaSC-like state as determined by % of colony formation. EGFP- and YAP S94A-transduced cells serve as negative control. For quantification of the lentiviral infection and of the organoid-forming efficiency of yMaSCs colonies once transferred in organoid medium, see Methods.

G, Quantifications of mammary colonies formed by the indicated cells, 15 days after seeding in mammary colony medium. Data are presented as mean + s.d.

A

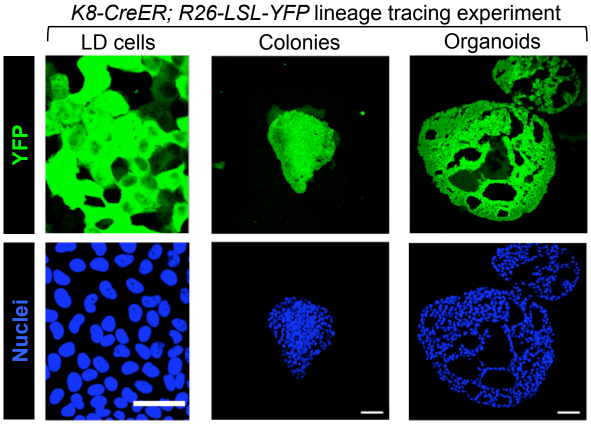
K8-CreER; R26-LSL-YFP lineage tracing experiment
LD cells
Colonies
Organoids
YFP
Nuclei

Figure S2

Figure S2. Lineage tracing of yMaSC-derived colonies and organoids.

Related to Figure 2.

A, anti-YFP immunostaining of the lineage tracing experiment showing that yMaSC-derived colonies and organoids originate from tamoxifen-treated *K8-CreERT2; R26-LSL-YFP* LD cells. Scale bars, 49 μm.

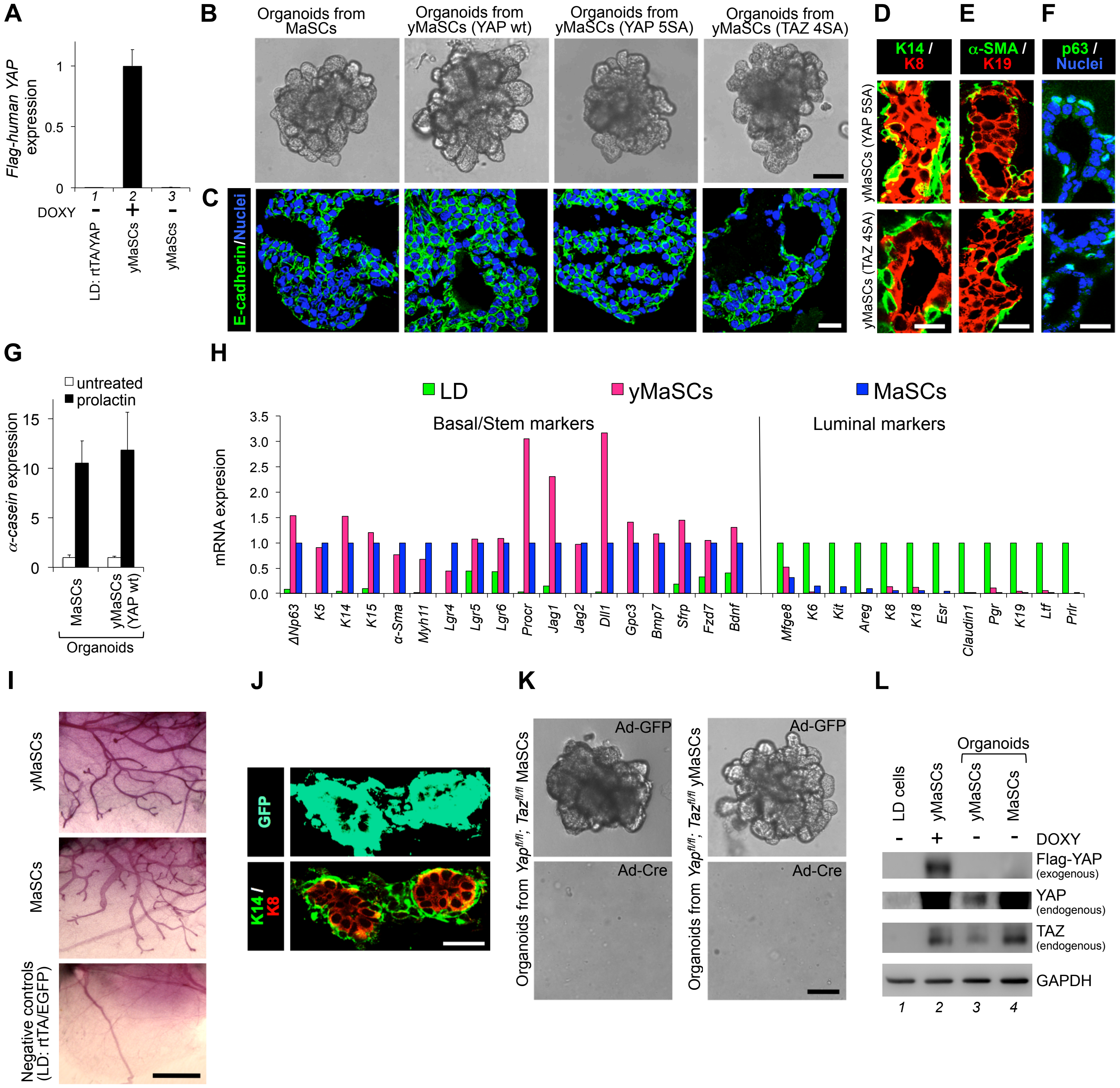
A
Flag-human YAP expression
DOXY
LD: rtTA/YAP
yMaSCs
yMaSCs
B
Organoids from MaSCs
Organoids from yMaSCs (YAP wt)
Organoids from yMaSCs (YAP 5SA)
Organoids from yMaSCs (TAZ 4SA)
C
E-cadherin/Nuclei
D
K14 / K8
E
α-SMA / K19
F
p63 / Nuclei
yMaSCs (YAP 5SA)
yMaSCs (TAZ 4SA)
G
untreated
prolactin
α-casein expression
MaSCs
yMaSCs (YAP wt)
Organoids
H
LD
yMaSCs
MaSCs
Basal/Stem markers
Luminal markers
mRNA expresion
ΔNp63
K5
K14
K15
α-Sma
Myh11
Lgr4
Lgr5
Lgr6
Procr
Jag1
Jag2
Dll1
Gpc3
Bmp7
Sfrp
Fzd7
Bdnf
Mfge8
K6
Kit
Areg
K8
K18
Esr
Claudin1
Pgr
K19
Ltf
Prlr
I
yMaSCs
MaSCs
Negative controls (LD: rtTA/EGFP)
J
GFP
K14 / K8
K
Organoids from Yapfl/fl; Tazfl/fl MaSCs
Organoids from Yapfl/fl; Tazfl/fl yMaSCs
Ad-GFP
Ad-Cre
L
Organoids
LD cells
yMaSCs
yMaSCs
MaSCs
DOXY
Flag-YAP (exogenous)
YAP (endogenous)
TAZ (endogenous)
GAPDH

Figure S3

Figure S3. Characterization of mammary organoids derived from MaSCs and yMaSCS. Related to Figure 3.

A, qRT-PCRs for transgenic Flag-human YAP in the indicated samples. Samples are: LD cells infected with rtTA and tetO-YAP lentiviral particles and either left untreated (lane 1, NO Doxy) or cultured in the presence of doxycycline to induce yMaSCs (lane 2). Next, yMaSCs were maintained in Matrigel as organoids (see Methods and Figure 1D) in the absence of doxycycline for 5 months (lane 3, NO Doxy). Data are normalized to *Gapdh* expression and are presented as mean + s.d. of two independent replicates. Note that yMaSCs kept in absence of doxycycline do not express the transgene.

B, Representative images of MaSCs or yMaSCs organoids (derived from YAPwt, YAP5SA or TAZ4SA, as indicated); see Figure 1D for a diagram of the experimental plan. Scale bar, 250 μm.

C, Organoids from MaSCs (positive control) and the indicated yMaSCs expressed E-cadherin by confocal immunofluorescence on frozen sections. Scale bar, 18 μm.

D-F, Organoids from the indicated yMaSCs expressed basal/stem (K14, α-SMA, p63) and luminal (K8, K19) markers by confocal immunofluorescence on frozen sections. Scale bars, 17 μm.

G, Compendium of Figure 3F. Treatment with prolactin triggers *α-casein* expression in MaSC- and yMaSC-derived organoids, as monitored by qRT-PCR. Data are normalized to *Gapdh* expression. Untreated samples were set to 1. Results are representative of two independent experiments, each performed in triplicate. Data are mean + s.d.

H, Comparison by qRT-PCR of FACS-purified MaSC-enriched basal cells (MaSCs) from the mammary gland and FACS-purified yMaSCs from yMaSC-derived organoids. Purified LD cells were used as control. Data are normalized to *Gapdh* expression and are referred to MaSCs levels for basal genes and to LD levels for all the luminal markers (each set to 1).

I, Representative images of whole-mount hematoxylin staining of cleared fat pad with reconstituted mammary trees from transplanted yMaSCs (from wtYAP), native MaSCs (positive control) and rtTA/EGFP control LD cells (negative control). Scale bar, 0.5 cm.

J, This refers to Figure 3J of the Main Text. Representative sections of virgin mammary gland tree derived from injected MaSCs stained for GFP, K14 and K8. Scale bar, 21 μm.

K, Organoids were obtained from *Yap*$^{fl/fl}$*; Taz*$^{fl/fl}$ MaSCs and yMaSCs. During passaging at the single cell level, they were transduced with Ad-Cre or Ad-GFP as control. Panels are representative images of the resulting outgrowths. No organoid ever formed in absence of YAP/TAZ. Scale bar, 250 μm.

L, Panels are western blots for YAP and TAZ of lysates from the indicated cells. Lane 1: FACS-sorted LD cells. Lane 2: yMaSCs (wtYAP) after seven days of doxycycline treatment (as in Figure 1D); tagged Flag-hYAP (with a higher molecular weight than endogenous mouse YAP) is induced. Lane 3: organoids from yMaSCs cultured in the absence of doxycycline (Flag-hYAP turned off, but endogenous YAP/TAZ remain expressed). Lane 4: control of endogenous MaSCs. GAPDH serves as loading control.

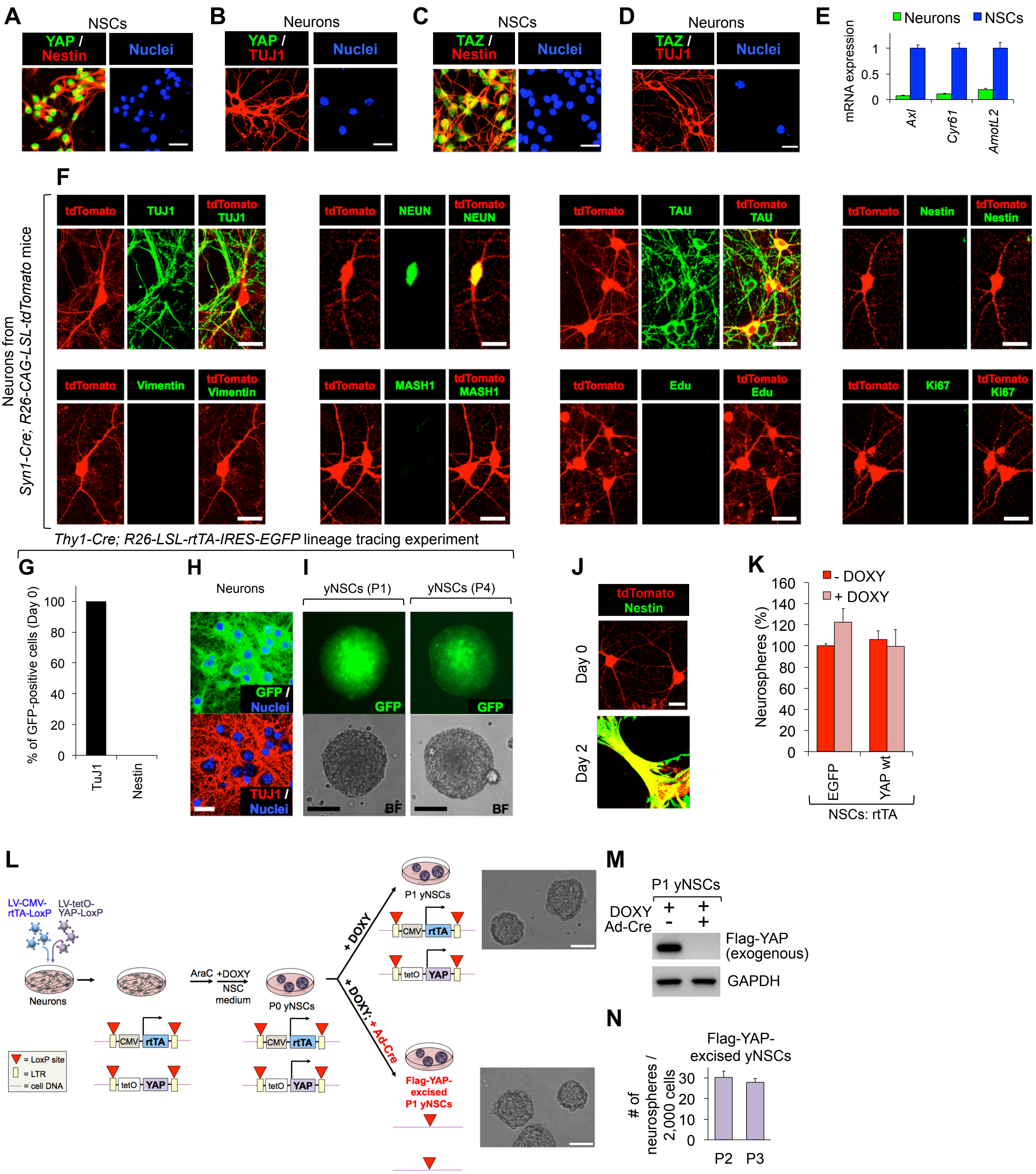
A
NSCs
YAP / Nestin
Nuclei
B
Neurons
YAP / TUJ1
Nuclei
C
NSCs
TAZ / Nestin
Nuclei
D
Neurons
TAZ / TUJ1
Nuclei
E
Neurons
NSCs
mRNA expression
Axl
Cyr61
AmotL2
F
Neurons from Syn1-Cre; R26-CAG-LSL-tdTomato mice
tdTomato
TUJ1
NEUN
TAU
Nestin
Vimentin
MASH1
Edu
Ki67
Thy1-Cre; R26-LSL-rtTA-IRES-EGFP lineage tracing experiment
G
% of GFP-positive cells (Day 0)
TuJ1
Nestin
H
Neurons
GFP / Nuclei
TUJ1 / Nuclei
I
yNSCs (P1)
yNSCs (P4)
GFP
BF
J
tdTomato Nestin
Day 0
Day 2
K
- DOXY
+ DOXY
Neurospheres (%)
EGFP
YAP wt
NSCs: rtTA
L
LV-CMV-rtTA-LoxP
LV-tetO-YAP-LoxP
Neurons
AraC +DOXY NSC medium
P0 yNSCs
+ DOXY
P1 yNSCs
+ DOXY; + Ad-Cre
Flag-YAP-excised P1 yNSCs
CMV
rtTA
tetO
YAP
= LoxP site
= LTR
= cell DNA
M
P1 yNSCs
DOXY
Ad-Cre
Flag-YAP (exogenous)
GAPDH
N
Flag-YAP-excised yNSCs
# of neurospheres / 2,000 cells
P2
P3

Figure S4

Figure S4. Expression of YAP/TAZ in neurons and lineage tracing experiments showing that yNSCs originate from neurons. Related to Figure 4.

A-B, Representative confocal images of NSCs (plated as monolayer) and hippocampal neurons, costained for YAP/Nestin and YAP/TUJ1, respectively. Nuclei were stained with DAPI. Scale bars, 23 μm.

C-D, Representative confocal images of endogenous TAZ costained with Nestin in primary NSCs (**C**) or with TUJ1 in primary hippocampal neurons (**D**). Nuclei were stained with DAPI. Scale bars, 23 μm.

E, qRT-PCRs for the known YAP/TAZ targets genes *Axl, Cyr61 and AmotL2* in hippocampal neurons and NSCs (mean + s.d.). Results are representative of three independent experiments performed in triplicate. Data were normalized to *Gapdh* expression.

F, tdTomato fluorescence and immunostaining for the indicated markers in cortical neurons obtained from *Syn1-Cre; R26-CAG-LSL-tdTomato* mice. Scale bars, 19 μm.

G-I, Lineage tracing experiment with the *Thy1-Cre* driver showing that yNSCs originate from neurons. **G**, quantification of the percentage of EGFP-positive cells (Thy1-traced) expressing TUJ1 or Nestin (n=200). **H**, immunostaining for GFP and TUJ1 in hippocampal neurons obtained from *Thy1-Cre; R26-LSL-rtTA-IRES-EGFP* mice. **I**, bright field and GFP-fluorescence pictures of yNSC-generated neurospheres obtained from neurons as in (G, H) after reprogramming using doxycycline-inducible YAP. Scale bars in **H**, 37 μm, in **I**, 105 μm.

J, Immunofluorescences for Nestin during the conversion of Syn1-Cre/tdTomato-traced YAP-expressing cortical neurons (day 0) to yNSCs (day 2).

K, Quantifications of neurospheres formed by the indicated cells, 7 days after seeding suspension cultures in NSC medium. Data are presented as mean + s.d.

L-N, Hippocampal neurons were transduced with the Cre-excisable vectors encoding for rtTA and doxycycline-inducible Flag-human wild-type YAP, and treated to obtain P0 yNSCs. P0 yNSCs were dissociated at the single cell level and replated in NSC medium + doxycycline to allow P1 yNSCs formation with or without Ad-Cre, that enables the excision of the whole viral integrated cassette. **L,** the panel includes representative images of the yNSCs, before and post-excision. Scale bar, 210 μm. **M**, Flag-human YAP could not be detected post-excision. GAPDH serves as loading control. **N**, quantification of

neurospheres from yNSCs post-excision in two serial passages. Results are representative of two independent experiments, each performed in six replicates. Data are mean + s.d.

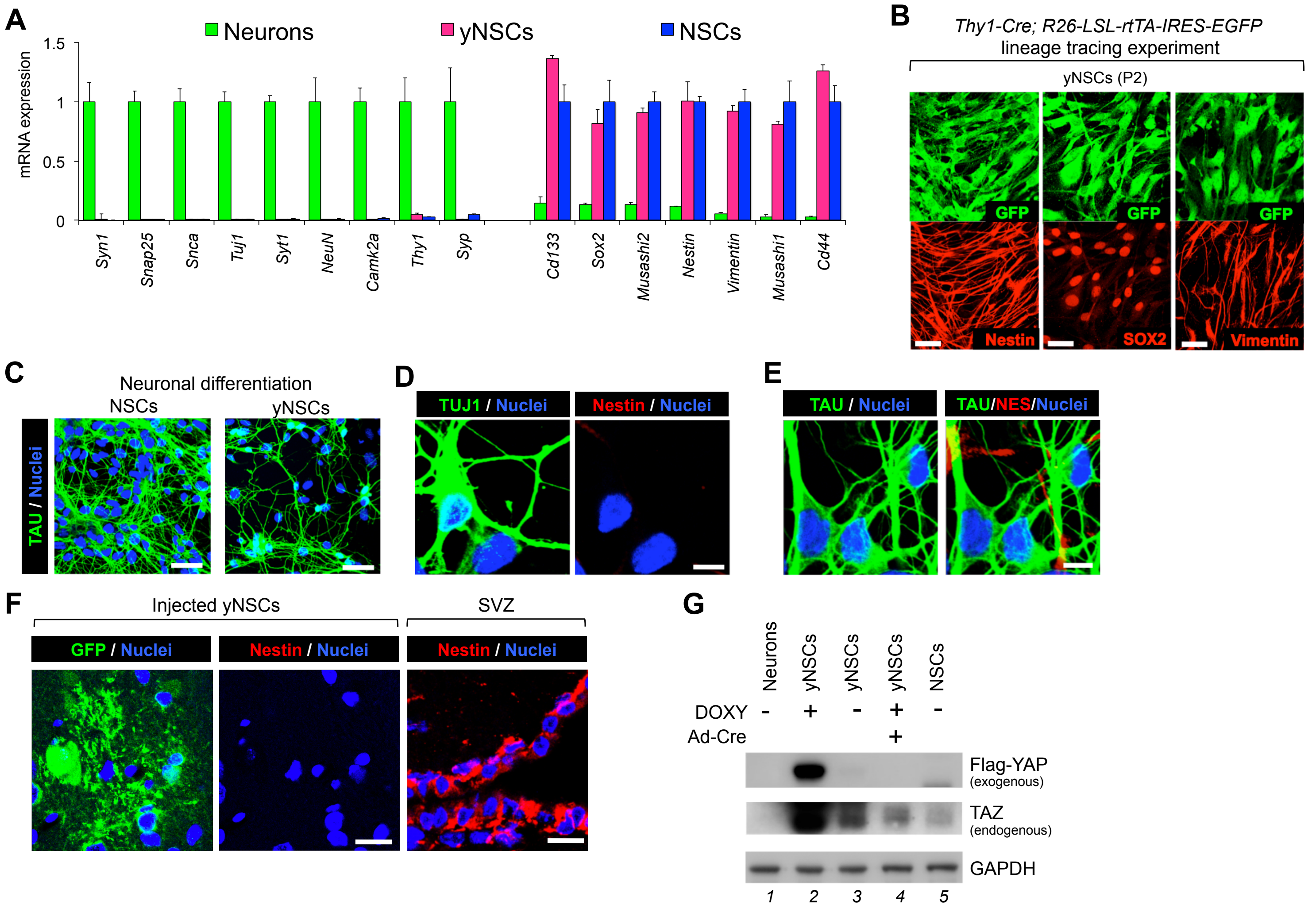

A
Neurons
yNSCs
NSCs
mRNA expression
1.5
1
0.5
0
Syn1
Snap25
Snca
Tuj1
Syt1
NeuN
Camk2a
Thy1
Syp
Cd133
Sox2
Musashi2
Nestin
Vimentin
Musashi1
Cd44
B
Thy1-Cre; R26-LSL-rtTA-IRES-EGFP
lineage tracing experiment
yNSCs (P2)
GFP
GFP
GFP
Nestin
SOX2
Vimentin
C
Neuronal differentiation
NSCs
yNSCs
TAU / Nuclei
D
TUJ1 / Nuclei
Nestin / Nuclei
E
TAU / Nuclei
TAU/NES/Nuclei
F
Injected yNSCs
SVZ
GFP / Nuclei
Nestin / Nuclei
Nestin / Nuclei
G
Neurons
yNSCs
yNSCs
yNSCs
NSCs
DOXY - + - + -
Ad-Cre +
Flag-YAP
(exogenous)
TAZ
(endogenous)
GAPDH
1 2 3 4 5

Figure S5

Figure S5. Molecular characterization of yNSCs. Related to Figure 5.

A, qRT-PCRs for the indicated neuronal and neural stem cell markers in cortical neurons, yNSCs and NSCs. Data are normalized to *Gapdh* expression and are referred to neurons for neuronal genes, and to NSCs for neural stem cell markers (each set to 1). Results are representative of at least three independent experiments performed in triplicate.

B, Immunostainings of yNSCs derived as in Figure S4G-I plated in 2D, showing positivity for GFP and neural stem cell markers Nestin, SOX2 and Vimentin. Scale bars, 37μm.

C, This refers to Figure 5E. yNSCs and NSCs were plated and differentiated toward a neuronal fate (see Methods). Panels represent confocal images for neuronal differentiation marker TAU. Scale bars, 50 μm.

D-E, These refer to Figure 5E of the main text and to Figure S5C. By immunofluorescence, neurons differentiated from yNSCs are TUJ1-positive (D) or TAU-positive (E) but are negative for Nestin. Scale bars, 9μm.

F, Panels are representative confocal images showing that injected cells (GFP-positive) lost expression of the NSC marker Nestin. A field of the subventricular zone (SVZ) of the same brain sections is shown as positive control of the anti-Nestin antibody staining.

G, Panels are western blots for YAP and TAZ from protein extracts of the indicated cells. Lane 1: neurons. Lane 2: yNSCs (P0) were obtained using excisable YAP transgene, and maintained in Doxy; as in Figure S4M-N. Cells (from P2-to-P3) were plated as monolayer in presence of Doxy and lysed after 1 day. Lane 3: the same yNSCs of lane 2, kept in absence of Doxy from P2. Lane 4: yNSCs as in lane 2, but after excision of the viral cassette (at P1, as Figure S4L-M). Lane 5: lysates of NSCs as comparison.

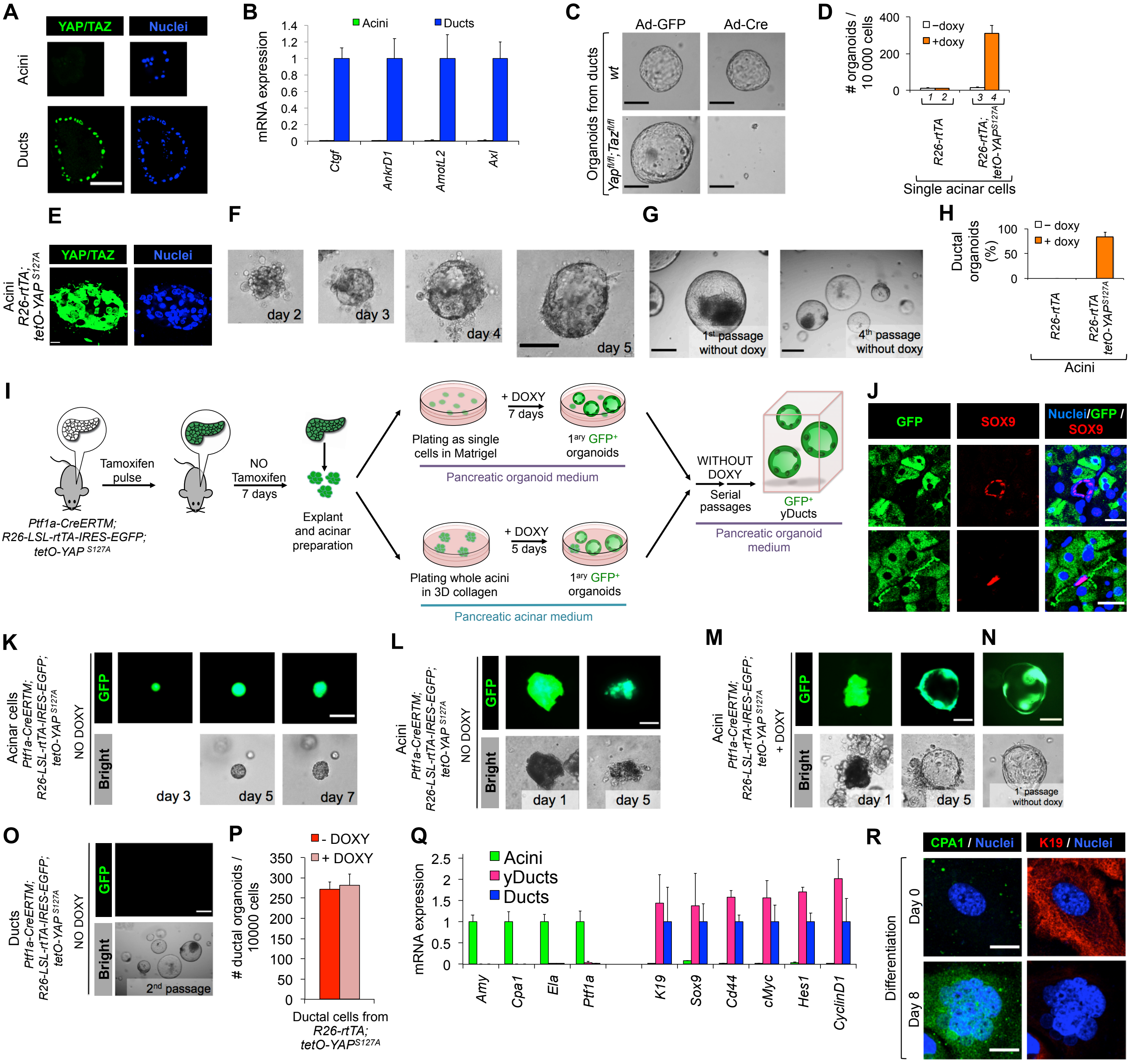
A
YAP/TAZ
Nuclei
Acini
Ducts
B
Acini
Ducts
mRNA expression
Ctgf
AnkrD1
AmotL2
Axl
C
Ad-GFP
Ad-Cre
Organoids from ducts
wt
D
organoids / 10 000 cells
−doxy
+doxy
R26-rtTA
R26-rtTA; tetO-YAP S127A
Single acinar cells
E
Acini R26-rtTA; tetO-YAP S127A
F
day 2
day 3
day 4
day 5
G
1st passage without doxy
4th passage without doxy
H
Ductal organoids (%)
− doxy
+ doxy
Acini
I
Tamoxifen pulse
NO Tamoxifen 7 days
Explant and acinar preparation
Ptf1a-CreERTM; R26-LSL-rtTA-IRES-EGFP; tetO-YAP S127A
Plating as single cells in Matrigel
+ DOXY 7 days
1ary GFP+ organoids
Pancreatic organoid medium
Plating whole acini in 3D collagen
+ DOXY 5 days
Pancreatic acinar medium
WITHOUT DOXY
Serial passages
GFP+ yDucts
Pancreatic organoid medium
J
GFP
SOX9
Nuclei/GFP / SOX9
K
Acinar cells Ptf1a-CreERTM; R26-LSL-rtTA-IRES-EGFP; tetO-YAP S127A
NO DOXY
GFP
Bright
day 3
day 5
day 7
L
Acini Ptf1a-CreERTM; R26-LSL-rtTA-IRES-EGFP; tetO-YAP S127A
day 1
M
+ DOXY
N
passage without doxy
O
Ducts Ptf1a-CreERTM; R26-LSL-rtTA-IRES-EGFP; tetO-YAP S127A
2nd passage
P
- DOXY
+ DOXY
# ductal organoids / 10000 cells
Ductal cells from R26-rtTA; tetO-YAP S127A
Q
Acini
yDucts
Ducts
Amy
Cpa1
Ela
Ptf1a
K19
Sox9
Cd44
cMyc
Hes1
CyclinD1
R
CPA1 / Nuclei
K19 / Nuclei
Differentiation
Day 0
Day 8

Figure S6

Figure S6. YAP expression converts pancreatic acinar explants to duct-like organoids.

Related to Figure 6.

A, Pancreatic ductal organoids ("Ducts", bottom panels) display nuclear YAP/TAZ by immunofluorescence. YAP/TAZ are not detected in pancreatic acini (top panels). Scale bar, 80μm.

B, qRT-PCRs for the known YAP/TAZ targets genes *Axl, Ctgf, Amotl2 and AnkrD1* in primary pancreatic acini and pancreatic ductal organoids (mean + s.d). Results are representative of three independent experiments performed in triplicate. Data were normalized to *18S rRNA* expression.

C, Ducts were derived from wild-type (*wt*) or $Yap^{fl/fl}$; $Taz^{fl/fl}$ mice and, during passaging at the single cell level, transduced with Ad-Cre or Ad-GFP as control. Panels are representative images of resulting outgrowths. Scale bars, 70 μm.

D, This refers to Figure 6C of the main text. Quantification of primary organoids arising from *R26-rtTA; tetO-*YAP^{S127A} single acinar cells. Lanes 1-3 are negative controls; although rare colonies were still counted in these control wells, at difference of bona fide organoids, in no case these could be amplified in subsequent passages. >90% of the YAP-induced yDucts in lane 4 were large and well-cavitated cysts, as those shown in Figure 6C, that were never found in controls. Data are presented as mean + s.d. and are representative of five independent experiments, performed with four technical replicates.

E, Representative immunostaining for YAP/TAZ showing control of the induction of transgenic YAP in pancreatic acini derived from *R26-rtTA; tetO-*YAP^{S127A} 5 days after Doxycycline addition. Scale bar, 24 μm.

F-G, Serial images of a whole acinus derived from *R26-rtTA; tetO-*YAP^{S127A} growing as cyst-like organoid at the indicated time points after Doxycycline addition (**F**) and after one or four passages in fresh Matrigel in the absence of Doxycycline (**G**). Scale bars, 70 μm in **F**; 290 μm in **G**. See also Movies S1 and S2.

H, Quantification of the ability of whole acini to form ductal organoids upon transgenic YAP overexpression as in Figure S6F. Data are presented as mean + s.d. and are representative of five independent experiments, performed with four technical replicates.

I, Schematic representation of the experiments performed with pancreatic acinar explants for lineage tracing. Pancreatic acini were isolated from *Ptf1a-CreERTM; R26-LSL-rtTA-IRES-EGFP; tetO-*YAP^{S127A} mice and either seeded as single cells in Matrigel or as whole acini in collagen (see Methods). Acinar cells were cultured in the presence of doxycycline

until primary organoids appeared. Organoids obtained from both culture conditions were then passaged in fresh Matrigel in the absence of doxycycline every 10 days.

J, Representative immunofluorescence pictures, showing that, 1 week after tamoxifen induction, *Ptf1a-CreERTM; R26-LSL-rtTA-IRES-EGFP* lineage tracer is labeling acinar cells, but not SOX9-positive ductal (top panels) or SOX9-positive centroacinar (bottom panels) cells. Scale bars, 19 µm.

K, This is a negative control that refers to Figure 6E. Panels are bright field and GFP-fluorescence pictures of transgenic *Ptf1a-CreERTM; R26-LSL-rtTA-IRES-EGFP; tetO-YAP*^{S127A} exocrine cells, at the indicated time points in absence of Doxy treatment. Scale bar, 33µm.

L, Negative control of the experiment shown in Figure S6M,N. Panels are bright field and GFP-fluorescence pictures of *Ptf1a-CreERTM; R26-LSL-rtTA-IRES-EGFP; tetO-YAP*^{S127A} whole exocrine acini, at the indicated time points in absence of Doxy treatment (Negative controls). Scale bar, 33µm

M-N, Lineage-tracing experiments using the *Ptf1a-CreERTM* driver. Panels are bright field and GFP-fluorescence pictures of transgenic YAP-expressing whole exocrine acini derived from *Ptf1a-CreERTM; R26-LSL-rtTA-IRES-EGFP; tetO-YAP*^{S127A} mice, at the indicated time points of Doxy treatment (**M**) and after passaging in absence of Doxy in fresh Matrigel (**N**). Scale bars, 33 µm in **M**; 70 µm in **N.** See also Movie S3.

O, Panels are bright field and GFP-fluorescence pictures of *Ptf1a-CreERTM; R26-LSL-rtTA-IRES-EGFP; tetO-YAP*^{S127A} ductal progenitors, at the indicated passage of organoid culture. Scale bar, 290 µm.

P, Quantifications of ductal organoids formed by the indicated cells 7 days after seeding in pancreatic organoid medium. Data are presented as mean + s.d.

Q, qRT-PCRs for the indicated exocrine and Ductal/progenitor markers in fresh pancreatic acini, yDucts and Ducts. Data are normalized to *18S* rRNA expression and are referred to Acini for exocrine differentiation markers, and to Ducts for Ductal/progenitor genes (each set to 1). Results are representative of four independent experiments performed in triplicate. Data are presented as mean + s.d.

R, Representative immunofluorescences for the ductal marker K19 and the exocrine marker CPA1 before (day 0) and after yDuct differentiation (day 8). Similar results were obtained with organoids from normal ducts (not shown). Scale bars:19 µm (day 0), 16 µm (day8).

SUPPLEMENTAL MOVIE LEGENDS

Movie S1. Time-lapse movie showing conversion of acini into yDucts. Related to Figure 6.

The movie shows the same field of whole pancreatic acini derived from *R26-rtTA; tetO-YAP*^{S127A} mice during their conversion into cyst-like organoids at the indicated time points (hours) after doxycycline addition.

Movie S2. Time-lapse movie showing conversion of a single acinus into yDuct. Related to Figure 6.

The movie shows a single pancreatic acinus derived from *R26-rtTA; tetO-YAP*^{S127A} mice during conversion into cyst-like organoid at the indicated time points (hours) after doxycycline addition.

Movie S3. Time-lapse movie showing conversion of a lineage traced- single acinus into yDuct. Related to Figure 6.

Bright-field and GFP-fluorescence movie showing a single pancreatic acinus derived from *Ptf1a-CreERTM; R26-LSL-rtTA-IRES-EGFP; tetO-YAP*^{S127A} mice during conversion into cyst-like organoid at the indicated time points (hours) after doxycycline addition.

EXTENDED EXPERIMENTAL PROCEDURES

Reagents, plasmids and transfections

Doxycycline hyclate, fibronectin, collagen I, heparin, insulin, dexamethasone, SBTI (Soybean Trypsin Inhibitor), gastrin, N-acethylcysteine, nicotinamide, T3 (Triiodo-L-Thyronine), tamoxifen and 4-OH-tamoxifen were from Sigma. Murine EGF, murine bFGF, human FGF10, human Noggin, human IGF, murine prolactin and BMP4 were from Peprotech. N2, B27 (always containing vitamin A), BPE and ITS-X (Insulin-Transferrin-Selenium-Ethanolamine) supplements were from Life Technologies. R-Spondin1 was from Sino Biological. Matrigel was from BD Biosciences (Corning). Rat tail collagen type I was from Cultrex.

GFP- and Cre-expressing adenoviruses were from University of Iowa, Gene Transfer Vector Core. For inducible expression of YAP and TAZ, cDNA for siRNA-insensitive Flag-hYAP1 wt, S94A (TEAD-binding mutant(Zhao et al., 2008)) and 5SA (LATS-mutant sites(Aragona et al., 2013)) and for Flag-mTAZ4SA(Azzolin et al., 2012) were subcloned in FUW-tetO-MCS, obtained by substituting the Oct4 sequence in FUW-tetO-hOct4 (Addgene #20726(Hockemeyer et al., 2008)) with a new multiple cloning site (MCS). This generated the FUW-tetO-wtYAP, FUW-tetO-YAPS94A, FUW-tetO-YAP5SA, FUW-tetO-TAZ4SA used throughout this study. FUW-tetO-MCS (empty vector) or FUW-tetO-EGFP plasmids were used as controls, as previously indicated(Cordenonsi et al., 2011).

For stable expression of GFP, we used pRRLSIN.cPPT.PGK-GFP.WPRE (gift of L. Naldini) lentiviral vector.

For Cre-excisable expression of rtTA, we used LV-CMV-rtTA-LoxP (Figures S4L-N), obtained by substituting the Cre cDNA in LV-CMV-Cre-LoxP with the cDNA of rtTA from FUdeltaGW-rtTA (Addgene #19780(Maherali et al., 2008)).

For Cre-excisable lentiviral vector containing the tetO-Flag-hYAP wt cassette, we used LV-tetO-YAP wt-LoxP (Figures S4L-N), obtained by substituting the CMV-Cre cassette in LV-CMV-Cre-LoxP with the tetO-Flag-hYAP wt cassette from FUW-tetO-Flag-hYAP1 wt.

All constructs were confirmed by sequencing.

siRNA transfections were done with Lipofectamine RNAi-MAX (Life technologies) in antibiotics-free medium according to manufacturer instructions. Sequences of siRNAs targeting murine *Yap* and *Taz* were as previously decribed (Azzolin et al., 2014).

DNA transfections were done with TransitLT1 (Mirus Bio) according to manufacturer instructions.

Lentiviral preparation

HEK293T cells (checked routinely for absence of mycoplasma contaminations) were kept in DMEM supplemented with 10% FBS (Life Technologies), Glutamine and Antibiotics (HEK medium). Lentiviral particles were prepared by transiently transfecting HEK293T with lentiviral vectors (10 micrograms/10 cm dishes) together with packaging vectors pMD2-VSVG (2.5 micrograms) and pPAX2 (7.5 micrograms) by using TransIT-LT1 (Mirus Bio) according to manufacturer instructions. Specifically, 60 μl of TransIT-LT1 reagent was diluted in 1.5 ml of Opti-MEM (Life Technologies) for each 10 cm dish, incubated with the vector DNA 15 min at RT and gently distributed over to the cell medium (dish contained about 10 ml of HEK medium). After 8 hr, HEK medium was changed. 48 hr post-transfection supernatant was collected, filtered through 0.45 micrometers and directly stored at -20°C; we did not concentrate viral supernatants. We also seldom measured the lentiviral titer of various supernatants used in our experiments using the QuickTiter Lentivirus Titer kit (lentivirus-associated HIV p24; Cell Biolabs) according to the manufacturer's protocol. As reference, viral supernatants were in the range 16 - 20 ng of p24/ml. This titer corresponds to a viral particle concentration of about $2x10^8$ particles/ml. As determined by PCR of integrated lentiviral DNA of HEK293T transduced with pRRL-EGFP, this roughly corresponds to $2x10^6$ transduction units (TU)/ml in the unconcentrated viral supernatant stock.

Lentiviral Infection of primary cells

As example, for a typical infection of a 3 cm dish containing primary cells, we used 500 μl of each unconcentrated viral supernatant diluted to 1x cell-specific medium in 2 ml final volume. For mammary gland cells, we mixed one volume (e.g., 500 μl/3.5 cm dish) of FUdeltaGW-rtTA supernatant, one volume of the FUW-tetO-YAP (or TAZ) supernatant, and added two volumes of serum-free MG medium with 2X concentrations of supplements. For neurons, for a 3.5 cm well, we mixed 500 μl of FUdeltaGW-rtTA with 250 μl of FUW-tetO-wtYAP (or negative controls) viral supernatants, 250 μl of HEK medium with 1.5 ml of serum-free Neurobasal medium with 2X B27.

Mammary gland dissociation

Primary MECs were isolated from the mammary glands of 8- to 12-week-old virgin C57BL/6J mice (unless otherwise specified), according to standard procedures (Stingl et

al., 2006). Mammary glands were minced and then digested with 6000 U/ml collagenase I (Life Technologies) and 2000 U/ml hyaluronidase (Sigma) in the DMEM/F12 (Life Technologies) at 37°C for 1 hour with vigorous shaking. The digested samples were pipetted, spun down at 1500 rpm for 5 min, and incubated 3 min in 0.64% buffered NH_4Cl (Sigma) in order to eliminate contaminating red blood cells. After washing with DMEM/F12 + 5% FBS, cells were plated for 1 hour at 37°C in DMEM/F12+5% FBS: in this way, the majority of fibroblasts attached to the tissue culture plastic, whereas mammary epithelial populations did not; MEC were thus recovered in the supernatant and pelleted. After washing in PBS/EDTA 0.02%, MECs were further digested with 0.25% trypsin (Life Technologies) for 5 min and 5 mg/ml dispase (Sigma) plus 100 μg/ml DNase I (Roche) for other 10 min. The digested cells were diluted in DMEM/F12+5%FBS and filtered through 40 μm cell strainers to obtain single cell suspensions cells and washed once in the same medium.

Matrigel culture of mammary colonies and organoids

After infection in 2D cultures and induction with doxycycline for 7 days, mammary cells were detached with trypsin and seeded at a density of 1,000 cells/well in 24-well ultralow attachment plates (Corning) in mammary colony medium (DMEM/F12 containing glutamine, antibiotics, 5% Matrigel, 5% FBS, 10 ng/ml murine EGF, 20 ng/ml murine bFGF, and 4 μg/ml heparin) containing doxycycline (2 μg/ml). Primary colonies were counted 14 days after seeding. To show the self-renewal capacity of yMaSCs independently of exogenous YAP/TAZ supply (i.e, independently of doxycycline administration), primary colonies were recovered from the MG-colony medium by collecting the samples and incubation with an excess volume of ice cold HBSS in order to solubilize Matrigel. After 1 hour, colonies were rinsed 3 times in cold HBSS by centrifugation at 1000 rpm for 5 min and incubated in trypsin 0.05% for 30 min to obtain a single cell suspension. Cells were counted and re-seeded at 1,000 cells/well in 24-well ultralow attachment plates in MG colony medium without doxycycline for further passaging.

For mammary organoid formation, primary colonies were recovered from MG colony medium in cold HBSS and transferred in 100% Matrigel. After Matrigel formed a gel at 37°C, MG organoid medium was added (Advanced DMEM/F12 supplemented with Hepes, GlutaMax, antibiotics, B27 1X, 50 ng/ml murine EGF, 20 ng/ml murine bFGF, 4 μg/ml heparin, 100 ng/ml Noggin and 1 μg/ml R-Spondin1). Note that at this step we do not

dissociate at single cell level the primary colonies but simply transfer them to organoid culture conditions. After few days, colonies started to form budding organoids. 64-75% (depending on the experiment) of yMaSC colonies evolved as organoids and were maintained and passaged without doxycycline. Also note that direct plating of MaSCs, LD control EGFP-infected, as well as YAP-infected cells, directly into organoid culture conditions did not result in any outgrowth, indicating that the intermediate step in colony culture conditions is required for organoid development. 2 weeks after seeding, organoids were removed from Matrigel, trypsin-dissociated and transferred to fresh Matrigel. Passages were performed in a 1:4-1:8 split ratio every 2 weeks. For analysis, colonies and organoids were recovered from Matrigel as before, and either embedded in OCT medium (PolyFreeze, Sigma) to obtain frozen sections for immunofluorescence or processed for protein or RNA extraction.

For the experiment depicted in Figure S1F, cells were plated into 96-well plate as single cells visually verified and their colony forming capacity was monitored. Based on the fluorescence of EGFP-positive LD cells (negative control cells), we measured that our rate of infection is about 50%.

For *α*- and *β-casein* induction (Figures 3F and S3G), Matrigel-embeded organoids derived from yMaSCs or MaSCs were treated with MG organoid medium supplemented with insulin (10 μg/ml) and dexamethasone (1 μg/ml) in the absence or presence of lactogenic hormone prolactin (5 μg/ml) for 7 days. Organoids were then recovered from Matrigel as before and processed for RNA extraction.

Cleared Mammary Fat Pad Transplantation

For induction of yMaSCs meant for in vivo injection (Figures 3H-J and S3I, J), adherent luminal differentiated cells were transduced for 48 hours with FUW-tetO-wtYAP in combination with stable rtTA- and EGFP-encoding lentiviruses to trace with EGFP fluorescence the generation of transgenic mammary glands from yMaSCs. For this, we mixed 500 μl of each FUdeltaGW-rtTA, pRRL-CMV-GFP and the FUW-tetO-wtYAP viral supernatants, diluted with 1.5 ml of serum-free MG medium with 2X concentrations of supplements, added to each 3 cm dish. Negative control LD cells were transduced with FUW-tetO-EGFP, rtTA and pRRL-CMV-GFP. After infection, cells were treated as before (washed, induced with doxycycline for 7 days in MG medium) and then injected in the cleared fat pads (see below). For the experiment of Figures 3K, L, cells were transduced as above and then cultured as clonal colonies and organoids; the latter were injected in the cleared fat pad of

recipient mice.

Cell aliquots or organoids resuspended in 10 μl PBS/10% Matrigel were injected into the inguinal mammary fat pads of NOD-SCID mice (Charles River), which had been cleared of endogenous mammary epithelium at 3 weeks of age. Animals were then administered doxycycline in the drinking water for 2 weeks and then maintained without doxycycline for additional 8-10 weeks. Transplanted mammary fat pads were examined for gland reconstitution by whole-mount staining, GFP native fluorescence and immunofluorescence on sections from paraffin-embedded biopsies. Only the presence of GFP-positive branched ductal trees with lobules and/or terminal end buds was scored as positive reconstitution. For whole-mount analysis of mammary glands, freshly-explanted glands were fixed in PFA 4% (2 hours) and ethanol 70% (overnight). Glands were rehydrated, stained overnight with hematoxylin, subsequently dehydrated in graded ethanols, cleared by incubation in benzyl-alcohol/benzyl benzoate (1:2; Sigma) and imaged.

Lineage tracing of neurons to yNSCs conversion

For the experiment depicted in Figures 4C-F and S4F, J, we obtained cortical neurons from *Syn1-Cre; R26-CAG-LSL-tdTomato/+* embryos (day 1 and 2 as described in experimental procedures). When indicated, these cells were transduced as described in experimental procedures (day 3). Cells were then treated with AraC/B27 containing medium as before and, after 7 days, switched to doxycycline containing-NSC medium to activate YAP expression and induce yNSC formation from tdTomato-positive neurons. *Syn1-Cre; R26-CAG-LSL-tdTomato/+* neurons transduced with FUdeltaGW-rtTA in combination with FUW-tetO-EGFP or FUW-tetO-YAPS94A never gave rise to any neurospheres and died after few days after transfer to NSC medium. Each embryo genotype was confirmed on tail biopsies post-brain dissociation; as separate negative controls, neurons derived from *R26-CAG-LSL-tdTomato/+* littermates (*Syn1-Cre* negative) were transduced with FUW-tetO-wtYAP and FUdeltaGW-rtTA viral supernatants, and never gave rise to tdTomato-positive yNSCs. These same neurons transduced with FUdeltaGW-rtTA in combination with FUW-tetO-EGFP or FUW-tetO-YAPS94A never gave rise to any neurospheres.

For the experiment depicted in Figure 4G, we obtained hippocampal neurons from *Thy1-Cre; R26-LSL-LacZ/+* embryos (day 1 and 2 as described in experimental procedures). These cells were transduced as described in experimental procedures (day 3). Cells were then treated with AraC/B27 containing medium as before and, after 7 days, switched to doxycycline

containing-NSC medium to activate YAP expression and induce yNSC formation from LacZ-positive neurons. *Thy1-Cre; R26-LSL-LacZ/+* neurons transduced with FUdeltaGW-rtTA in combination with FUW-tetO-EGFP or FUW-tetO-YAPS94A never gave rise to any neurospheres. Each embryo genotype was confirmed on tail biopsies post-brain dissociation; as separate negative controls, neurons derived from *R26-LSL-LacZ/+* littermates (Thy1-Cre negative) were transduced with FUW-tetO-wtYAP and FUdeltaGW-rtTA viral supernatants, and never gave rise to LacZ-positive yNSCs. These same neurons transduced with FUdeltaGW-rtTA in combination with FUW-tetO-EGFP or FUW-tetO-YAPS94A never gave rise to any neurospheres.

To validate the specificity of the neuronal drivers, primary NSCs were derived as below from *Syn1-Cre; R26-CAG-LSL-tdTomato/+* and *Thy1-Cre; R26-LSL-LacZ/+* embryos and we verified the negativity for tdTomato and LacZ, respectively (see Figures 4F, G)

For the experiment depicted in Figures 4H, S4G-I and S5B, we obtained cortical neurons from *Syn1-Cre; R26-LSL-rtTA-IRES-EGFP/+* embryos and hippocampal neurons from *Thy1-Cre; R26-LSL-rtTA-IRES-EGFP/+* (day 1 and 2 as described in experimental procedures). Each embryo genotype was confirmed on tail biopsies post-brain dissociation. These neurons were transduced with FUW-tetO-YAP wt alone (or FUW-tetO-empty vector or FUW-tetO-YAPS94A as negative controls). Cells were then treated with AraC/B27 containing medium as before and, after 7 days, switched to doxycycline containing-NSC medium to activate YAP expression and induce yNSC formation from GFP-positive neurons. For additional negative controls, neurons derived from *R26-LSL-rtTA-IRES-EGFP/+*littermates (*Thy1-Cre* negative) were treated as all the others, and never gave rise yNSCs.

For the experiment with excisable YAP vectors (Figures S4L-N), neurons were transduced (day 3) for 24 hours with LV-tetO-wtYAP-LoxP in combination with LV-CMV-rtTA-LoxP. Neurons were then treated with AraC/B27 containing medium as before and, after 7 days, switched to doxycycline containing-NSC medium to activate YAP expression.

Primary neural stem cells (NSCs) isolation and culture

Neural stem cells (NSCs) were isolated as previously reported (Ray and Gage, 2006) from the telencephalon of C57BL/6J E18 embryos or from mice of the indicated genotype. Telencephalons were minced and digested in trypsin 0.05% for 10 min at 37°C. The cell suspension was treated with DNaseI (Roche) and washed. NSCs were cultured in DMEM/F12 supplemented with 1% N2, 20 ng/ml murine EGF, 20 ng/ml murine bFGF, glutamine and

antibiotics. For passages, neurospheres were dissociated into single cells with TrypLE Express (Life Technologies).

NSCs/yNSCs transfection, infection and differentiation

Prior to transfection with siRNA, yNSCs were plated on fibronectin coated-plate in NSC medium, to allow a 2D culture; the next day, cells were transfected with siRNA and after 24 hours, replated in ultra-low attachment plates to allow neurosphere formation. Neurospheres were counted after 7 days from plating.

For adenoviral infection of wild-type (wt) or double *Yap*$^{fl/fl}$*; Taz*$^{fl/fl}$ NSCs (Figure 5K), single cells were plated in NSC medium containing adeno-Cre on ultra-low attachment plates and allowed to form neurospheres for 7 days.

For lentiviral infection of NSCs (Figure S4K), cells plated on fibronectin coated-plate in NSC medium, to allow a 2D culture; the next day, cells were infected with FUW-tetO-wtYAP and FUdeltaGW-rtTA viral supernatants. Negative controls were provided by neurons transduced with FUdeltaGW-rtTA in combination with FUW-tetO-EGFP. After 24 hours single cells were replated at a density of 20000 cells/well to allow neurosphere formation. Neurospheres were counted after 7 days from plating.

For neuronal differentiation (Choi et al., 2014), NSCs or yNSCs were cultured over a thin Matrigel layer. Differentiation medium was Neurobasal supplemented with 1X B27, glutamine.

For astrocyte differentiation (Bonaguidi et al., 2005), NSCs or yNSCs were plated on fibronectin coated-plate in NSC medium, to allow a 2D culture. The next day, medium was changed to DMEM (Life Technologies) containing 25 ng/ml LIF, 25 ng/ml BMP4, glutamine, and antibiotics for 2 weeks.

For oligodendrocyte differentiation (Hsieh et al., 2004), NSCs or yNSCs were plated on fibronectin coated-plate in NSC medium, to allow a 2D culture. The next day, medium was changed to Neurobasal (Life Technologies) containing 1x B27, 500 ng/ml IGF, 30 ng/ml T3, glutamine, and antibiotics for 2 weeks.

NSCs transplantation

P0 CD1 mice pups were used for cell transplantations. Pups were anesthetized by hypothermia (3 minutes) and fixed on ice-cold block during cell injection. Cells were resuspended in ice-cold HBSS ($5x10^4$ cells/µl) and injected into both hemispheres of neonatal mice with a 5µl-volume Hamilton syringe (2µl/injection). One month after the procedures, the

grafted animals were perfused with PBS and 4%PFA, and the brains were excised and processed for immunofluorescence.

Lineage tracing of pancreatic exocrine acinar cells

For the experiment depicted in Figures 6E-F (see also scheme in Figure S6I), we obtained acinar explants from 6 week-old *Ptf1a-CreERTM; R26-LSL-rtTA-IRES-EGFP/+; tetO-YAP*^{S127A} mice (Pan et al., 2013). Mice with this genotype were given tamoxifen by three daily i.p. injections of a 10 mg/ml solution in corn oil. 1 week after the last tamoxifen injection, mice were sacrificed and pancreas was dissociated to obtain *rtTA-IRES-EGFP*$^{+}$-labeled exocrine acinar cells. Primary pancreatic acinar cells were isolated and cultured as described in experimental procedures. For induction of pancreatic organoids, acinar explants were treated with 2 μg/ml doxycycline.

Matrigel culture of yDucts organoids

To show the self-renewal capacity of pancreatic organoids independently of exogenous YAP supply (i.e, independently of doxycycline administration), organoids were recovered from Matrigel or collagen cultures, trypsinized to obtain a single cell suspension and re-seeded in 100% Matrigel covered with pancreatic organoid medium. For analysis, organoids were recovered from Matrigel as before and processed for immunofluorescence or for protein or RNA extraction.

For the differentiation experiments shown in Figure S6R, yDucts were removed from Matrigel, trypsin-dissociated and seeded as single cells in Matrigel- coated (1:50) chamber slides. Cells were expanded in DMEM supplemented with 0.5% BSA, 1% ITS-X and 1x N2 and 50 ng/ml EGF and antibiotics for 5 days. For differentiation, cells were switched to DMEM/F12 supplemented with 1% ITS-X, 10 ng/ml bFGF, 10 mM nicotinamide, 50 ng/ml Exendin-4 and 10 ng/ml BMP4 and antibiotics for further 8 days. Cells were fixed in 4% PFA at Day 0 or Day 8 of differentiation and processed for immunofluorescence.

Culture of pancreatic ductal organoids (Ducts)

For culture of pancreatic duct-derived organoids, pancreatic ducts were isolated from the bulk of the pancreas as previously described(Huch et al., 2013) with minor modifications. The whole pancreas of 6- to 9-week-old mice of the indicated genotypes was grossly minced and digested by collagenase/dispase dissociation: DMEM medium (Life Technologies)

supplemented with collagenase type XI 0.012% (w/v) (Sigma), dispase 0.012% (w/v) (Life Technologies), 1% FBS (Life Technologies) and antibiotics at 37°C for 1 hour. Isolated pancreatic duct fragments were hand-picked under a dissecting microscope, carefully washed in DMEM medium and embedded in 100% Matrigel. After Matrigel formed a gel, pancreatic organoid medium (Advanced DMEM/F12 supplemented with 1x B27, 1.25mM N-Acetylcysteine, 10 nM gastrin, 50 ng/ml murine EGF, 100 ng/ml human Noggin, 100 ng/ml human FGF10, 10 mM Nicotinamide, 1 μg/ml R-Spondin1 and antibiotics) was added. Ductal fragments rapidly expanded to form cyst-like organoids within 5 days. Organoids were removed from Matrigel by incubation in ice cold HBSS, dissociated with trypsin 0.05% for 30 min to obtain a single cells suspension and reseeded in 100% fresh Matrigel. Organoid cultures were maintained for at least 9 months passaging every 10 days. For analysis, organoids were recovered from Matrigel and processed for immunofluorescence or for protein or RNA extraction.

For the experiments depicted in Figure S6C, pancreatic duct fragments were isolated from 9 weeks old $Yap^{fl/fl}$; $Taz^{fl/fl}$ mice, embedded in 100% Matrigel and cultured as above. Organoids were passaged once every 10 days. After at least 3 months of culture, organoids were removed from Matrigel by incubation in ice cold HBSS, trypsin-dissociated and transduced with adenovirus encoding for CRE recombinase to induce Yap/Taz knockout (or with GFP-encoding adenovirus as control). Single cells were resuspended in 2 ml Advanced DMEM/F12, transduced for 2 hours at 37°C with adenovirus, washed in Advanced DMEM/F12 and seeded in 100% Matrigel. After Matrigel formed a gel, cells were maintained in pancreatic organoid medium and organoid formation capacity was morphologically monitored over a period of 10 days. Pancreatic ductal organoids obtained from *wt* mice were used as additional controls and treated as above.

Single-cell gene expression analysis during the conversion of pancreatic acinar cells to yDucts

For the experiment depicted in Figure 6G, we dissociated and harvested single cells from pancreatic cultures of the *R26-rtTA; tetO-YAP S127A* genotype during the YAP-induced conversion from acinar cells to yDucts, that is at: day 0 = starting acini (without doxycycline); day 2 = cultures that have experienced 48 hours of doxycycline; day 4 = cultures that have experienced 96 hours of doxycycline; yDucts (n= 12/13 each time point). Single cells were collected by pipetting into 200 μl- conical tubes and processed for total RNA extraction,

retro-transcription and pre-amplification with Single Cell-to-CT kit (Ambion), according to manufacturer's protocol. Specific target amplification products were then processed for qRT-PCR analysis on QuantStudio 6 Flex Real-time PCR System, 384-well (Applied Biosystems) with the following Taqman Gene Expression Assays: *18S* (Hs99999901_s1), *Amy2a5* (Mm02342486_mH), *Ptf1a* (Mm00479622_m1), *Nestin* (Mm00450205_m1), *Krt19* (Mm00492980_m1), *Sox9* (Mm00448840_m1), *Car2* (Mm00501576_m1), *Pdx1* (Mm00435565_m1), *Ctgf* (Mm01192933_g1), *Myc* (Mm00487804_m1), *Ccnd1* (Mm00432359_m1). Expression levels were normalized to *18S-rRNA* for each sample.

Immunofluorescence and microscopy

Immunofluorescences on PFA-fixed samples were performed as previously described(Cordenonsi et al., 2011). Briefly, samples were fixed 10 min at room temperature with 4% PFA solution. Slides were permeabilized 10 min at RT with PBS 0.3% Triton X-100, and processed for immunofluorescence using the following conditions: blocking in 10% Goat Serum (GS) in PBS 0.1% Triton X-100 (PBST) for 1 hr followed by incubation with primary antibodies (diluted in 2% GS in PBST) overnight at 4°C, four washes in PBST and incubation with secondary antibodies (1:200 in 2% GS in PBST) for 2 hours at room temperature. Samples were counterstained with ProLong-DAPI (Molecular Probes, Life Technologies) to label cell nuclei.

For immunofluorescence on mammary colonies and organoids (Figures 2B-C, 3B-E, S2, S3C-F), outgrowths freshly recovered from Matrigel were embedded in OCT tissue-freezing medium (PolyFreeze, Sigma) and frozen on dry ice. 8 µm cryostat sections for all types of organoids were cut at –20 °C. Sections were mounted on glass slides and dried for at least 30 min. The sections were then fixed with 4% formaldehyde for 10 min. After washing with PBS the sections were processed as described above. For immunofluorescence on pancreatic organoids or acini (Figures 6H and S6A, E), pancreatic acini and organoids were fixed overnight in PBS 4% PFA at 4°C, permeabilized with two washes in PBS 0.5% NP40 for 20 minutes at 4°C, followed by one wash in PBS 0.3% Triton X-100 for 20 minutes at room temperature. After two washes in PBS 0.1% Triton X-100 (PBST) for 15 minutes at room temperature, acini or organoids were blocked with two washes in PBST 10% GS for 1 hour at room temperature, and incubated overnight with primary antibodies. The following day, cells were washed twice in PBST 2% GS for 15 minutes at 4°C, and five more times in PBT 2% GS for 1 hour at 4°C. Secondary antibodies were incubated overnight. The third day, cells were washed five times in PBST for 15 minutes, incubated 20 min with DAPI solution and

mounted in glycerol.

For immunofluorescence on mammary and brain tissue, biopsies were fixed with PFA, paraffin-embedded and cut in 10 μm-thick sections. Sections were re-hydrated and antigen retrieval was performed by incubation in citrate buffer 0.01 M pH 6 at 95°C for 20 minutes. Slides were then permeabilized (10 min at RT with PBS 0.3% Triton X-100 for mammary sections and 10 min at RT with PBS 1% Triton X-100 for brain sections) and processed as described above.

Primary antibodies: anti-YAP (4912; 1:25) polyclonal antibody, anti-CNPase (5664S; 1:100) polyclonal antibody, anti-SOX2 (4900; 1:50) monoclonal antibody were from Cell Signaling Technology. anti-TAZ (anti-WWTR1, HPA007415; 1:25) polyclonal antibody, anti-α-SMA (A2547; 1:400) mouse monoclonal antibody and anti-amylase (A8273: 1:200) rabbit polyclonal antibody were from Sigma. anti-TUJ1 (anti β-III-tubulin; MMS435P-100; 1:500) mouse monoclonal antibody was from Covance. Anti-Synaptophysin (M0776; 1:200) mouse monoclonal antibody and anti-GFAP (Z0334; 1:1000) rabbit polyclonal antibody was from Dako. anti-Nestin (MAB353; 1:300) mouse monoclonal antibody and anti-SOX9 (AB5535; 1:200) and anti-NEUN (ABN78; 1:1000) rabbit polyclonal antibodies were from Millipore. anti-E-cadherin (610181; 1:1000) and anti-MASH1 (556604; 1:100) monoclonal antibody was from BD Biosciences. anti-K14 (Ab7800; 1:100) mouse monoclonal antibody, anti-NEUN (Ab177487; 1:100) rabbit monoclonal antibody, anti-K8 (Ab14053; 1:100) chicken polyclonal antibody and anti-GFP (Ab13970; 1:100) polyclonal antibody were from Abcam. anti-GFP (A6455; 1:100) rabbit serum was from Life Technologies. Anti-YAP (63.7; sc-101199; 1:200) mouse monoclonal antibody, anti-SV2a (E-8; sc-376234; 1:200) mouse monoclonal antibody, anti-p63 (H137, sc-8343; 1:50) polyclonal antibody and anti-Vimentin (Vim C-20, sc-7557-R; 1:100) rabbit polyclonal antibody were from Santa Cruz. anti-TAU (1:100) rabbit polyclonal antibody was from Axell. Anti-Ki67 (M3060; 1:100) rabbit monoclonal antibody was from Spring Bioscience. K19 was detected using the monoclonal rat anti-*Troma-III* antibody (DSHB; 1:50). Alexa-conjugated secondary antibodies (Life Technologies): Alexa-Fluor-488 donkey anti-mouse IgG (A21202); Alexa Fluor-568 goat anti-mouse IgG (A11031); Alexa-Fluor-647 donkey anti-mouse (A31571); Alexa Fluor-488 goat anti-mouse IgG_{2a} (A21131), Alexa Fluor-647 goat anti-mouse IgG_1 (A21240), Alexa Fluor-488 donkey anti-rabbit IgG (A21206), Alexa-Fluor-568 goat anti-rabbit IgG (A11036), Alexa-Fluor-647 donkey anti-rabbit IgG (A31573); Alexa Fluor-555 goat anti-chicken IgG (A21437). Goat anti-rat Cy3 (112-165-167) was from Jackson Immunoresearch.

For X-gal staining (Figure 4G), samples were permeabilized in PBS/NP-40 0.02%, fixed 1 hour in PFA 4% in PBS, washed twice in PBS/NP-40 0.02% and stained with the staining solution (X-gal (Sigma, B4252) 25 μg/ml, 4 mM potassium ferricyanide crystalline, 4 mM potassium ferricyanide trihydrate, 2mM $MgCl_2$, 0.02% NP-40 in PBS).

Confocal images were obtained with a Leica TCS SP5 equipped with a CCD camera. Bright field and native-GFP (or tdTomato) images were obtained with a Leica DM IRB inverted microscope equipped with a CCD camera (Leica DFC 450C). Live cell imaging was performed with a A1Rsi+ laser scanning confocal microscope (Nikon) equipped with NIS-Elements Advanced Research Software.

Western blot

Western blots were carried out as described in Ref. (Cordenonsi et al., 2011). Anti-YAP/TAZ (63.7; sc-101199) and anti-p63 (4A4; sc-8431) monoclonal antibodies were from Santa Cruz. anti-GAPDH (MAB347) monoclonal antibody was from Millipore. Anti-K14 (Ab7800) mouse monoclonal antibody and anti-K8 (Ab14053) chicken polyclonal antibody were from Abcam.

Quantitative Real-Time PCR (qRT-PCR)

Cells or tissues were harvested in TriPure (Roche) for total RNA extraction, and contaminant DNA was removed by DNase treatment. qRT-PCR analyses were carried out on retrotranscribed cDNAs with Rotor-Gene Q (Qiagen) thermal cycler and analyzed with Rotor-Gene Analysis6.1 software. Expression levels are always given relative to *Gapdh,* except for Figures S6B and S6Q in which expression levels were normalized to *18-S rRNA*. PCR oligo sequences for mouse samples are listed in our website http://www.bio.unipd.it/piccolo/protocols_and_tools.html.

Microarray experiments

For microarray experiments, Mouse Genome 430 2.0 arrays (Affymetrix, Santa Clara, CA, USA) were used. Total RNA was extracted using TriPure (Roche) from:

1) luminal differentiated mammary cells (3 replicates), organoids derived from yMaSCs (3 replicates), and MaSCs (3 replicates);

2) cortical neurons (3 replicates), yNSCs (from YAP wild type-transduced cortical neurons, passage 2; 3 replicates), and native NSCs (3 replicates);

3) pancreatic exocrine acini (4 replicates), yDucts (passage 10; 4 replicates), and Ducts (passage 10; 4 replicates).

RNA quality and purity were assessed on the Agilent Bioanalyzer 2100 (Agilent Technologies, Waldbronn, Germany); RNA concentration was determined using the NanoDrop ND-1000 Spectrophotometer (NanoDrop Technologies Inc.). RNA was then treated with DNaseI (Ambion). In vitro transcription, hybridization and biotin labeling were performed according to Affymetrix 3'IVT protocol (Affymetrix). As control of effective gene modulation and of the whole procedure, we monitored the expression levels of tissue-specific markers of differentiated cells or stem/progenitors by qRT-PCR prior to microarray hybridization and in the final microarray data.

All data analyses were performed in R (version 3.1.2) using Bioconductor libraries (BioC 3.0) and R statistical packages. Probe level signals were converted to log2 expression values using robust multi-array average procedure RMA(Irizarry et al., 2003) of Bioconductor *affy* package. Raw data are available at Gene Expression Omnibus under accession number GSE70174.

Global unsupervised clustering was performed using the function *hclust* of R *stats* package with Pearson correlation as distance metric and average agglomeration method. Gene expression heatmaps have been generated using the function *heatmap.2* of R *gplots* package after row-wise standardization of the expression values. Before unsupervised clustering, to reduce the effect of noise from non-varying genes, we removed those probe sets with a coefficient of variation smaller than the 90th percentile of the coefficients of variation in the entire dataset. The filter retained 4511 probe sets that are more variable across samples in any of the 3 subsets (i.e., mammary, neuron, and pancreatic).

Differentially expressed genes were identified using Significance Analysis of Microarray algorithm coded in the *samr* R package (as in Ref. (Tusher et al., 2001)). To identify differentially expressed genes, we selected those probe sets with an FDR ≤ 1%.

GO terms associated to genes coherently upregulated to Ducts and yDucts in comparison with acinar cells included RNA processing, positive regulation of cell cycle and control of cell death.

Mice

C57BL/6J mice and NOD-SCID mice were purchased from Charles River. Transgenic lines used in the experiments were gently provided by: Duojia Pan(Zhang et al., 2010) (*Yap1*$^{fl/fl}$ and *R26-LSL-LacZ*); Cedric Blanpain (*K8-CreERT2/R26-LSL-YFP)*(Van Keymeulen et al., 2011);

Pierre Chambon (*K14-CreERT2*)(Li et al., 2000); Doron Merckler (*Thy1.2-Cre*)(Dewachter et al., 2002); Ivan De Curtis and Riccardo Brambilla (*Syn1-Cre*)(Zhu et al., 2001); Giorgio Carmignoto (*R26-CAG-LSL-tdTomato*)(Madisen et al., 2010); Fernando Camargo (*tetO-YAP^{S127A}*)(Camargo et al., 2007). *Taz$^{fl/fl}$* and double *Yap$^{fl/fl}$; Taz$^{fl/fl}$* conditional knock-out mice were as described in Ref. (Azzolin et al., 2014). *Ptf1a-CreERTM* (stock #019378), *R26-LSL-rtTA-IRES-EGFP* (stock #005670) and *R26-rtTAM2* mice (stock #006965) were purchased from The Jackson Laboratory. Animals were genotyped with standard procedures(Morsut et al., 2010) and with the recommended set of primers. Animal experiments were performed adhering to our institutional guidelines as approved by OPBA and authorized by the Ministry of Health.

To obtain *Thy1-Cre; R26-LSL-LacZ/+* mice, we crossed *Thy1-Cre* hemizygous males with *R26-LSL-LacZ/LSL-LacZ* females. Littermate embryos derived from these crossings were harvested at E18-19 and kept separate for neurons/NSCs derivation; genotypes were confirmed on embryonic tail biopsies.

To obtain *Thy1-Cre; R26-LSL-rtTA-IRES-EGFP/+* mice, we crossed *Thy1-Cre* hemizygous mice with *R26-LSL-rtTA-IRES-EGFP/LSL-rtTA-IRES-EGFP* mice. Littermate embryos derived from these crossings were harvested at E18-19 and kept separate for neurons derivation; genotypes were confirmed on embryonic tail biopsies.

To obtain *Syn1-Cre* lineage tracing studies, we used *Syn1-Cre* hemizygous females (as transgene expression in male mice results in germline recombination(Rempe et al., 2006)) with *R26-LSL-rtTA-IRES-EGFP* or with *R26-CAG-LSL-tdTomato* homozygous males. Littermate embryos derived from these crossings were harvested at E18-19 and kept separate for neurons derivation; genotypes were confirmed on embryonic tail biopsies.

To obtain *R26-rtTAM2/+ ; tetO-YAP^{S127A}* mice, we crossed *R26-rtTAM2/+* mice with *tetO-YAP^{S127A}* mice. *R26-rtTAM2/+* littermates were used as negative control.

To obtain *Ptf1a-CreERTM; R26-LSL-rtTA-IRES-EGFP/+; tetO-YAP^{S127A}* mice, we crossed *Ptf1a-CreERTM; R26-LSL-rtTA-IRES-EGFP/LSL-rtTA-IRES-EGFP* mice with *tetO-YAP^{S127A}* mice. *Ptf1a-CreERTM; R26-LSL-rtTA-IRES-EGFP/+* littermates were used as negative control.

SUPPLEMENTAL REFERENCES

Aragona, M., Panciera, T., Manfrin, A., Giulitti, S., Michielin, F., Elvassore, N., Dupont, S., and Piccolo, S. (2013). A mechanical checkpoint controls multicellular growth through YAP/TAZ regulation by actin-processing factors. Cell *154*, 1047-1059.

Azzolin, L., Panciera, T., Soligo, S., Enzo, E., Bicciato, S., Dupont, S., Bresolin, S., Frasson, C., Basso, G., Guzzardo, V., *et al.* (2014). YAP/TAZ incorporation in the beta-catenin destruction complex orchestrates the Wnt response. Cell *158*, 157-170.

Azzolin, L., Zanconato, F., Bresolin, S., Forcato, M., Basso, G., Bicciato, S., Cordenonsi, M., and Piccolo, S. (2012). Role of TAZ as mediator of Wnt signaling. Cell *151*, 1443-1456.

Bonaguidi, M.A., McGuire, T., Hu, M., Kan, L., Samanta, J., and Kessler, J.A. (2005). LIF and BMP signaling generate separate and discrete types of GFAP-expressing cells. Development *132*, 5503-5514.

Camargo, F.D., Gokhale, S., Johnnidis, J.B., Fu, D., Bell, G.W., Jaenisch, R., and Brummelkamp, T.R. (2007). YAP1 increases organ size and expands undifferentiated progenitor cells. Curr Biol *17*, 2054-2060.

Choi, S.H., Kim, Y.H., Hebisch, M., Sliwinski, C., Lee, S., D'Avanzo, C., Chen, H., Hooli, B., Asselin, C., Muffat, J., *et al.* (2014). A three-dimensional human neural cell culture model of Alzheimer's disease. Nature *515*, 274-278.

Cordenonsi, M., Zanconato, F., Azzolin, L., Forcato, M., Rosato, A., Frasson, C., Inui, M., Montagner, M., Parenti, A.R., Poletti, A., *et al.* (2011). The Hippo transducer TAZ confers cancer stem cell-related traits on breast cancer cells. Cell *147*, 759-772.

Dewachter, I., Reverse, D., Caluwaerts, N., Ris, L., Kuiperi, C., Van den Haute, C., Spittaels, K., Umans, L., Serneels, L., Thiry, E., *et al.* (2002). Neuronal deficiency of presenilin 1 inhibits amyloid plaque formation and corrects hippocampal long-term potentiation but not a cognitive defect of amyloid precursor protein [V717I] transgenic mice. The Journal of neuroscience : the official journal of the Society for Neuroscience *22*, 3445-3453.

Hockemeyer, D., Soldner, F., Cook, E.G., Gao, Q., Mitalipova, M., and Jaenisch, R. (2008). A drug-inducible system for direct reprogramming of human somatic cells to pluripotency. Cell Stem Cell *3*, 346-353.

Hsieh, J., Aimone, J.B., Kaspar, B.K., Kuwabara, T., Nakashima, K., and Gage, F.H. (2004). IGF-I instructs multipotent adult neural progenitor cells to become oligodendrocytes. The Journal of cell biology *164*, 111-122.

Huch, M., Bonfanti, P., Boj, S.F., Sato, T., Loomans, C.J., van de Wetering, M., Sojoodi, M., Li, V.S., Schuijers, J., Gracanin, A.*, et al.* (2013). Unlimited in vitro expansion of adult bi-potent pancreas progenitors through the Lgr5/R-spondin axis. The EMBO journal *32*, 2708-2721.

Irizarry, R.A., Hobbs, B., Collin, F., Beazer-Barclay, Y.D., Antonellis, K.J., Scherf, U., and Speed, T.P. (2003). Exploration, normalization, and summaries of high density oligonucleotide array probe level data. Biostatistics (Oxford, England) *4*, 249-264.

Li, M., Indra, A.K., Warot, X., Brocard, J., Messaddeq, N., Kato, S., Metzger, D., and Chambon, P. (2000). Skin abnormalities generated by temporally controlled RXRalpha mutations in mouse epidermis. Nature *407*, 633-636.

Madisen, L., Zwingman, T.A., Sunkin, S.M., Oh, S.W., Zariwala, H.A., Gu, H., Ng, L.L., Palmiter, R.D., Hawrylycz, M.J., Jones, A.R.*, et al.* (2010). A robust and high-throughput Cre reporting and characterization system for the whole mouse brain. Nature neuroscience *13*, 133-140.

Maherali, N., Ahfeldt, T., Rigamonti, A., Utikal, J., Cowan, C., and Hochedlinger, K. (2008). A high-efficiency system for the generation and study of human induced pluripotent stem cells. Cell Stem Cell *3*, 340-345.

Morsut, L., Yan, K.P., Enzo, E., Aragona, M., Soligo, S.M., Wendling, O., Mark, M., Khetchoumian, K., Bressan, G., Chambon, P.*, et al.* (2010). Negative control of Smad activity by ectodermin/Tif1gamma patterns the mammalian embryo. Development *137*, 2571-2578.

Pan, F.C., Bankaitis, E.D., Boyer, D., Xu, X., Van de Casteele, M., Magnuson, M.A., Heimberg, H., and Wright, C.V. (2013). Spatiotemporal patterns of multipotentiality in Ptf1a-expressing cells during pancreas organogenesis and injury-induced facultative restoration. Development *140*, 751-764.

Ray, J., and Gage, F.H. (2006). Differential properties of adult rat and mouse brain-derived neural stem/progenitor cells. Molecular and cellular neurosciences *31*, 560-573.

Rempe, D., Vangeison, G., Hamilton, J., Li, Y., Jepson, M., and Federoff, H.J. (2006). Synapsin I Cre transgene expression in male mice produces germline recombination in progeny. Genesis *44*, 44-49.

Tusher, V.G., Tibshirani, R., and Chu, G. (2001). Significance analysis of microarrays applied to the ionizing radiation response. Proceedings of the National Academy of Sciences of the United States of America *98*, 5116-5121.

Van Keymeulen, A., Rocha, A.S., Ousset, M., Beck, B., Bouvencourt, G., Rock, J., Sharma, N., Dekoninck, S., and Blanpain, C. (2011). Distinct stem cells contribute to mammary gland development and maintenance. Nature *479*, 189-193.

Zhang, N., Bai, H., David, K.K., Dong, J., Zheng, Y., Cai, J., Giovannini, M., Liu, P., Anders, R.A., and Pan, D. (2010). The Merlin/NF2 tumor suppressor functions through the YAP oncoprotein to regulate tissue homeostasis in mammals. Dev Cell *19*, 27-38.

Zhao, B., Ye, X., Yu, J., Li, L., Li, W., Li, S., Yu, J., Lin, J.D., Wang, C.-Y., Chinnaiyan, A.M.*, et al.* (2008). TEAD mediates YAP-dependent gene induction and growth control. Genes & development *22*, 1962-1971.

Zhu, Y., Romero, M.I., Ghosh, P., Ye, Z., Charnay, P., Rushing, E.J., Marth, J.D., and Parada, L.F. (2001). Ablation of NF1 function in neurons induces abnormal development of cerebral cortex and reactive gliosis in the brain. Genes & development *15*, 859-876.
